# CRISPR and compound screens in a novel ex vivo tissue model identify DDR1 and ETA as regulators of cancer cell invasion

**DOI:** 10.1186/s11658-026-00936-6

**Published:** 2026-05-06

**Authors:** Junnan Liu, Wencheng Jiang, Xue Wang, Anca Azoitei, Hengchuan Liu, Gregoire Najjar, Kuangzheng Liu, Michael Karl Melzer, Stephan Stilgenbauer, Mohamed Elati, Martin D. Burkhalter, Melanie Philipp, Felix Wezel, Friedemann Zengerling, Christian Bolenz, Cagatay Günes

**Affiliations:** 1 Department of Urology, Ulm University Hospital, Ulm, Germany; 2 Department of Internal Medicine III, Ulm University Hospital, Ulm, Germany; 3 CANTHER, ONCOLille Institute, University of Lille, CNRS UMR 1277, Inserm U9020, Lille, France; 4 Department of Experimental and Clinical Pharmacology and Pharmacogenomics, Section of Pharmacogenomics, Eberhard-Karls-University Tübingen, Tübingen, Germany; 5 Present Address: Department of Urology, Mayo Clinic College of Medicine and Science, Rochester, MN USA; 6 Present Address: Department of Molecular Pharmacology and Therapeutics, Columbia University Irving Medical Center, New York, NY USA; 7 Present Address: Mol. Pharmacology and Exp. Therapeutics, Mayo Clinic College of Medicine and Science, Rochester, MN USA

**Keywords:** Bladder cancer, NMIBC, MIBC, Invasion, Metastasis, Ex vivo tissue model, Small-compound library, CRISPR-Cas9 screening

## Abstract

**Background:**

Bladder cancer (BC) can be characterized clinically as either non-muscle-invasive (NMIBC) or muscle-invasive (MIBC). While NMIBC generally has a favorable prognosis, MIBC is characterized by high morbidity and mortality. Understanding the molecular determinants of tumor invasion is critical, yet research is hampered by the limitations of current experimental models. Standard assays such as the Boyden chamber lack physiological complexity, while porcine bladder models suffer from tissue contamination and genetic variability. There is an urgent need for reliable models that mimic the intact tissue architecture.

**Methods:**

We established a unique ex vivo tissue invasion model (EXTIM) to evaluate the invasive capacity of BC cells within a largely intact tissue context, using freshly prepared bladders from mice. The invasiveness of human BC cells (RT4, T24, UMUC3) and the immortal urothelial cell strain (Y235T) was comparably evaluated using EXTIM, the Boyden chamber, and porcine models. Gene knockdown or ectopic expression of GJB3 or ORP3 indicated the suitability of EXTIM to investigate the impact of specific factors on tumor cell invasion. To identify novel genetic regulators of cell invasion, we combined EXTIM with a genome-wide clustered regularly interspaced short palindromic repeats (CRISPR)-Cas9 knockout screen. Additionally, we utilized the EXTIM to perform a pharmacological screen of a small molecule library comprising 90 substances to identify compounds capable of suppressing BC cell dissemination.

**Results:**

Importantly, by combining EXTIM with genomewide CRISPR-Cas9 screening, we identified several candidate genes involved in BC progression. Notably, discoidin domain receptor tyrosine kinase 1 (DDR1) was identified as a functional inhibitor of tumor cell invasion. Furthermore, the small-molecule screen revealed that PD-156707, a selective antagonist of the endothelin receptor A (ETA), significantly suppresses cancer cell invasion within the EXTIM environment.

**Conclusions:**

EXTIM serves as a robust and physiologically relevant tool for assessing tumor cell invasion and migration under ex vivo conditions. EXTIM can be used to identify factors involved in the progression of invasive BC by high-throughput genetic screenings in an ex vivo organ culture system, by culturing cells after transmigration through the bladder tissue. Moreover, the impact of specific genetic factors in the process of tumor cell dissemination can be assessed by placing bladders from genetically modified mice into the EXTIM.

**Graphical abstract:**

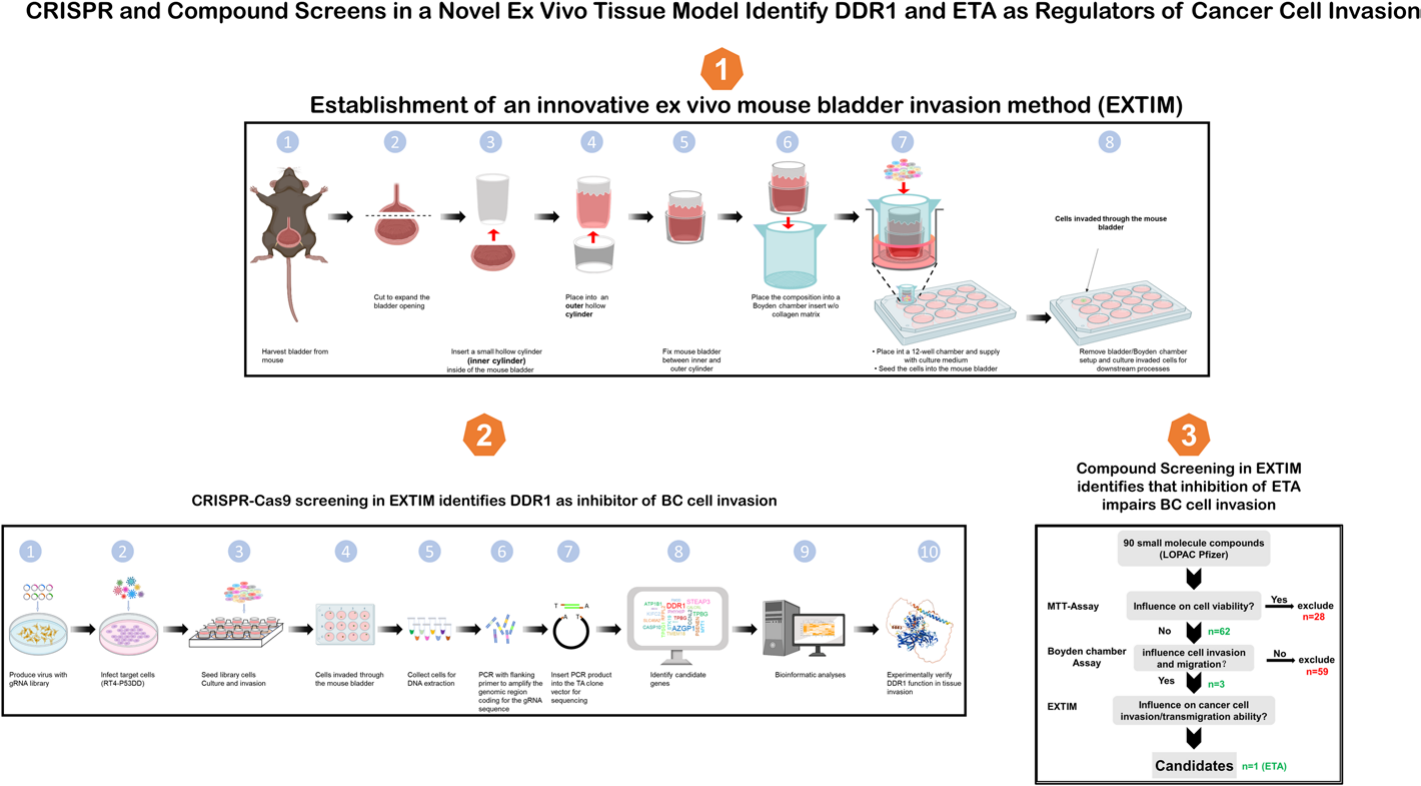

**Supplementary Information:**

The online version contains supplementary material available at 10.1186/s11658-026-00936-6.

## Background

Bladder cancer (BC) is a prevalent malignancy worldwide, with an estimated 614,298 new cases and 220,596 deaths reported in 2024 [1]. BC originates primarily from the urothelial cells lining the bladder lumen and is classified into non-muscle-invasive bladder cancer (NMIBC) and muscle-invasive bladder cancer (MIBC), which differ in clinical behavior, prognosis, and molecular characteristics, especially in terms of cell migration, invasion, and metastasis [2, 3]. The 5-year overall survival (OS) rate of patients with NMIBC is approximately 90%. Unfortunately, the 5-year OS rate of patients decreases to approximately 50% when BC progresses to MIBC [4]. Notably, the OS rate decreases to less than 5% for patients with metastatic BC. Critically, metastasis accounts for approximately 90% of cancer deaths and is the primary driver of recurrence and mortality across all cancers [5]. The first critical step of the metastatic cascade involves the migration of tumor cells from the primary site and their invasion into surrounding tissues, where they transmigrate to distant sites across tissue barriers [6]. Therefore, understanding the mechanisms underlying cancer cell migration and invasion is crucial for improving BC therapy.

The Boyden chamber, a classical method, is widely used for investigating cell invasive and migratory capacities. In this well-established model, Matrigel, which mimics the invasion barrier during cell invasion, is applied to the insert to replace the extracellular matrix (ECM) [7]. However, Matrigel is composed of laminin, collagen IV, heparan sulfate proteoglycans, entactin/nidogen, and a number of growth factors, which represent only a portion of the protein layer found in the basal layer of the bladder. Most importantly, the Boyden chamber setup does not account for the three-dimensional nature of tissue matrices. Consequently, the Boyden chamber system does not fully replicate the actual conditions encountered during cell invasion. Recently, the use of ex vivo models, e.g., culture pieces from rat or porcine bladder or human ureter [8–10], has emerged as a more accurate way to simulate the bladder microenvironment. These tissues contain all the layers found in the human bladder and can closely resemble real conditions during cancer cell invasion. One important limitation of all these models, however, is their suitability for high-throughput screening (HTS) analyses. Genetic screening is a crucial tool in biomedical research for identifying novel pivotal genes and elucidating the mechanisms underlying various biological processes. It has been extensively employed to reveal critical gene functions, identify drug targets, and clarify the mechanisms driving diverse biological processes. This could involve measuring changes in invasion, migration, cell proliferation, drug sensitivity, or other relevant phenotypes. By analyzing the distribution of mutations across the genome, novel genes associated with the phenotype can be identified.

The development of ex vivo models that accurately simulate BC invasion and that allow HTS application can contribute to the identification of key factors involved in BC progression. In the present study, we established a novel BC invasion model in the mouse bladder that effectively recapitulates the invasive process during BC progression. Notably, this model efficiently distinguishes cells with different invasive capacities. By combining CRISPR-Cas9 screening with this ex vivo bladder tissue invasion model, we identified *DDR1* as a top candidate gene potentially regulating BC cell migration and invasion. Moreover, we screened a small-compound library comprising 90 substances developed by Pfizer in combination with EXTIM and identified the endothelin receptor (ETA) as a potential promoter of cancer cell invasion.

In conclusion, EXTIM is an innovative experimental tool for studying critical aspects of cancer cell migration and invasion and holds promise for identifying therapeutic targets and advancing our understanding of the metastatic cascade during the progression of BC.

## Materials and methods

### Mice

Seventeen- to 24-week-old male C57BL/6 J mice were purchased from Jackson Laboratory (Bar Harbor, ME, USA). The experimental procedures were conducted according to the ethical guidelines for the care and use of laboratory animals according to the German Animal Welfare Act and the Directive of the European Union on the Protection of Animals Used for Scientific Purposes (EU Directive 2010/63/EU). Every effort was made to decrease the number of animals used and to reduce animal suffering.

### Establishment of the ex vivo tissue invasion model (EXTIM)

Following excision, the bladder was separated from the kidney and placed into a 1.5-mL vial tube with 1 mL of Roswell Park Memorial Institute (RPMI) 1640 (cat. no. 11875093, Gibco-Thermo Fisher, Waltham, USA) supplemented with 10% fetal calf serum (FCS) (cat. no. AC-SM-0190, Anprotec, Bruckberg, Germany) and with 1% penicillin/streptomycin (P/S) (cat. no. P06-07100, PAN Biotech, Aidenbach, Germany) and kept on ice for further procedures. The mouse bladder was cut transversely at the neck to generate a larger opening, and a hollow cylinder 3 mm in diameter, which was cut from CellCrown™ eight-well strips (cat. no. C00005S, Scaffdex, Tampere, Finland), was placed inside the mouse bladder. This assembly was then placed in another cylinder with a larger diameter (6 mm), which was also cut from the CellCrown™ eight-well strips (cat. no. C00005S, Scaffdex, Tampere, Finland). The sandwich composition was 5.5 mm in height, 2.7 mm in inner diameter, and 6 mm in outer diameter. The inner space of the mouse bladder lumen for seeding cells can afford a 170–200 µL volume of cell suspension in total. The EXTIM structure was then constructed by placing the sandwich composition on a 12-well insert (cat. no. 83.3931.800, Sarstedt, Nümbrecht, Germany) with seeded cells from the opening top of the inner cylinder. The EXTIM structure was maintained in a 12-well plate supplemented with 1 mL of complete medium on the plate bottom and maintained in a humidified atmosphere at 37 °C with 5% carbon dioxide (CO_2_). Three EXTIM structures were placed in one 12-well plate for further maintenance and to monitor invasion. For EXTIM maintenance and invasion monitoring, every 2 days, EXTIM structures were moved into the next line of wells containing 1 mL of fresh medium. After three transfers in one 12-well plate, the EXTIM structures were transferred into a new 12-well plate, and the old 12-well plate was further cultured for 14 days to monitor the growth of potentially invaded cells. Finally, for documentation of invading cells, the plate was treated with 5% glutaraldehyde solution (cat. no. 340855, Sigma-Aldrich, St. Louis, USA) for 15 min and subsequently stained with 0.2% Crystal Violet solution (cat. no. V5262, Sigma-Aldrich, St. Louis, USA) for 30 min. For the quantification and the evaluation of the transmigratory capacity of cells in the functional experiments (as shown in Figs. 2C, F, G, 3C, and 6K, M), cells in the bottom of a culture well were visualized with Crystal Violet staining. To identify the candidate genes in the CRISPR-Cas9 screening experiments (Fig. 4B), transmigrated cell pictures were taken at the membrane pores of the insert (cat. no. 83.3931.800, Sarstedt, Nümbrecht, Germany), as living cells need to be collected for growth for the downstream sequencing approach.

### Production of viruses from pooled genome-scale CRISPR-Cas9 libraries

The Human CRISPR Knockout Pooled Library (GeCKOv2) [11] (cat. no. 1000000048, Addgene, Watertown, USA) includes 65,383 gRNAs that target 19,050 genes, 1864 microRNAs and 1000 nonsense gRNAs. RT4-TP53DD cells were infected with a genome-wide CRISPR/Cas9 library and expanded according to the standard protocol [12]. For library infection, HEK293T cells were plated in 20 × 10 cm^2^ dishes at a density of 5 × 10^6^ cells per dish in 10 mL of DMEM supplemented with 10% FCS and 1% P/S for 24 h prior to transfection. For virus production, 3 µg of library plasmid, 1.8 µg of psPAX2 plasmid, and 0.3 µg of pMD2.G plasmids supplemented with 30 µL of PEI (Cat. 23,966, Polysciences, Warrington, USA) were mixed with 500 µL of DMEM without FCS on the day of transfection. On the second day, the medium was replaced with 10 mL of fresh DMEM supplemented with 10% FCS and 1% P/S per plate. On the third day post-transfection, the supernatant containing the virus was collected, and fresh medium was added to the cells. The collected virus was filtered twice through 0.45-µm filters and stored at 4 °C until the next day. The virus from the transfected cells was again collected on the fourth day and filtered twice, and all the viruses from the third and fourth days were then combined and stored at −80 °C until use. Considering that the backbone size of lentiCRISPRv2 is 14,873 bp, the molecular weight of lentiCRISPRv2 was calculated to be 9.18 × 10^6^ Da via web tools (https://www.bioinformatics.org/sms2/dna_mw.html). The number of copies was calculated as follows: plasmid DNA copy number = (plasmid amount [µg] × 6.022 × 10^23^) ÷ (9.18 × 10^6^ × 10^6^). Three micrograms of the library plasmid contained 1.968 × 10^11^ copies, which was approximately 3 × 10^6^-fold larger than the library size.

### Virus titration

Virus titration was performed according to Pan et al. [13] as follows. A total of 1 × 10^6^ RT4-TP53DD cells were plated per well of a six-well plate. The cells were infected with the lentivirus mixture:complete medium at ratios of 0:1, 1:1, 1:10, 1:100, 1:1000, and 1:10,000 (2 mL in total for each well) in the presence of 8 µg/mL polybrene overnight. The next day, RT4-TP53DD cells from each condition were reseeded equally into two six-well plates. Forty-eight hours post-infection, 2 µg/mL puromycin (cat. no. A1113803; Thermo Fisher Scientific, Waltham, USA) was added. After all the uninfected cells had been killed, the number of cells in each well was counted. The percentage of surviving cells for each viral concentration was calculated via the following formula: *P* (survival) = Number of cells with puromycin ÷ Number of cells without puromycin × 100%. The multiplicity of infection (MOI, *m*) was subsequently calculated via the following formula: *P* (survival) = 1–e^−*m*^. The MOI was calculated as 0.3 to ensure that most RT4-TP53DD cells were infected with only one virus. To ensure that all the sgRNAs covered the library cells, we set the coverage to 200 times the library size and calculated the number of cells by using the following formula: sgRNA library size × coverage ÷ MOI = starting cell number.

### Infection of RT4-TP53DD target cells for genome-scale CRISPR-Cas9 screening

With these calculations, at least 8.2 × 10^7^ RT4-TP53DD cells were needed. The target cells were plated in 15 six-well plates at a density of 1 × 10^6^ cells per well in 5 mL of complete RPMI 1640 (supplemented with 10% FBS and 1% P/S). Polybrene was added to the viral supernatant to a final concentration of 8 µg/mL. The cells were incubated at 37 °C overnight. The viral mixture was replaced with fresh complete RPMI 1640 medium the next day. Puromycin (2 µg/mL) was added to the medium 48 h post-infection. Puromycin selection was performed for 14 days, and the medium was changed every 2–3 days.

### RT4-TP53DD screen in EXTIM

In the first cycle of screening, after 14 days of puromycin selection of the RT4-TP53DD GeCKO v2 cells, library cells were seeded into 30 EXTIM setups at a cell density of 1 × 10^6^ in 30 µL of complete RPMI 1640 per EXTIM. Additionally, 1 × 10^6^ RT-TP53DD cells or RT-TP53DD cells with gGFP1 or gGFP2 were seeded into three EXTIM setups separately in parallel. The EXTIM apparatus was maintained in the incubator for 40 days, and the wells were changed every 2 days. After 40 days, the sandwich structures were removed, and invading cells in the Boyden chamber inserts and the bottom of the 12-well plate were transferred to 6-well plates by trypsinization and cultured. After expanding these cells for 2 weeks, the second cycle of screening was initialized to further enrich highly invasive cells. A total of 1 × 10^6^ cells per enrichment were suspended in 30 µL of complete medium and reseeded in one EXTIM setup, for a total of 11. Additionally, parental and gGFP#1 in the two EXTIM setups were set up in parallel and seeded at the same cell density. The invasive cells were collected 40 days after seeding via trypsinization to perform further steps.

### Identification of candidate gRNAs and genes

Genomic DNA extraction was performed via the QIAamp^®^ DNA Micro Kit (cat. no. 51306; Qiagen, Hilden, Germany) according to the standard protocol on the QIAGEN website (https://www.qiagen.com/). The collected genomic DNA was subjected to PCR amplification. The GoTaq^®^ G2 Flexi DNA polymerase system (cat. no. M7808, Promega Corporation, Madison, USA) was utilized for PCR amplification, which was carried out for 15 cycles to amplify the target gRNA sequences from the genomic DNA. A total of 400 ng of genomic DNA was added to the reaction mixture under the following conditions: 1 × (95 °C, 2 min), 30 × (95 °C, 30 s; 55 °C, 30 s; 72 °C 30 s) and 1 × (72 °C, 5 min). The PCR products were loaded for gel electrophoresis, after which the gel containing DNA products of the correct size was removed. DNA in the gel was extracted by using a QIAquick Gel Extraction Kit (cat. no. 28704, Qiagen, Hilden, Germany) following the protocol provided by the manufacturer. The purified DNA products were subcloned and inserted into the pCR™2.1 vector via the Invitrogen TA Cloning™ Kit (cat. no. K202020, Thermo Fisher, Waltham, USA) according to the manufacturer’s protocol. Single white clones (*n* = 168) growing on agar plates were picked and transferred into 15-mL Falcon tubes containing 5 mL of LB medium supplemented with 100 µg/mL ampicillin, which was then shaken at 220 rpm for 12‒16 h at 37 °C. The following day, plasmid DNA from each bacterial colony was isolated via the PureYield™ plasmid miniprep system (cat. no. A1223; Promega Corporation, Madison, USA). Plasmid DNA was sent for Sanger sequencing to determine the gRNA sequence. The candidate genes were identified by pairing with the library sequence provided by the Zhang laboratory. The primers used are listed in Supplementary Table ST5.

### Cells

HEK293T cells were obtained from the American Type Culture Collection (cat. no. CRL-3216) and were maintained in DMEM (cat. no. 116965092, Gibco-Thermo Fisher, Waltham, USA) supplemented with 10% fetal calf serum (FCS) and 1% penicillin/streptomycin (P/S). RT4 (cat. no. 91091914, ECACC, Porton Down, UK), T24 (cat. no. 85061107, ECACC, Porton Down, UK), and UMUC3 (cat. no. 96020936, ECACC, Porton Down, UK) cells were obtained from the European Collection of Authenticated Cell Cultures (ECACC) cultured in RPMI 1640 supplemented with 10% FCS and 1% P/S. Y235T cells were obtained from Prof. Jennifer Southgate (University of York, UK). HBLAK cells were generously provided by Dr. Michele Hoffmann (University of Düsseldorf, Düsseldorf, Germany) and can be purchased from CELLnTEC Advanced Cell Systems (cat. no. HBLAK, CELLnTEC Advanced Cell Systems, Bern, CH). Y235T and HBLAK cells were cultured in CnT-Prime medium (cat. no. CnT-PR, CELLnTEC, Bern, CH). BC61 (Cellosaurus, CVCL_D725) and BFTC-905 cell lines were provided by Prof. Wolfgang Schulz (University of Düsseldorf, Düsseldorf, Germany). BC61 cells were cultured in DMEM/F-12 medium supplemented with 10% FCS, 1% P/S, 1% insulin–transferrin–selenium (ITS), and 25 ng/ul basic fibroblast growth factor (bFGF). Simian virus 40 (SV40) large T antigen-immortalized UROtsa cells were received from Dr. Phillip Erben (University Hospital Mannheim, Mannheim, Germany). UROtsa cells were cultured in RPMI 1640 supplemented with 10% fetal calf serum FCS supplemented with 1% P/S, 1% GlutaMAX and 1% nonessential amino acids. BFTC-905 cells were cultured in DMEM supplemented with 20% FCS and 1% P/S. All the cell lines were maintained at 37 °C in a humidified incubator with 5% carbon dioxide.

### Transfection, infection, and generation of stable cell lines

DNA transfection, virus production, virus collection, and cell infection were performed according to standard procedures as described previously [14]. For the generation of cells with stable target gene expression, the cells were permanently selected with 2.0 µg/mL puromycin, 25.0 µg/mL hygromycin (cat. no. 10687010, Thermo Fisher Scientific, Waltham, USA), or 12.5 μg/mL blasticidin (cat. no. A1113903, Thermo Fisher Scientific, Waltham, USA).

### Antibodies

The following primary antibodies were utilized: anti-nuclei mouse antibody (1:100 for immunohistochemistry (IHC), ab254080, Abcam, Cambridge, UK); anti-HLA mouse antibody (1:100 for IHC, ab70328, Abcam, Cambridge, UK); anti-α-tubulin mouse antibody (1:2000 for WB T5168, Sigma-Aldrich, St. Louis, USA); anti-GAPDH mouse antibody (1:2000 for WB, MA5–15,738, Invitrogen, Thermo Fisher Scientific, Waltham, USA); anti-β-actin mouse antibody (1:10,000 for WB, A1978, Sigma-Aldrich, St. Louis, USA); anti-ORP3 mouse antibody (1:400 for IHC and 1:2000 for WB, sc398326, Santa Cruz, CA, USA); anti-GJB3 rabbit antibody (1:2000 for WB, ab236620, Abcam, Cambridge, UK); the truncated murine p53 protein TP53DD was detected with the mouse monoclonal PAb421 antibody (1:500 for WB, BML-SA293-0050, Enzo, NY, USA) and the full-length endogenous human TP53 was detected with the N-terminus specific (amino acids 11–25) monoclonal p53 antibody DO-1 (1:500 in WB, sc-126, Santa Cruz, CA, USA); DDR1 was detected with the anti-DDR1 mouse monoclonal antibody C-6 (1:500 for WB, sc-374618, Santa Cruz, CA, USA). The secondary antibodies used included horseradish peroxidase (HRP)-conjugated anti-mouse antibodies (1:5000 for WB, 7076S, Cell Signaling Technology, Danvers, USA) and horseradish peroxidase (HRP)-conjugated anti-rabbit antibodies (1:2000 for WB, 7074S, Cell Signaling Technology, Danvers, USA). One drop of universal immune-peroxidase anti-mouse (cat. no. A9044-2ML, Milliporesigma, Burlington, USA) or rabbit (cat. no. A9169-2ML, Milliporesigma, Burlington, USA) per piece of tissue was used for IHC.

### Plasmids

The TP53DD overexpression plasmid (pBABE-hygro-p53DD), which expresses a truncated murine p53 protein (amino acids 1–13 and 302–390 of murine p53) was obtained from Addgene (cat. no. 9058; Addgene, Watertown, USA). The human CRISPR knockout pooled library (GeCKOv2) [11] (cat. no. 1000000048, Addgene, Watertown, USA) and the control gRNAs were purchased from Addgene (cat. no. 60073 and 60,074; Addgene, Watertown, USA). DDR1 guide RNA (gRNA) was designed via an online tool (https://zlab.bio/guide-design-resources), and gRNAs were cloned and inserted into the pLentiCRISPRv2 [11] (cat. no. 52961, Addgene, Watertown, USA) according to the protocol from Zhang Lab (Feng Zheng, Broad Institute, Cambridge, USA). The sequences were confirmed by Sanger sequencing. The gRNA sequences are listed in Supplementary Table ST5. GJB3 and ORP3 overexpression plasmids as well as control vectors have been described recently [14, 15]. Further details are listed in the supplementary data (see Supplementary Table ST5 for the cloning primers). The DDR1 expression vector was purchased from DNASU (cat. no. HsCD00941305, DNASU, Tempe, USA).

### Ureteral sample collection and handling

Patient-derived ureter epithelial cells were prepared from the ureter epithelium of patients undergoing nephrectomy at the University of Ulm. Written consent was obtained from the patients. Sample treatment and RNA preparation were performed as described by Wang et al. [14].

### Total RNA, reverse transcription, and quantitative real-time PCR (RT‒qPCR)

Healthy human tissue samples across different organ types were purchased from Clontech (cat. no. 636643; Clontech Laboratories, California, USA). For the mRNA expression analysis, RNA extraction, reverse transcription and quantitative real-time PCR were carried out as previously described [14]. The sequences of primers used are provided in Supplementary Table ST5.

### Protein extraction and Western blot

Protein extraction and Western blot experiments were performed as described in Wang et al. [14]. Protein samples were loaded onto a 10% acrylamide gel, and subsequently, immunoblotting was conducted in accordance with established procedures [16].

### Ex vivo porcine bladder invasion model

The experiments were essentially conducted following the methodology outlined by Wezel et al. [10].

### Boyden chamber assays

Boyden chamber assays were conducted according to a previously established protocol [17]. For DDR1 inhibition, the cells were starved with serum-free RPMI 1640 containing 5 nM DDR1 inhibitor (VU6015929, Selleckchem, China) for 24 h, and the bottom wells were filled with 800 μL of RPMI 1640 containing 10% FCS, 1% P/S, and 5 nM DDR1 inhibitor.

### Hematoxylin and eosin (H&E) stainings and immunohistochemistry (IHC)

The tissue sections were cut into 4-µm sections. Hematoxylin and eosin (H&E) and IHC analyses of the tissues were carried out according to standard staining protocols as previously described [17].

### The tetrazolium-based colorimetric (MTT) assay

Cell viability was assessed via the MTT assay, which was conducted in a 96-well plate format, with 2000 cells in 100 μL of cell suspension per well. Each experimental condition was performed in six replicates. Measurements were performed at 24-h intervals up to 10 days after cell seeding. At each time point, 20 μL of MTT solution was added to each well, followed by a 4-h incubation at 37 °C. After incubation, the supernatant was carefully removed, preserving the formazan crystals formed. To solubilize the crystals, 100 μL of MTT lysis buffer was added to each well, and the plate was gently shaken in the dark for 10 min. The absorbance was recorded at 570 nm via a microplate spectrophotometer. Wells without cells were included as blank controls to establish baseline readings.

### Workflow of compound screening for anti-invasive cancer properties.

A total of 90 compounds from the LOPAC (Pfizer) library were screened for potential effects on cancer cell invasion. The compounds were first evaluated for their influence on T24 cell viability via the MTT assay. Of these, 28 compounds were excluded because of their cytotoxic effects, leaving 62 viable candidates with negligible effects on cell viability. These 62 compounds were then tested for their impact on T24 cell invasion and migration via a Boyden chamber assay, resulting in the exclusion of 59 compounds. The remaining three compounds were further evaluated for their effects on cancer cell invasion via EXTIM. Ultimately, only one compound demonstrated significant potential without compromising cell viability, making it a candidate for further investigation.

### Statistical analysis

Statistical analysis and graphical representations were carried out via GraphPad Prism 6 or Excel. Student’s *t*-test was used to compare differences between two groups. Unless otherwise noted, all values are presented as means with standard errors of the means (SEMs). The log-rank test was used to determine overall survival or recurrence-free survival in patients with bladder cancer. In all studies, *P* values < 0.05 were considered statistically significant.

## Results

### Limitations of evaluating cancer cell invasion via the Boyden chamber or ex vivo porcine bladder methods

**Fig. 1:**
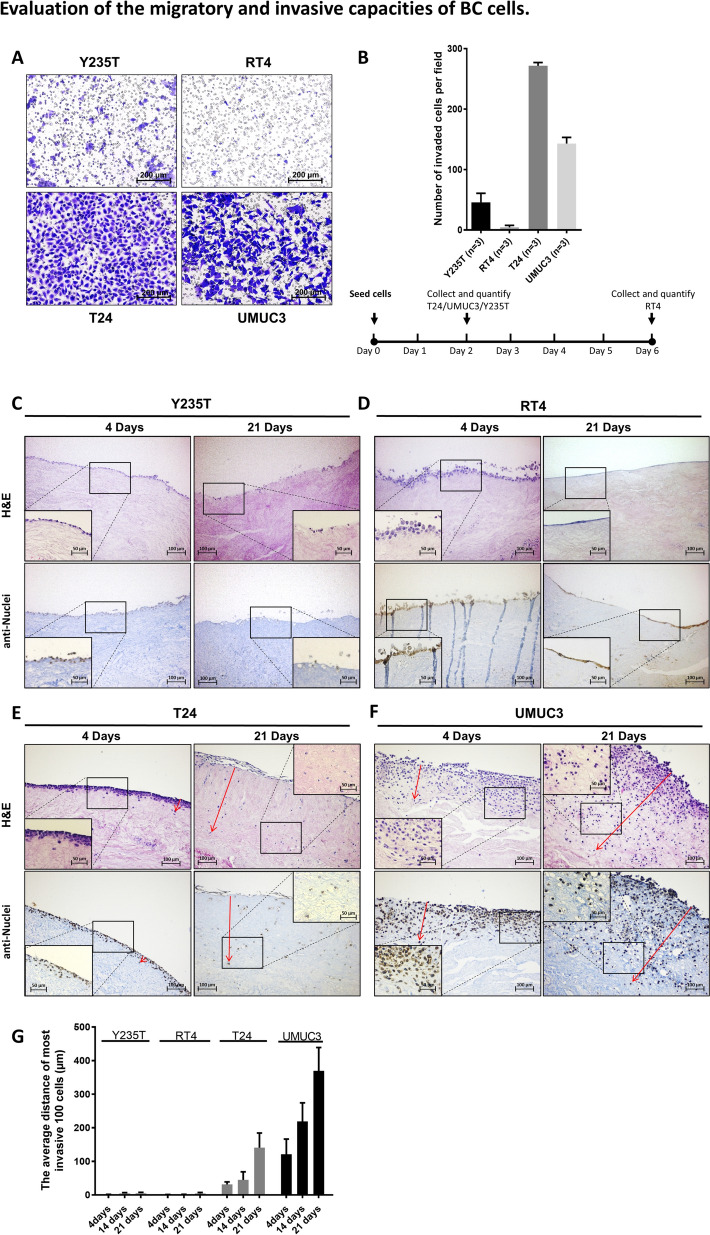
Evaluation of the migratory and invasive capacities of BC cells. **A** Representative images demonstrating the different invasive and migratory capacities of Y235T, RT4, T24, and UMUC3 cells in the Boyden chamber assay. Representative images of at least *n* = 3 independent experiments are shown. **B** (Top) Quantitation of the migratory and invasive capacities of Y235T, RT4, T24, and UMUC3 cells. The mean ± SEM values from *n* = 3 independent experiments are shown in the bar graphs. (Bottom) The chart illustrates the timeline of the experimental procedure. All the cells were seeded at the same time points on day 0. T24, UMUC3 and Y235T cells were evaluated on day 2 post-seeding, whereas RT4 cells were evaluated on day 6 post-seeding. **C**–**F** Representative images of hematoxylin/eosin (H&E) staining (top panels) and immunohistochemistry experiments using a human-specific anti-nuclei antibody (bottom panels) to visualize Y235T, RT4, T24, and UMUC3 cells in the porcine bladder ex vivo organ culture invasion model. To determine the different invasive capacities of the cells, tissues were collected at three different time-points post-seeding, on days 4, 14 and 21. For simplicity and space reasons, only pictures of tissues collected on days 4 and 21 are shown here. Insets: enlarged areas of the images shown by black boxes. All images captured at total magnification of 100× (**A**, **C** main panels, **D** main panels, **E** main panels and **F** main panels) or 400× (**C** insets, **D** insets, **E** insets and **F** insets). Scale bars: 200 μm (**A**), 100 μm (**C** main panels, **D** main panels, **E** main panels and **F** main panels) and 50 μm (**C** insets, **D** insets, **E** insets and **F** insets). Note that the size of nuclei seems larger at day 4 in Fig. 1D, likely owing to the condensed nuclei of RT4 cells at the 21 day time-point due to the differentiation of RT4 cells on porcine bladder, as previously described [10]. **G** Quantitation of the invasive capacities of Y235T, RT4, T24, and UMUC3 cells. The invasive distance of different cell lines was measured as previously described [15]. *n* = 6 (Y235T), *n* = 6 (RT4), *n* = 6 (T24), and *n* = 6 (UMUC3) independent experiments were performed. The mean ± SEM values are shown in the bar graph

To emphasize the advantages and limitations of Boyden chamber technique in the context of BC, we compared the invasive and migratory capacities of the invasive BC cell lines T24 and UMUC3, the noninvasive BC cell line RT4 and the ureter-derived, telomerase-immortalized but nontransformed (i.e., nontumor) Y235T cell strain, which has a normal karyotype. As expected, T24 and UMUC3 cells readily passed through the Boyden chamber within 48 h, whereas only a few RT4 cells were detected on the lower side of the Boyden chamber after 6 days (RT4: 4.5 cells/field; Figs. 1A, B). Interestingly, at the 48-h timepoint, we observed significant migration of Y235T cells (Y235T: 45.8 cells/field; Fig. 1B), although with much lower efficiency than the highly invasive BC cell lines T24 and UMUC3 (T24: 271.6 cells/field; UMUC3: 143.0 cells/field; Fig. 1B).

To better model the tissue context, we employed an ex vivo porcine bladder model [10, 14, 15, 17]. In this model, neither Y235T nor RT4 cells invaded porcine bladder tissue but remained on the surface of the de-epithelized porcine bladder throughout the observation period of up to 21 days (Fig. 1C, D). In contrast, T24 and UMUC3 cells were detected in the porcine stroma at the 4-day time point and in the muscle layer at the 21-day time point (Fig. 1E–G). A time-dependent increase of invasion of T24 and UMUC3 cells in the stroma/muscle was observed and quantified (Fig. 1G), following our established quantification procedure [14].

### Establishment of an ex vivo bladder cancer invasion model: the EXTIM

**Fig. 2:**
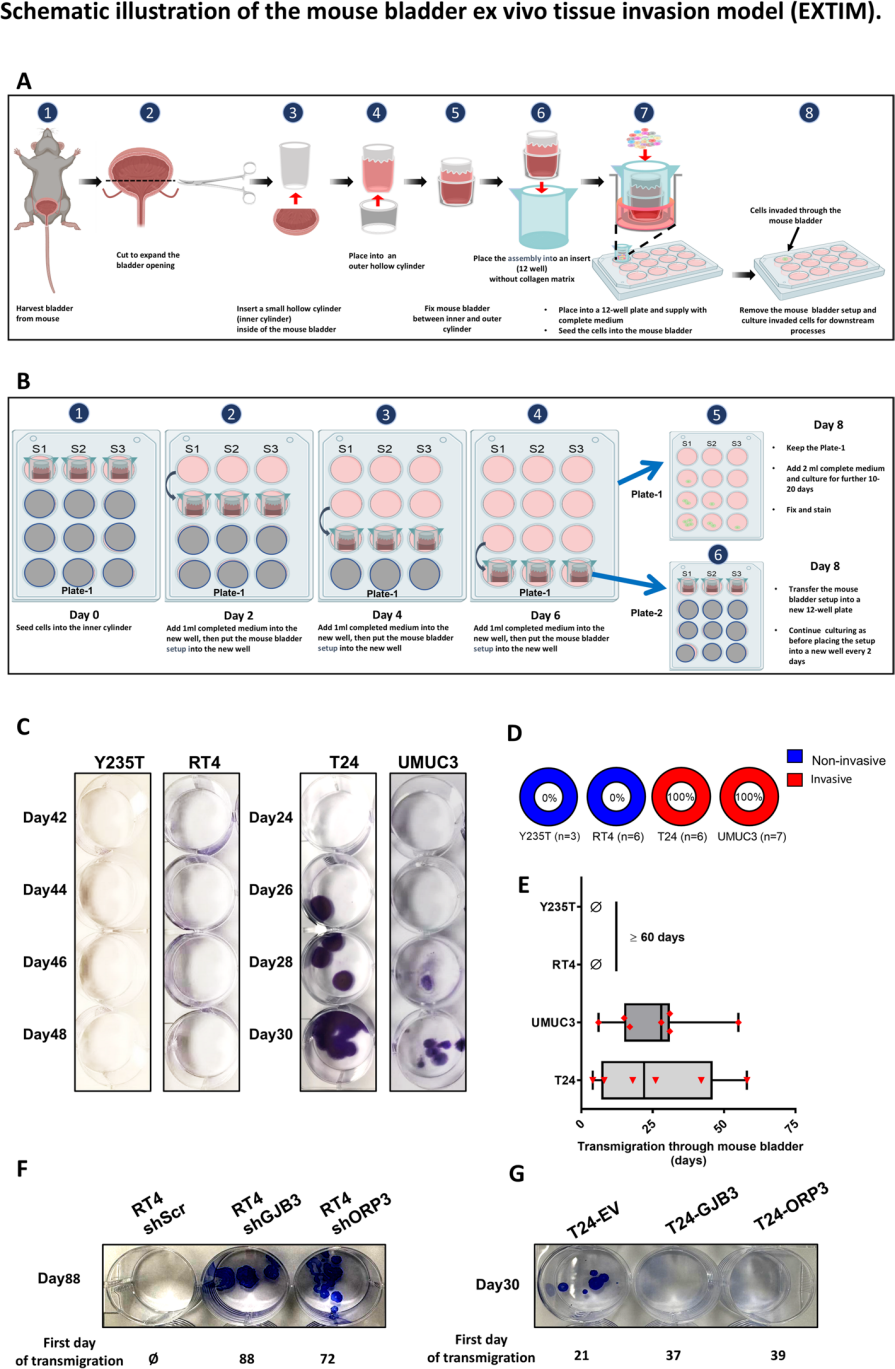
Schematic illustration of the mouse bladder ex vivo tissue invasion model (EXTIM).** A** The flowchart illustrates the EXTIM establishment procedure. See the main text and the “Materials and methods” section for the detailed experimental procedure. **B** The flow chart illustrates the procedure for long-term maintenance and subculturing of the EXTIM setup to monitor the transmigratory capacity of cells through the mouse bladder. See the main text and the “Materials and methods” section for the detailed experimental procedure. **C** The representative images illustrate the different invasive/transmigratory capacities of Y235T, RT4, T24, and UMUC3 cells in the EXTIM. T24 and UMUC3 cells transmigrated through mouse bladder tissue and proliferated to form colonies in the culture plates. These cells were fixed and stained in the wells for visualization (see “Materials and methods” section). Notably, an increasing number of cells per time point were observed during culture in consecutive wells. The exemplary images depict the staining of colonies in the plate formed from cells that transmigrated through the mouse bladder. *n* = 3 (Y235T), *n* = 6 (RT4), *n* = 6 (T24), and *n* = 7 (UMUC3) distinct experiments were performed. Note that, for each cell line, only one of the repeat experiments is shown (depicted here are example plates of the repeat experiments with the plates showing no colony on day 48 post-seeding for Y235T and RT4 and example plates of the repeat experiments with the plates showing colonies on day 26 post-seeding for T24 and on day 28 post-seeding for UMUC3). **D** Pie charts showing the transmigation frequency of different cell lines through the mouse bladder. While T24 and UMUC3 cells transmigrated through the mouse bladder and formed colonies in the wells in all the repeated experiments, Y235T cells and RT4 cells never transmigrated through the mouse bladder. The values in the center of each pie represent the percentage of invasive events. The *n* numbers are indicated in parentheses: Y235T (*n* = 3), RT4 (*n* = 6), T24 (*n* = 6), and UMUC3 (*n* = 7) independent experiments were performed. Experiments with invasive events are indicated by red pie charts, and those with noninvasive events are indicated by blue pie charts. **E** The bar graph illustrates the first day, on which cells were visible after transmigration through the mouse bladder during the observation time in independent repeat experiments; i.e., for T24 cells, the cells were first observed transmigrating through the mouse bladder and proliferating to form colonies on day 4 post-seeding in experiment 1 and on day 26 in experiment 3. The results of repeat experiment 3 are shown in **C**. ∅: No transmigration during the observation time up to 60 days. Filled downward triangle or filled diamond: Each sign represents the corresponding first day, on which T24 or UMUC3 cells were visible in the culture plate after transmigration through the mouse bladder in the respective independent repeat experiment. **F** The representative images compare the transmigration capacities of RT4-shScr, RT4-shGJB3, and RT4-shORP3 EXTIM cells on day 88 post-seeding. The representative images depict the staining of cell colonies in the culture plate that formed from cells that had transmigrated through the mouse bladder. The days below the images indicate the first day on which cells were visible after transmigration through the mouse bladder during the observation time. ∅: no transmigration during the observation time. **G** Representative images comparing the transmigration capacities of T24-EV, T24-GJB3, and T24-ORP3 EXTIM cells on day 30 post-seeding. The exemplary images depict the staining of colonies in the plate formed from cells that transmigrated through the mouse bladder. The days below the images indicate the first day on which cells were visible after transmigration through the mouse bladder during the observation time

**Fig. 3:**
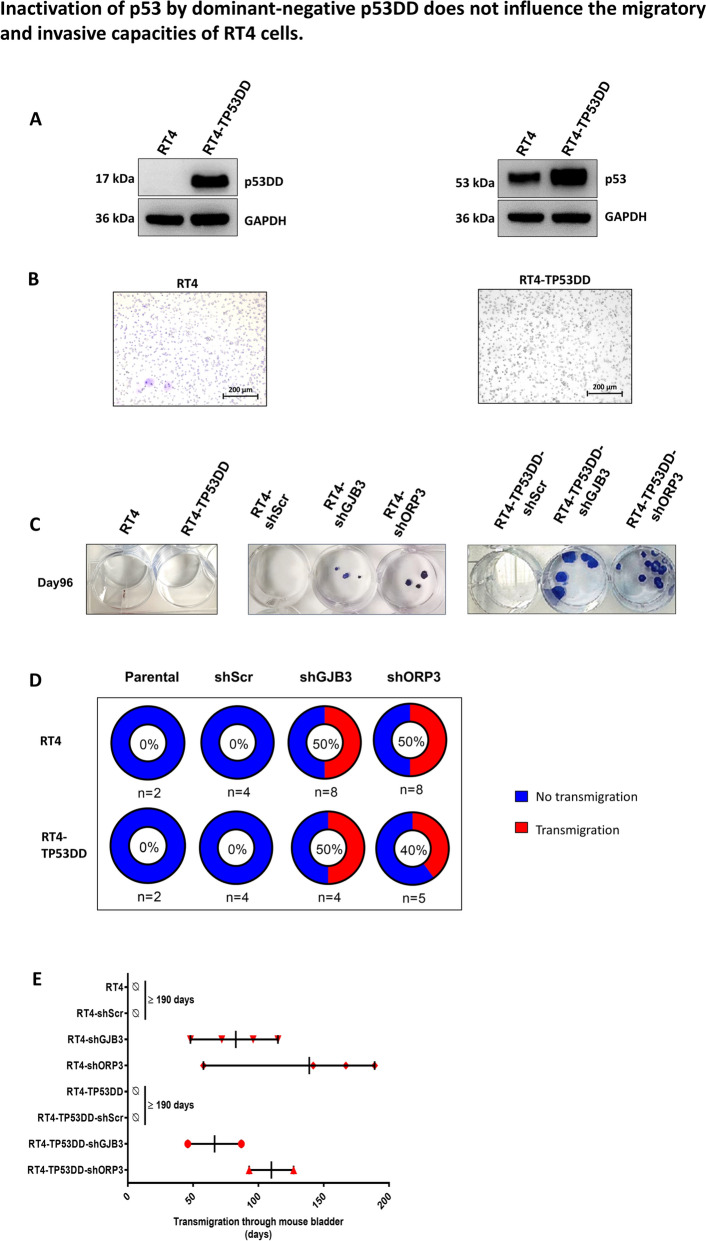
Inactivation of p53 by dominant-negative p53DD does not influence the migratory and invasive capacities of RT4 cells.** A **(Left) Representative Western blot showing p53DD protein levels in RT4 and RT4-TP53DD cells. *n* = 3 independent experiments were performed. Note that the mouse monoclonal PAb421 antibody was used to detect the truncated protein p53DD. Although PAb421 is suitable to detect the full-length p53 protein of human, rat, and mouse origin, the endogenous full-length human p53 is not visible in the uncropped full blot (see Supplementary Figure showing uncropped full blots of all immunoblots) owing to strong p53DD overexpression in RT4 cells. (Right) Representative Western blot results indicating p53 protein levels in RT4 and RT4-TP53DD cells. GAPDH protein levels were used as the loading control. *n* = 3 independent experiments were performed. To determine the expression levels of endogenous full-length human p53 protein in RT4 cells, we used the highly sensitive DO-1 antibody. This antibody is directed against the N-terminal amino acids of human p53 (aa 11–25 of human p53), which has no similarity and conservation in the murine TP53DD protein, which was used in this study (pBABE-hygro-TP53DD). This dominant-negative p53 protein is a truncated variant of the murine p53 protein, and lacks amino acids 14–301 and thus contains the first 13 codons followed by codons 302–390 of the murine p53 protein. Although the DO-1 (sc-126) antibody can be used to detect full-length TP53 of human, rat, and mouse origin, it does not detect the truncated TP53DD protein of mouse origin. It is also noteworthy that we repeatedly observed slightly higher full-length p53 levels in RT4 cells expressing the truncated p53 protein (RT4-TP53DD) compared with parental RT4 cells. This is likely due to the stabilization of the endogenous p53 protein levels by the ectopic dominant negative TP53DD protein. Boyden chamber assays revealed similar invasive and migratory capacities of RT4 and RT4-TP53DD cells. The representative images show the results of the Boyden chamber approach, stained at 144 h post-seeding. No transmigratory RT4 or RT4-TP53DD cells were observed. Representative images of *n* = 3 independent experiments are shown. **C** Representative images showing the transmigration capacities of RT4 and RT4-TP53DD EXTIM cells. (Left) RT4 and RT4-TP53DD cells similarly do not transmigrate through the mouse bladder in the EXTIM. *n* = 2 (RT4) and *n* = 2 (RT4-TP53DD) distinct experiments were performed. The exemplary images depict no cell transmigration through the mouse bladder on day 96 post-seeding. (Middle) Representative images comparing the transmigratory capacities of RT4-shScr, RT4-shGJB3 and RT4-shORP3 EXTIM cells on day 96 post-seeding. *n* = 4 (RT4-shScr), *n* = 8 (RT4-shGJB3) and *n* = 8 (RT4-shORP3) distinct experiments were performed. (Right) Representative images comparing the transmigratory capacities of RT4-TP53DD-shScr, RT4-TP53DD-shGJB3, and RT4-TP53DD-shORP3 EXTIM cells on day 96 post-seeding. The exemplary images depict the staining of colonies in the plate formed from cells that transmigrated through the mouse bladder. *n* = 4 (RT4-TP53DD-shScr), *n* = 4 (RT4-TP53DD-shGJB3), and *n* = 5 (RT4-TP53DD-shORP3) distinct experiments were performed. **D** Pie charts illustrating the results, as described in **C**. The values in the center of each pie represent the percentage of transmigration events. The *n* numbers are indicated in parentheses: RT4 (*n* = 2), RT4-shScr (*n* = 4), RT4-shGJB3 (*n* = 8), RT4-shORP3 (*n* = 8), RT4-TP53DD (*n* = 2), RT4-TP53DD-shScr (*n* = 4), RT4-TP53DD-shGJB3 (*n* = 4), and RT4-TP53DD-shORP3 (*n* = 5) independent experiments were performed. Experiments with transmigration events are indicated by red pie charts, and non-transmigration events are indicated by blue pie charts. **E** The bar graph shows the first day on which transmigration of the indicated cells through the mouse bladder was detected during the observation time in independent experiments. For RT4-shGJB3 cells, the cells were first observed transmigrating through the mouse bladder and proliferating to form colonies on days 48, 72, 96, and 115 post-seeding in four repeat experiments, and for RT4-shORP3 cells, the cells were first observed transmigrating through the mouse bladder and proliferating to form colonies on days 58, 142, 167, and 189 post-seeding in four repeat experiments. ∅: No transmigration during the observation time up to 190 days. Filled downward triangle: The first day of cell transmigration through the mouse bladder during the observation of RT4-shGJB3 cells in EXTIMs in independent repeat experiments. Filled diamond: The first day of cell transmigration through the mouse bladder during the observation of RT4-shORP3 cells in EXTIMs in independent repeat experiments. Filled circle: The first day of cell transmigration through the mouse bladder during the observation of RT4-TP53DD-shGJB3 EXTIM cells in independent experiments. Filled upward triangle: The first day of cell transmigration through the mouse bladder during the observation of RT4-TP53DD-shORP3 EXTIM cells in independent experiments. The results are shown as scatter dot blot with range, indicating the mean value. Scale bars: 200 μm (**B**). Images captured at total magnification of 100× (**B**)

**Fig. 4:**
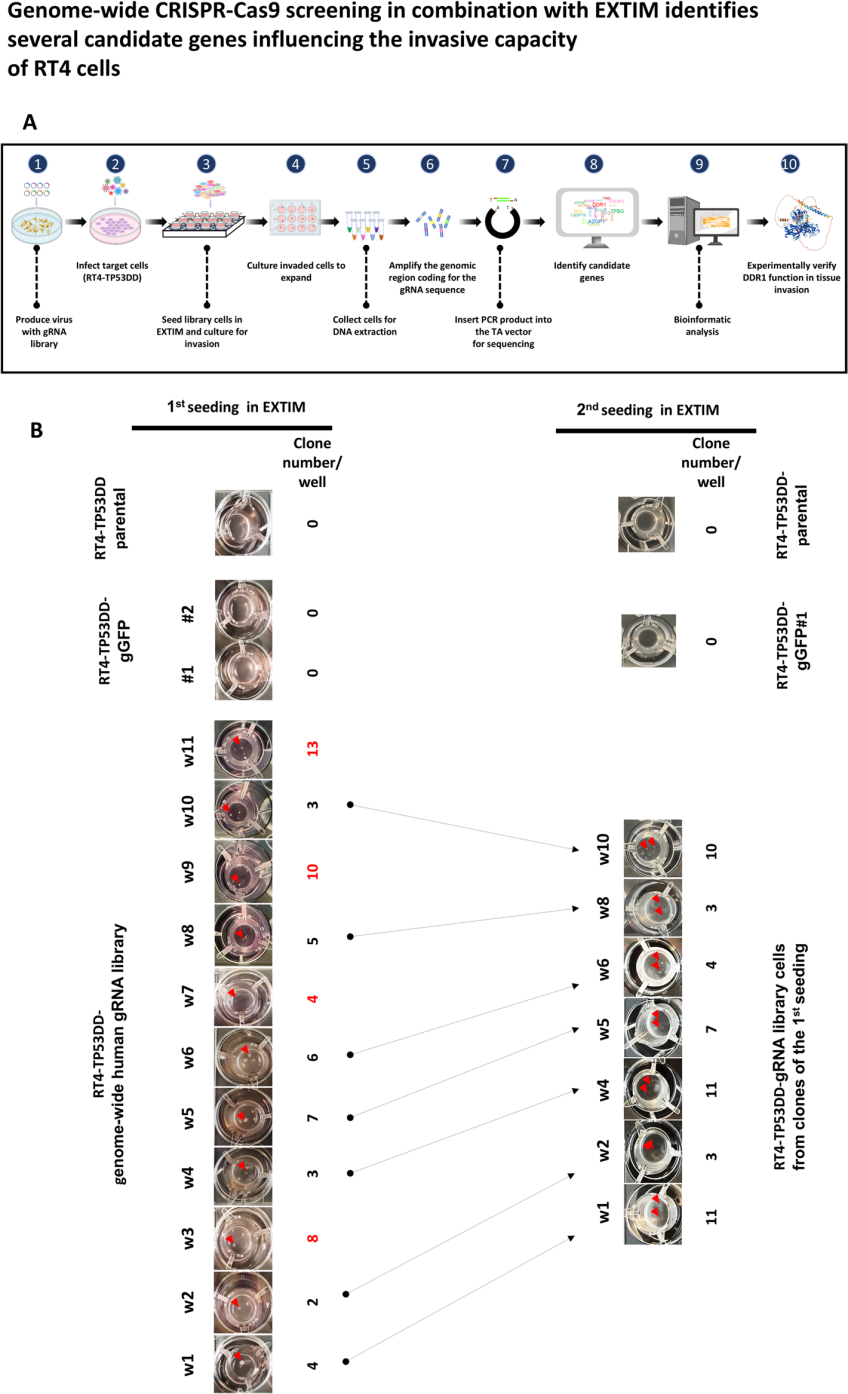
Genomewide CRISPR-Cas9 screening in combination with EXTIM identifies several candidate genes influencing the invasive capacity of RT4 cells.** A** The flowchart shows the procedure of CRISPR library screening via EXTIM. **B** Representative images of the cells after first (left) and second (right) seeding of RT4-TP53DD cells with gGFP or the genomewide human gRNA library in EXTIM. The pictures show the insert with the cell colonies in the culture well after removal of the EXTIM assembly. (Left) The pictures show cell colonies on the inserts that transmigrated through the mouse bladder (growing cell colonies were observed in 11 out of 30 EXTIM setups). As controls, RT4-TP53DD cells and RT4-TP53DD cells with gGFP#1 or gGFP#2 were seeded into three EXTIM setups in parallel. (Right) For the second seeding, the cells from the first seeding were collected, expanded, and reseeded. The pictures show cell colonies on the inserts that transmigrated through the mouse bladder (growing cell colonies were observed in 7 out of 11 EXTIM setups). As controls, RT4-TP53DD cells and RT4-TP53DD cells with gGFP#1 were seeded into two EXTIM setups in parallel. The arrows indicate that the cells seeded via the 2nd EXTIM approach were expanded from the corresponding inserts of the first EXTIM approach. The EXTIM setups indicated with red numbers did not give rise to colonies in the second seeding. The identified colony numbers in different wells are marked on the right side of the images

**Fig. 5:**
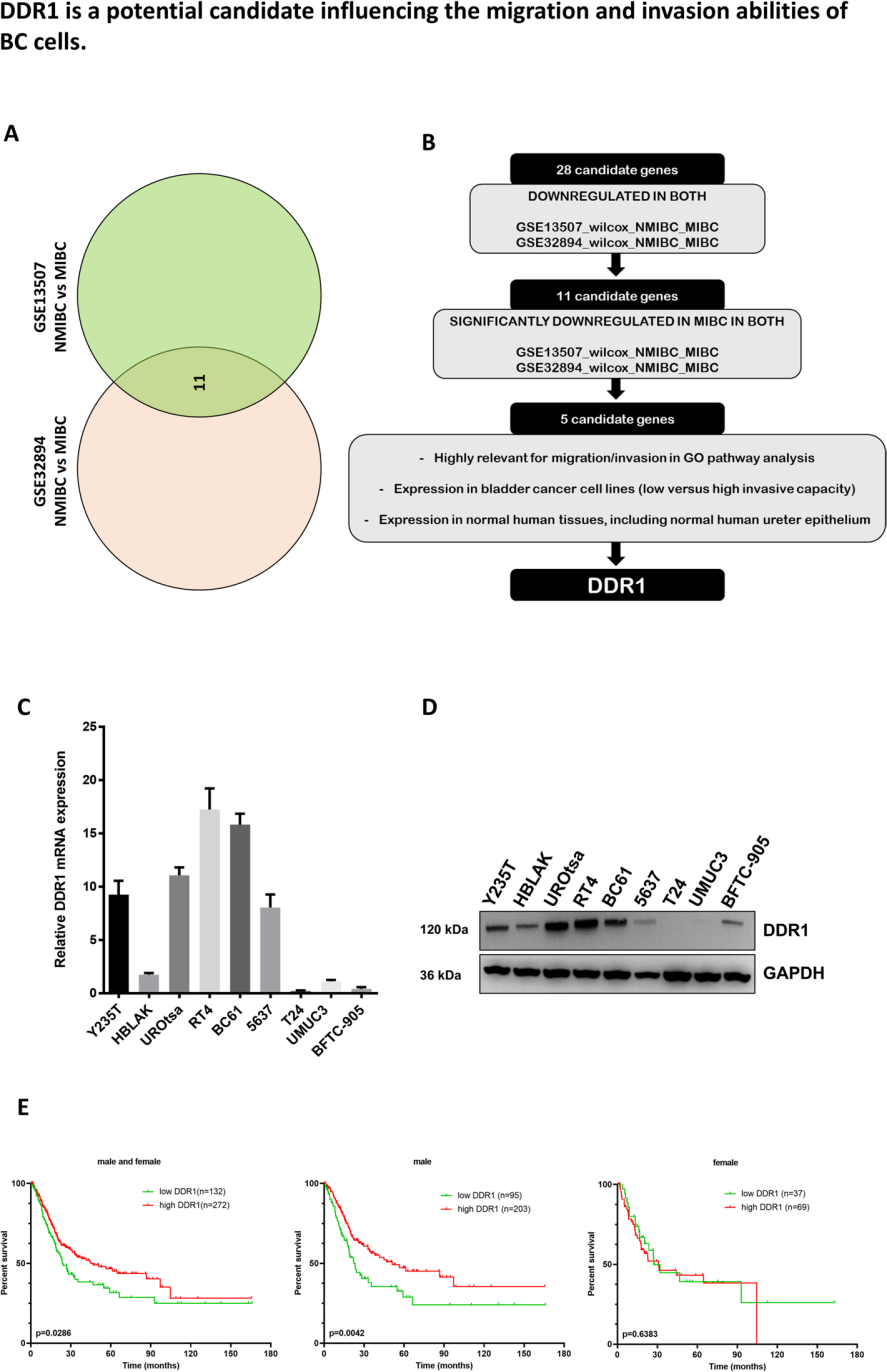
DDR1 is a potential candidate influencing the migration and invasion abilities of BC cells.** A** Venn diagram showing that 11 out of 28 genes were commonly downregulated in MIBC compared to NMIBC, both in the CHUN (GSE13507) cohort (NMIBC: *n* = 103 and MIBC: *n* = 62) and the LUND (GSE32894) cohort (NMIBC: *n* = 215 and MIBC: *n* = 93). The Venn diagrams are based on the Wilcoxon test with a *p* value threshold of 0.05. The microarray datasets GSE13507 and GSE32894 were obtained from the Gene Expression Omnibus (GEO) database (https://www.ncbi.nlm.nih.gov/). GSE13507 contained NMIBC (*n* = 103) and MIBC (*n* = 62). GSE32894 contained NMIBC (*n* = 213) and MIBC (*n* = 93). Significance was assessed using the unpaired *t* test. A *P*-value < 0.05 was considered statistically significant. **B** Selection procedure for the top candidate genes. Of the 28 protein-coding genes identified, 11 (*DDR1*, *AZGP1*, *STK19*, *STEAP3*, *LAT*, *IL11*, *FERMT1*, *TMEM18*, *EMP2*, *PRRT3*, and *BICD1*) were found to be downregulated in MIBC compared with NMIBC in at least one dataset. However, only five (*DDR1*, *AZGP1*, *STK19*, *STEAP3*, and *LAT*) were significantly downregulated in MIBC relative to NMIBC in both datasets. Subsequent Gene Ontology (Supplementary Fig. ST3) analysis of the relative mRNA levels of the five genes in noninvasive and invasive BC cell lines (Supplementary Fig. SF4A) as well as in a set of apparently healthy human tissues and human ureter-derived epithelial cells (Supplementary Fig. SF4B) revealed *DDR1* as the top candidate gene. **C** The bar graph illustrates the relative *DDR1* mRNA expression in ureter-derived cell strains (Y235T, HBLAK, and UROtsa) and a set of BC cell lines. The mRNA levels were normalized to those of *GAPDH*. *n* = 3 independent experiments were performed. The error bars represent the means ± SEMs. **D** The Western blot results illustrate the relative DDR1 protein expression levels in ureter-derived cell strains (Y235T, HBLAK, and UROtsa) and a set of BC cell lines. GAPDH was used as the loading control. *n* = 2 independent experiments were performed. **E** Kaplan‒Meier survival curves showing overall survival (OS) for patients with BC in correlation with *DDR1* mRNA levels, stratified by expression level (high versus low). The source data [33] were downloaded from the cBioPortal [34, 35], and OS was calculated via PRISM software with a cut-off of 6100 tpm. The *p* value was calculated via the log-rank test. Left panel: All patients with BC (high: *n* = 132, low: *n* = 272). High *DDR1* mRNA expression was associated with improved survival (*P* = 0.0286). Middle panel: Male patients with BC (High: *n* = 95, Low: *n* = 203). Male patients with high *DDR1* mRNA expression had significantly better OS than did those with low expression (*P* = 0.0042). Right panel: Female patients with BC (high: *n* = 37, low: *n* = 69). There was no statistically significant difference in OS between the high and low *DDR1* mRNA expression groups (*P* = 0.6383). Note: OS is plotted against time in months

**Fig. 6:**
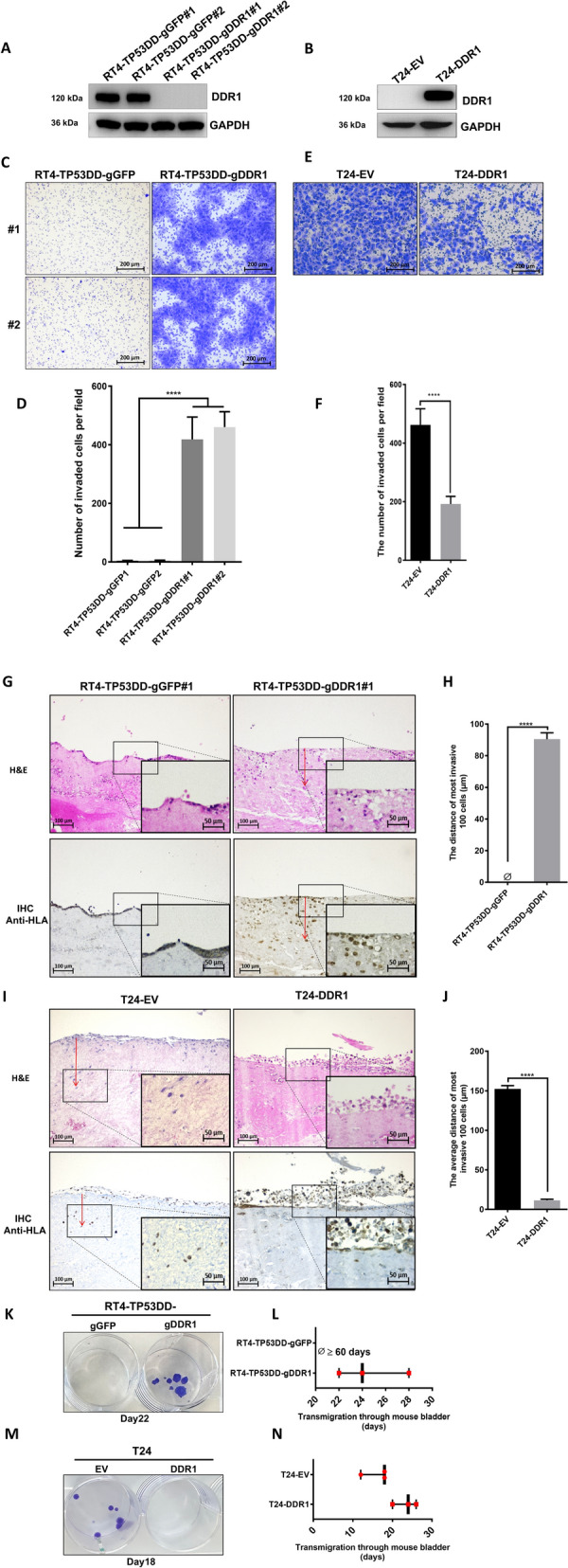
DDR1 regulates the invasive and migratory capacities of bladder cancer cells via the Boyden chamber approach.** A** Representative Western blot results depict DDR1 protein levels in RT4-TP53DD cells expressing guide RNAs that target green fluorescence protein (GFP) as a negative control (gGFP#1 and gGFP#2 represent two different gRNAs directed against GFP) or expressing guide RNAs that target DDR1 (gDDR1#1 and gDDR1#2 represent two different gRNAs directed against DDR1). GAPDH served as a loading control. *n* = 2 independent experiments were conducted. The representative Western results depict DDR1 protein levels in T24 cells with an empty vector (T24-EV) or T24 cells with ectopic DDR1 expression. GAPDH was used as a loading control. *n* = 3 independent experiments were performed. **C**, **D** The invasive and migratory capacities of RT4-TP53DD cells with DDR1- and GFP-targeting gRNAs were detected with a Boyden chamber. The representative images depict cell invasion through the Boyden chamber on day 6 post-seeding. **D** Quantitation of the invasive and migratory capacities of RT4-TP53DD cells with DDR1- or GFP-targeting gRNAs in a Boyden chamber. *n* = 3 independent experiments were performed. The bar graphs display the mean ± SEM values, and a two-tailed Student’s *t* test was used to assess significance. ****P < 0.0001. **E**, **F** The invasive and migratory capacities of T24 cells with ectopic DDR1 expression. The representative images depict cell transmigration through the Boyden chamber on day 2 post-seeding. **F** Quantitation of the invasive and migratory capacities of T24 cells overexpressing ectopic DDR1 via a Boyden chamber assay. *n* = 3 independent experiments were performed. The bar graphs display the mean ± SEM values, and a two-tailed Student’s *t* test was used to assess significance. **** *P* < 0.0001. **G** The representative pictures show the results of hematoxylin/eosin (H&E) staining (top panels) and immunohistochemistry experiments using a human-specific anti-HLA antibody (bottom panels) to visualize the invasive capacities of RT4-TP53DD cells with DDR1- and GFP-targeting gRNAs in the porcine bladder ex vivo organ culture invasion model. Tissues were collected on day 21 post-seeding. Insets: enlarged areas of the images are shown by black boxes. The invasion distance of an exemplary cell is shown by a red arrow. **H** Quantitation of the invasive capacities of RT4-TP53DD cells with DDR1- and GFP-targeting gRNAs in a porcine bladder ex vivo organ culture model.* n* = 3 independent experiments were performed. The means ± SEMs are shown in the bar graph, and significance was determined via two-tailed Student’s *t* test. ****P < 0.0001. **I** The representative images show the results of hematoxylin/eosin (H&E) staining (top panels) and immunohistochemistry experiments using a human-specific anti-HLA antibody (bottom panels) to visualize the invasive capacities of T24 cells with ectopic DDR1 in the porcine bladder ex vivo organ culture invasion model. Tissues were collected on day 14 post-seeding. Insets: enlarged areas of the images are shown by black boxes. The invasion distance of one cell is shown by a red arrow. **J** Quantitation of the invasive capacities of T24 cells with ectopic DDR1 expression in a porcine bladder ex vivo organ culture model.* n* = 3 independent experiments were performed. The means ± SEMs are shown in the bar graph, and significance was determined via two-tailed Student’s *t* test. ****P < 0.0001. **K** The representative images illustrate the transmigration capacities of RT4-TP53DD-gGFP and RT4-TP53DD-gDDR1 EXTIM cells on day 22 post-seeding. The exemplary images depict the staining of colonies in the culture plate formed from cells that transmigrated through the mouse bladder. Three (RT4-TP53DD-gGFP) and three (RT4-TP53DD-gDDR1) distinct experiments were performed. Note that, for each cell line, only one of the repeat experiments is shown (depicted here are example plates of the repeat experiments showing no colony formation (i.e., no transmigration) of RT4-TP53DD-gGFP cells until day 60 post-seeding, whereas colonies were already detectable on day 22 post-seeding with RT4-TP53DD-gDDR1 cells, indicating a gain of transmigration ability upon loss of DDR1 expression. **L** The graph illustrates the first day at which the first transmigratory cells with the indicated genotype were observed in the culture plates. For RT4-TP53DD-gDDR1 cells, growing colonies were observed on days 22, 24, and 28 in three different experiments. ∅: No transmigration. Filled square: the first day, at which point the first cells were observed in the culture plates. **M** The representative pictures illustrate the transmigratory capacities of T24-EV and T24-DDR1 cells in EXTIMs on day 18 post-seeding. The exemplary images depict the staining of colonies in the plate formed from cells that transmigrated through the mouse bladder. Three (T24-EV) and three (T24-DDR1) distinct experiments were performed. Note that, for each cell, only one of the repeat experiments is shown (depicted here are example plates of the repeat experiments showing no colonies of T24-DDR1 cells until day 26 postseeding, whereas colonies of T24-EV cells from day 18 post-seeding are shown). **N** The graph illustrates the time points at which the first transmigratory cells with the indicated genotype were identified in the culture plates. For T24-EV cells, growing cell colonies were observed on days 12 and 18 (2 ×), whereas growing T24-DDR1 cells were observed on days 20, 24, and 26. Filled circle and filled square: the first day at which the cells were first observed in the culture plates. Scale bars: 200 μm (**C** and **E**), 100 μm (**G** main panels and **I** main panels) and 50 μm (**G** insets and **I** insets). Images captured at total magnification of 100× (**C** and **E**, **G** main panels and **I** main panels) or 400× (**G** insets and **I** insets)

**Fig. 7:**
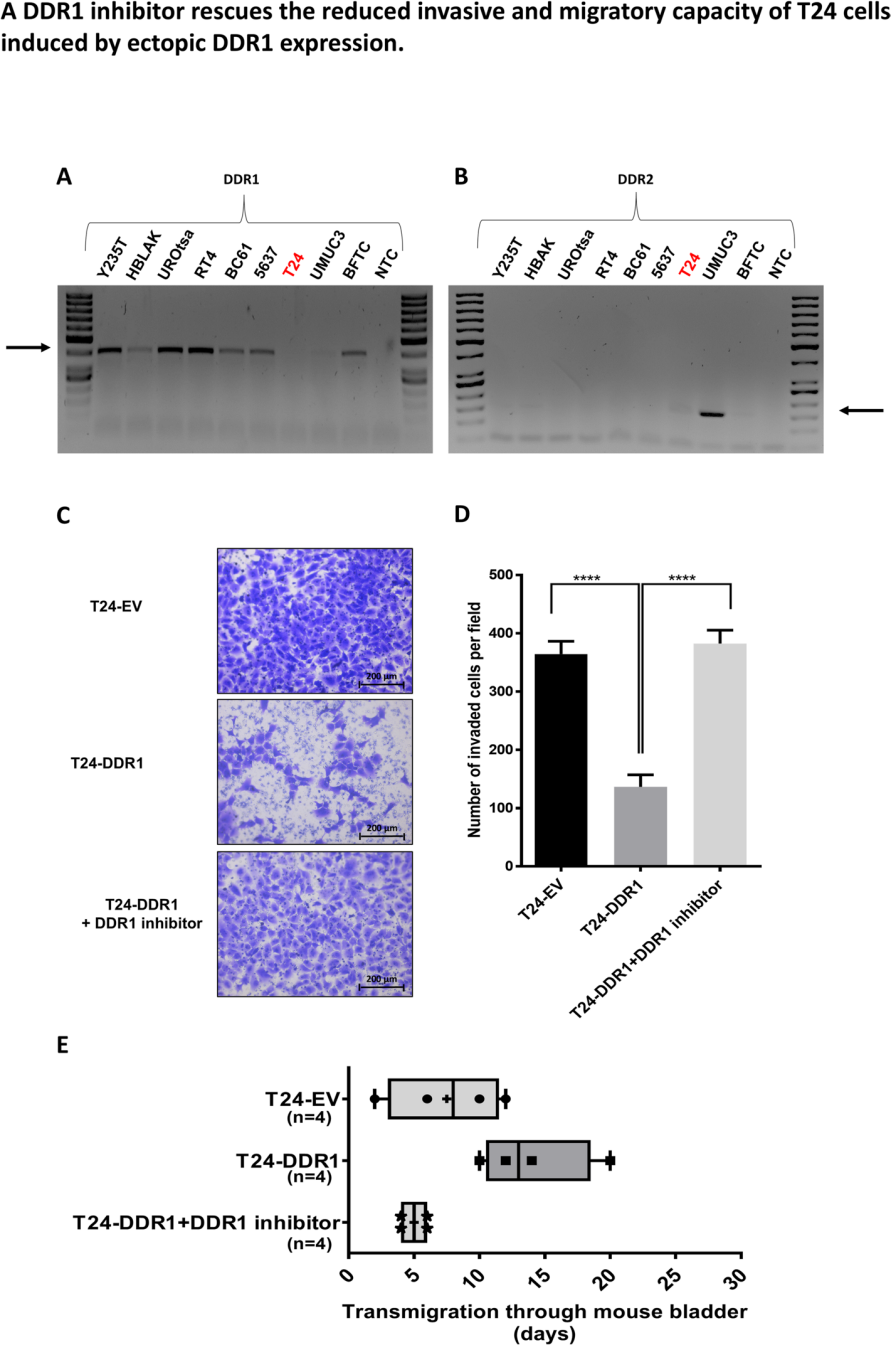
A DDR1 inhibitor rescues the reduced invasive and migratory capacity of T24 cells induced by ectopic DDR1 expression.** A**, **B** Semiquantitative PCR analysis for the detection of DDR1 and DDR2 mRNA expression. The black arrows indicate DDR1 (398 bp) (**A**) or DDR2 (157 bp) (**B**) PCR products in bladder cell lines. T24 cell line is highlighted in red. **C.** The invasive and migratory capacities of T24 cells with ectopic DDR1 expression in the presence or absence of a selective DDR1 inhibitor (treated with 5 nM VU6015929, cat. no. S6817, Selleckchem, Texas, USA) were detected in a Boyden chamber. The representative images depict cell invasion through the Boyden chamber on day 2 post-seeding. **D** Quantitation of the invasive and migratory capacities of T24 cells overexpressing ectopic DDR1 with or without DDR1 inhibitor treatment in a Boyden chamber. *n* = 3 independent experiments were performed. The bar graphs display the mean ± SEM values, and a two-tailed Student’s *t* test was used to assess significance. ****P < 0.0001. **E** The bar graph illustrates the first day on which transmigration of the indicated cells through the mouse bladder was detected during the observation time in independent repeat EXTIM experiments. T24-EV cells were first observed to transmigrate through the mouse bladder and to proliferate to form colonies on days 2, 6, 10, and 12. For T24-DDR1 cells, the cells were first observed transmigrating through the mouse bladder and proliferating to form colonies on days 10, 12, 14, and 20. For T24-DDR1 + DDR1 inhibitor, the cells were first observed transmigrating through the mouse bladder and proliferating to form colonies on days 4, 4, 6, and 6 post-seeding, respectively, in four repeated experiments. Filled circle: The first day of cell transmigration through the mouse bladder during the observation of T24-EV-treated cells in independent repeat EXTIM experiments. Filled square: The first day that cells transmigrated through the mouse bladder during the observation of T24-DDR1 cells in independent repeat EXTIM experiments. Filled start: The first day of cell transmigration through the mouse bladder during the observation of T24-DDR1 + DDR1 inhibitor EXTIM cells in independent experiments. Images captured at total magnification of 100× (**C**). Scale bars: 200 μm (**C**)

**Fig. 8:**
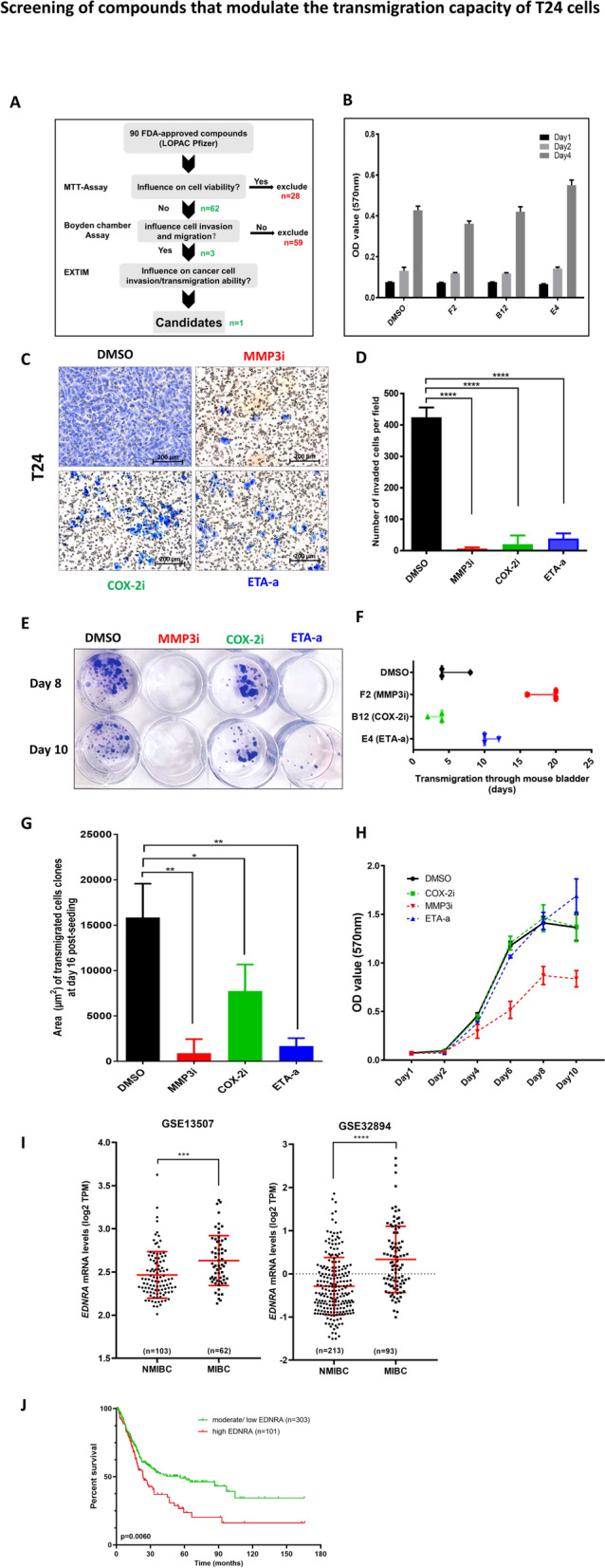
Screening of compounds that modulate the transmigration capacity of T24 cells.** A **The flowchart illustrates the screening of candidate compounds. See the main text and the “Materials and methods” section for the detailed experimental procedure. **B** Evaluation of the toxicity of the indicated compounds (MMP3 inhibitor: F2, MMP3i; COX-2 inhibitor: B12, COX-2i; and endothelin receptor antagonist: E4, ETA-a) on T24 cells as determined by the MTT assay at days 1, 2, and 4 post-seeding. The bar graphs show the relative number of living cells treated with the indicated compounds (see “Materials and methods” for calculations). The bar graphs display the mean ± SEM values, and a two-tailed Student’s *t* test was used to assess significance. ****P < 0.0001. MTT assays were performed in sextuplicate. **C** Impact of the MMP3i, COX-2i, and ETA-a compounds on the invasive and migratory capacities of T24 cells as determined by the Boyden chamber assay. The representative images depict cell invasion through the Boyden chamber on day 2 post-seeding. Images captured at total magnification of 100× (**C**). Scale bars: 200 μm (**C**). **D** Quantitation of the impact of the MMP3i, COX-2i and ETA-a compounds on the invasive and migratory capacities of T24 cells as determined by the Boyden chamber assay. *n* = 3 independent experiments were performed. The bar graphs display the mean ± SEM values, and a two-tailed Student’s *t* test was used to assess significance. **** *P* < 0.0001. **E** Impact of the MMP3i, COX-2i, and ETA-a compounds on the transmigratory capacities of T24 cells as determined by EXTIM. The representative pictures show mock-treated (DMSO control) and compound-treated T24 cells, which were stained in the cell culture well after transmigration through the mouse bladder. From left to right, T24 cells were treated with DMSO, MMP3i, COX-2i, or ETA-a at days 8 (top row) and 10 (bottom row). *n* = 3 independent experiments were performed. The bar graph illustrates the first day on which transmigration of the indicated cells through the mouse bladder was detected during the observation time in three independent repeat EXTIM experiments. Each symbol indicates the first day of transmigrating cells in one of the three independent EXTIM experiments. Filled circle: DMSO-treated T24 cells; Filled square: MMP3i-treated T24 cells; Filled upward triangle: COX-2i-treated T24 cells; Filled downward triangle: ETA-a-treated T24 cells. **G**. The bar graph indicates the tissue-culture surface area occupied by colonies of transmigrated T24 cells in EXTIM as calculated in µm^2^. Cells were treated with DMSO, MMP3i, COX-2i, or ETA-a. *P < 0.05, **P < 0.01. **H**. Evaluation of the toxicity of the indicated compounds (MMP3i, COX-2i, and ETA-a) on T24 cells as determined by the MTT assay on days 1, 2, 4, 6, 8, and 10 post-seeding. The relative number of living T24 cells treated with the indicated compounds is shown by growth curves (see “Materials and methods” for calculations). The bar graphs display the mean ± SEM values, and a two-tailed Student’s *t* test was used to assess significance. ****P < 0.0001. MTT assays were performed in sextuplicate. **I** *EDNRA* (ETA) mRNA levels in NMIBC versus MIBC in GSE13507 and GSE32894 datasets. NMIBC, non-muscle invasive bladder cancer. MIBC, muscle invasive bladder cancer. The microarray datasets GSE13507 and GSE32894 were obtained from the Gene Expression Omnibus (GEO) database (https://www.ncbi.nlm.nih.gov/). GSE13507 contained NMIBC (*n* = 103) and MIBC (*n* = 62). GSE32894 contained NMIBC (*n* = 213) and MIBC (*n* = 93). Significance was assessed using the unpaired *t* test. A *P*-value < 0.05 was considered statistically significant. ***P < 0.001, ****P < 0.0001. **J** Kaplan‒Meier survival curve showing overall survival (OS) for patients with BC in correlation with *EDNRA* mRNA levels, stratified by expression level. The source data [33] were downloaded from the cBioPortal [34, 35], and OS was calculated via PRISM software. Patients were stratified into high (*n* = 101) and moderate/low (*n* = 303) EDNRA expression groups using the 75% percentile as the cutoff. The high *EDNRA* group showed significantly poorer overall survival compared with the moderate/low *EDNRA* group (*P* = 0.006, log-rank test). Note: OS is plotted against time in months

We aimed to identify genes associated with cell invasion via a CRISPR-Cas9 screening. Although the Boyden chamber method has been previously used for this purpose, it is not ideal for identifying genes specifically related to invasion [18–20]. This is because cells with high migratory but low invasive capacities can also be enriched, as demonstrated above. Also the porcine bladder model, well-established in our laboratory, was unsuitable for this purpose owing to technical reasons. To overcome these challenges, we developed the ex vivo mouse bladder tissue invasion model (EXTIM). The setup procedure is demonstrated in Fig. 2A and in the accompanying video file (Supplementary Video), with the detailed protocol provided in the “Materials and methods” section. Briefly, bladders were collected from C57BL/6 J mice aged between 17 and 24 weeks (Fig. 2A–1). After an incision was made at the neck of the bladder (Fig. 2A–2), a small hollow cylinder was inserted into the bladder lumen (Fig. 2A–3). To ensure that the bottom opening of the small hollow cylinder was completely covered by bladder tissue, the assembly was then inserted into a larger hollow cylinder to secure the bladder tissue and prevent seeded cell leakage (Fig. 2A–4, 5). The entire assembly consisting of cylinders and mouse bladders was then placed into an insert (12-well) and cultured in a 12-well cell culture plate (Fig. 2A–6) with complete medium until the cells transmigrated through the mouse bladder tissue (Fig. 2A–7, 8). Importantly, the whole setup was moved to a new well with fresh complete medium every second day (Fig. 2B–1–6). After the EXTIM setup, the previous 12-well plates were maintained with 2 mL of fresh complete medium for 10‒20 days and subsequently stained with Crystal Violet to visualize cancer cells that eventually transmigrated through the mouse bladder cells (Fig. 2B‒5), while the EXTIM setup continued in a new plate (Fig. 2B‒6). Notably, we were able to culture the mouse bladders for up to 190 days without any complications.

Note that, in the following, the term “transmigration”’ is used here solely to describe the crossing of the established cancer cell lines through the ex vivo mouse bladder tissue, a process that involves cancer cell migration and invasion.

### EXTIM is suitable to assess the impact of gene alterations on the invasive capacity of cells

Before initiating CRISPR screening via EXTIM, we evaluated whether it is suitable for investigating the different invasive capacities of BC cells, including genetically modified BC cells with specific gene alterations. Notably, highly invasive T24 and UMUC3 cells transmigrated through mouse bladders in all independent experiments (Fig. 2C, D). First invasive colonies were observed at day 4 for T24 cells and at day 6 for UMUC3 cells (Fig. 2E). Importantly, the number of invasive colonies per well (i.e., per time point) increased over time (Fig. 2C, e.g., comparing colony numbers on days 26, 28, and 30). On the other hand, neither RT4 nor Y235T cells transmigrated through the mouse bladder even after 60 days in culture in the EXTIM (Fig. 2D, E).

Next, on the basis of recent reports that the connexin protein GJB3 and the oxysterol-binding protein ORP3 suppress BC cell migration and invasion [14, 15], we knocked down GJB3 or ORP3 in RT4 cells (Supplementary Fig. SF2A) and examined the invasive behavior of the modified cell lines (RT4-shScr, RT-shORP3, and RT4-shGJB3) via EXTIM (Fig. 2F). While RT4-shScr cells did not transmigrate through the bladders of the mice during the observation period (up to 94 days in culture), RT4-shGJB3 or RT4-shORP3 cells transmigrated on days 88 and 72 post-seeding, respectively (Fig. 2F). Conversely, ectopic expression of GJB3 or ORP3 in T24 cells impaired their transmigration ability (Fig. 2G, Supplementary Fig. SF2B). T24-EV, T24-GJB3, or T24-ORP3 cells transmigrated on day 21, 37, and 39 (Fig. 2F).

### p53 abrogation in RT4 cells for CRISPR-Cas9 screening

To perform genetic screening to identify genes that influence the ability of cells to transmigrate through bladder tissue, we choose RT4 cells as they are unable to transmigrate through the mouse bladder in the EXTIM. As the presence of the tumor suppressor protein p53 can impair CRISPR-based gene editing [21], we functionally blocked p53 activity via an ectopic dominant negative p53 protein (p53DD) in RT4 cells (designated RT4-TP53DD) (Fig. 3A). Given that the most invasive BC samples and cell lines lack p53, or one of its pathway members, we first tested whether the functional inactivation of p53 in RT4 cells would affect their invasive ability via our experimental approaches. The results from both the Boyden chamber assays and the EXTIM experiments revealed that p53 inactivation did not significantly influence the invasive ability of RT4 cells (Fig. 3B–E). Importantly, we also assessed the invasive ability of genetically altered RT4 cells (GJB3-KD or ORP3-KD) in the EXTIM, alongside their parental and p53-inactive counterparts (Fig. 3C). The results indicated that RT4-TP53DD cells with GJB3 or ORP3 abrogation (RT4-TP53DD-shGJB3 and RT4-TP53DD-shORP3; see Supplementary Fig. SF3) were able to transmigrate through the mouse bladder, similar to their counterparts with active p53 (RT4-shGJB3 and RT4-shORP3). In contrast, the parental RT4 and RT4-TP53DD strains, as well as their control counterparts with scrambled shRNAs (RT4-shScr and RT4-TP53DD-shScr), did not transmigrate through the mouse bladder in the EXTIM during the observation time (invasive events: 0/2 for RT4, 0/2 for RT4-TP53DD, 0/4 for RT4-shScr, 0/4 for RT4-TP53DD-shScr, 4/8 for RT4-shGJB3, 2/4 for RT4-TP53DD-shGJB3, 4/8 for RT4-shORP3, and 2/5 for RT4-TP53DD-shORP3) (Fig. 3D). Notably, we observed slightly faster transmigration of RT4 cells with GJB3 or ORP3 knockdown in combination with nonfunctional p53 than in parental RT4 cells (transmigration time: 82 days for RT4-shGJB3 versus 66 days for RT4-TP53DD-shGJB3, *P* = 0.554; and 139 days for RT4-shORP3 versus 110 days for RT4-TP53DD-shORP3, *P* = 0.548) (Fig. 3E).

### CRISPR-Cas9 screening via EXTIM identified genes involved in BC cell invasion

To further evaluate whether EXTIM could be applied to effectively isolate invasive cells from a mixture of noninvasive and invasive cells, noninvasive RT4 cells and highly invasive eGFP-expressing UMUC3 cells (UMUC3-eGFP) were mixed at a ratio of 1000:1. Indeed, only UMUC3-eGFP cells transmigrated through the mouse bladder (Supplementary Fig. SF1), providing additional support that only invasive cells are able to transmigrate through the mouse bladder and excludes any cross-contamination or leakage in the EXTIM.

The overall screening procedure is depicted in Fig. 4A. To identify genes that suppress BC cell invasion, RT4-TP53DD cells were infected with a genomewide CRISPR/Cas-9 library consisting of 65,383 single guide RNAs (gRNAs) targeting 19,050 genes and 1864 microRNAs (Fig. 4A–1, 2). Notably, the library also contained 1000 nonsense gRNAs as an internal control. The cells were subjected to antibiotic selection for 14 days and subsequently seeded in 30 EXTIM setups (Fig. 4A–3). In parallel, we also used one EXTIM setup with RT4-TP53DD cells and two EXTIM setups with RT4-TP53DD cells infected with two different control gRNAs, directed against the green fluorescence protein (GFP)-encoding mRNAs (gGFP#1 and gGFP#2). As described above, during culture, the EXTIM setup was moved to the next well every second day. This procedure was continued for 40 days, and the mouse bladders were ultimately removed at this time point (Fig. 4B). Importantly, we did not observe any invading cells in the control EXTIM setups (i.e., with RT4-TP53DD, RT4-TP53DD-gGFP#1, and RT4-TP53DD-gGFP#2). In the library group, we observed the growth of transmigrated cells in 11 out of 30 EXTIM setups (Fig. 4B, left). Notably, cells that transmigrated into the mouse bladder were observed on day 30 in well 2; day 32 in wells 1 and 8; day 34 in wells 5, 6 and 10; day 36 in well 4; and day 38 in well 3. Finally, on day 40, the cells were cultured in wells 7, 9, and 11. We stopped further culture at the next time point, on day 42. To exclude false positives and strengthen our results, we decided to reseed the cells from each well into a new EXTIM setup (Fig. 4B, right). Notably, we did not attempt to pick single clones from all the wells for two reasons: first, in some cases, colonies were too close to each other; second, the number of cells was still too low, and any handling could result in their loss, which occurred when we tried to pick single clones from well 1. For the control cells, we seeded fresh RT4-TP53DD and RT4-TP53DD gGFP#1 cells (Fig. 4B, right). Following the second seeding, we did not observe any transmigration of the control cells in the EXTIM. Notably, together with all the above-mentioned control experiments (Fig. 3), these repeated experiments also revealed that there was not even a single transmigratory cell among the parental RT4 or RT4-TP53DD cells. Finally, in 7 of the 11 EXTIM experiments, we observed transmigration and clone growth. In detail, colonies in wells 1 and 2 were identified on day 24; those in wells 5 and 10 were identified on day 28; and those in wells 8, 6 and 4 were identified on days 26, 30 and 32 (Supplementary Table ST1). These findings strongly indicate that these cells may contain gRNAs, altering the invasive capacity of RT4-TP53DD cells. To identify these candidate genes, we collected these cells, isolated genomic DNA and amplified the gRNA sequences with primers surrounding the region of the gRNA sequence (Fig. 4A–5, 6). On the basis of previous experiments, we first cloned the PCR products into the TOPO-TA vector for sequencing (Fig. 4A–7, 8, 9, 10).

Finally, 34 unique guide RNA sequences (note that RFLP2 was found in two different wells independently, wells 2 and 5, respectively) were identified, targeting both protein-coding genes and microRNAs (Supplementary Table ST2). These candidates included microtubule function-related genes, such as KIFC2 [22] and BICD1 [23], as well as actin function-related genes, such as FMOD [24] and DDR1 [25]. Among these candidates, 31 out of all 34 candidates have been reported to be associated with cancer, whereas 21 of these genes, such as DDR1 [26, 27], FERMT1 [28], AZGP1 [29], and ABCA3 [30], have been found to regulate cell migration and invasion via epithelial–mesenchymal transition (marked in green in Supplementary Table ST2), and are associated with cancer. For 3 of 34 candidates, mir-6795, NSMF, and RFPL2, there are currently no reports related to cancer, and these genes need to be further explored in the field of cancer research. In summary, our screening results strongly support the involvement of the identified candidate genes in cancer initiation and/or progression and that the majority of them have been shown to be related to cancer cell migration and invasion.

### Bioinformatic and gene expression analyses point to DDR1 as a top candidate

To select suitable candidates for subsequent verification experiments, we first conducted bioinformatic analyses using two different datasets with BC gene expression data, which included noninvasive and invasive BC samples (GSE32894 [31] and GSE13507 [32], respectively). Notably, for microRNAs, we failed to find appropriate datasets that included both MIBC and NMIBC samples for miRNA studies, as microRNA sequencing was performed on either MIBC or NMIBC cohorts. Of the 28 protein-coding genes identified, 11 (*DDR1*, *AZGP1*, *STK19*, *STEAP3*, *LAT*, *IL11*, *FERMT1*, *TMEM18*, *EMP2*, *PRRT3*, and *BICD1*) were found to be downregulated in MIBC compared with NMIBC in at least one dataset. However, only five (*DDR1*, *AZGP1*, *STK19*, *STEAP3*, and *LAT*) were significantly downregulated in MIBC relative to NMIBC in both datasets (Fig. 5A, B). Next, we used Gene Ontology (GO) analysis (Supplementary Table ST3) and determined the expression of the five genes in noninvasive and invasive BC cell lines (Supplementary Fig. SF4A), as well as in a set of apparently healthy human tissues and human ureter-derived epithelial cells (Supplementary Fig. SF4B). Interestingly, DDR1 mRNA was highly expressed in the human ureter epithelium compared with all the other human tissue samples (Supplementary Fig. SF4B). In summary, these analyses identified DDR1 as a top candidate with a role in normal bladder epithelium and suggested that DDR1 may have an inhibitory function in the progression of BC to MIBC.

In line with the results of the in silico analyses and initial gene expression results, DDR1 mRNA and protein levels were lower in invasive BC cell lines (T24, UMUC3, and BFTC-905) than in non/low-invasive BC cell lines (RT4, BC61, and 5637) and in ureter-derived immortal cell strains (Y235T, HBLAK, and UROtsa) (Fig. 5C, D). Although the DDR1 protein levels somewhat differed from the mRNA levels, the DDR1 levels were clearly higher in the widely used, noninvasive BC cell lines RT4 and BC61 than in the widely used, highly invasive BC cell lines T24 and UMUC3 (Fig. 5C, D). These data indicate a prominent function of DDR1 in healthy ureter/bladder epithelium and that downregulation of DDR1 is associated with the invasive progression of human BC. Moreover, a comparison of *DDR1* mRNA levels via a TCGA dataset [33], which was downloaded from the cBioPortal [34, 35] and comprises data from patients with invasive BC, revealed that lower *DDR1* mRNA levels are associated with reduced overall survival of male patients with BC but not female patients with BC (Fig. 5E). In summary, these data support the idea that DDR1 may have a tumor suppressive role in the bladder epithelium.

### Alterations in DDR1 impact the invasive capacity of BC cell lines

To investigate the role of DDR1 on BC cell invasion, DDR1 was knocked out in RT4-TP53DD cells via CRISPR/Cas-9 employing two independent gRNAs. One of these gRNAs (gDDR1#1) was identified during our screening (Fig. 6A). On the other hand, DDR1 was ectopically expressed in T24 cells (Fig. 6B). We first used the Boyden chamber assay to assess the impact of DDR1 alterations on the migratory/invasive characteristics of these cells. Indeed, DDR1 depletion in RT4-TP53DD cells significantly increased their migration/invasion potential in this experimental system (3.7 and 4.4 cells/field with RT4-TP53DD-gGFP#1 or with RT4-TP53DD-gGFP#2, versus 418.5 and 460.5 cells/field with RT4-TP53DD-gDDR1#1 and RT4-TP53DD-gDDR1#2, *P* < 0.0001; Fig. 6C, D). Conversely, ectopic DDR1 expression in T24 cells markedly reduced cell motility and invasive capacity (462.4 cells/field with T24-EV versus 192.3 cells/field with T24-DDR1, *P* < 0.0001; Fig. 6E, F).

Next, the impact of DDR1 on the invasive capacity of BC cell lines was investigated via the ex vivo porcine bladder system. In line with our previous results, RT4-TP53DD cells expressing gGFP#1 did not invade porcine bladder tissue, whereas RT4-TP53DD cells lacking DDR1 invaded porcine bladder tissue (the invasive distance was 0 µm for RT4-TP53DD gGFP#1 cells and 90.5 µm for RT4-TP53DD-gDDR1#1 cells on day 14; *P* < 0.0001; Fig. 6G, H). Conversely, ectopic DDR1 expression in T24 cells significantly impaired their invasive capacity in the ex vivo porcine bladder approach (reducing the invasive distance from 152.3 µm for T24-EV to 11.45 µm for T24-DDR1, *P* < 0.0001; Fig. 6I, J).

Finally, we used EXTIM to determine whether DDR1 deficiency in RT4-TP53DD cells or ectopic DDR1 expression in T24 cells could account for the observed changes in invasive capacity. Consistent with the results from the Boyden chamber assay and porcine bladder invasion model, DDR1 knockout significantly promoted transmigration through the mouse bladder in EXTIMs (no transmigration of RT4-TP53DD-gGFP, *n* = 3, versus 3/3 transmigration of RT4-TP53DD-gDDR1, *n* = 3, over 24.6 days on average, *P* < 0.0001) (Fig. 6K, L). Conversely, ectopic DDR1 expression in T24 cells impaired their ability to transmigrate through the mouse bladder in EXTIMs (transmigration for 16 days on average for T24-EVs, *n* = 3, versus 23.3 days for T24-DDR1, *n* = 3, *P* = 0.0082) (Fig. 6M, N). These observations further support the role of DDR1 in regulating the invasive progression of BC cells.

### Small-molecule inhibition of DDR1 improves the invasive capacity of BC cell lines

Several DDR1 inhibitor molecules have been developed to treat patients with fibrotic diseases, as dysregulation of DDR1 is thought to contribute to the development of fibrotic diseases (kidney, lung, and liver fibrosis) [36]. Among these, VU6015929 is one of the most promising potent and selective inhibitors and inhibits both DDR receptors DDR1 and DDR2, with half-maximal inhibition concentration (IC_50_) of 4.67 nM and 7.39 nM [37, 38]. As our above results indicate that DDR1 inhibits the invasive ability of BC cells and that DDR1 levels are downregulated in several invasive BC cell lines [39], the impact of this inhibitor on BC cell invasion was investigated. For this purpose, we used T24 cells with ectopic DDR1, as this cell line lacks both DDR1 and DDR2 expression (Fig. 7A, B; see also Fig. 5C, D). Similar to our results presented in Fig. 6, here, we again show that ectopic DDR1 impairs the invasive capacity of T24 cells (Fig. 7C, D). Notably, the impaired migratory/invasive capacity of T24-DDR1 cells (T24-DDR1) could be significantly reversed by treatment with the DDR1 inhibitor VU6015929 in the Boyden chamber assay (136.5 cells/field with T24-DDR1 versus 382.3 cells with the T24-DDR1 + DDR1 inhibitor). *P* < 0.0001) (Fig. 7C, D). Importantly, the DDR1 inhibitor VU6015929 markedly relieved the DDR1-dependent impairment of the transmigration ability of T24 cells in EXTIMs (Fig. 7E). Since T24 cells lack the DDR2 protein, these results suggest that DDR1 genuinely exerts a suppressive effect on invasion and migration.

### A combined small-compound library screen using a Boyden chamber and EXTIM identifies the role of ETA in BC cell invasion

Inspired by the results with the DDR1 inhibitor compound, we proceeded to explore whether we might have examined the impact of other small-molecule drugs in EXTIMs with similar characteristics. To apply an unbiased approach with a feasible set of compounds, we used a library of small chemical compounds that contained 90 drugs developed by Pfizer (LOPAC Pfizer) (see Supplementary Table ST6 for the full list). First, using T24 cells, we tested the effects of all the compounds on cell viability to exclude compounds that significantly influence cell proliferation or survival within 4 days of treatment at a concentration of 10 µM and found that 62 compounds had no or only mild impact on cell viability under these conditions (Fig. 8A, Supplementary Fig. SF5). Subsequently, the impact of these 62 compounds on the migration/invasion ability of T24 cells was investigated via a Boyden chamber assay (Fig. 8A, Supplementary Fig. SF6). Among these, three compounds, the matrix metalloprotease 3 (MMP3: position F2 in the 96-well plate) inhibitor (MMP3i: UK-356618), the cyclooxygenase-2 (COX-2: position B12 in the 96-well plate) inhibitor (COX-2i: SC-236), and the antagonist of endothelin receptor A (ETA: position E4 in the 96-well plate) (ETA-a: PD-156707) impaired the invasive capacity of T24 cells. The cell viability and Boyden chamber assays were repeated for these compounds, confirming the first observations (Fig. 8B–D). Finally, we employed the EXTIM approach to investigate the impact of these compounds on the transmigration capacity of T24 cells. The results obtained with the COX-2 inhibitor do not allow a definitive conclusion regarding the role of COX-2 in regulating cell migration and invasion (Fig. 8C–H). In fact, we observed an overall reduction of transmigratory T24 cells in the COX-2 inhibitor group (Fig. 8E, G), indicating that COX-2 inhibition may impair transmigration capacity of T24 in EXTIM, which is consistent with the results of the Boyden chamber experiments (Fig. 8C, D). However, first clones of transmigratory cells in the COX-2 inhibitor-treated T24 group showed up around day 5, like the control DMSO-treated T24 cells, indicating that COX-2 inhibition does not affect the capacity of T24 cells to transmigrate in EXTIM. It is plausible to assume that the reduced cell transmigration capacity (Fig. 8E, G) with COX-2 inhibitor is, at least in part, due to some toxicity of the compound after long-term treatment (Fig. 8H). Likely, although the MMP3 inhibitor seemed to suppress T24 transmigration in EXTIMs, long-term cell survival analysis revealed that the MMP3 inhibitor impaired cell survival when the cells were treated for longer than 4 days (Fig. 8H). In summary, among the 90 compounds tested, only the ETA antagonist compound impaired T24 cell transmigration in EXTIMs (Fig. 8E–H).

Importantly, PD-156707 has a binding affinity (*K*_i_) of 0.17 nM for the human ETA receptor, whereas its affinity for endothelin receptor type B (ETB) is significantly lower, with a *K*_i_ of 133.8 nM, indicating over 780-fold selectivity for ETA over ETB receptors [40]. Although we observed reduced cell migration and invasion at 100 nm to 1 µm PD-156707 concentrations in the Boyden chamber with T24 cells, which express high levels of *ETA* but very low levels of *ETB*, the EXTIM experiments were conducted at 10 µM concentrations to ensure observable effects within a relatively short timeframe (Supplementary Fig. SF7). Notably, UMUC3 cells, which express detectable levels of *ETB* but not *ETA* mRNA, showed only partial impairment of migration and invasion at 10 µM PD-156707 in the Boyden chamber assay (Supplementary Fig. SF7C, D). Despite these observations, the impact of ETB on the invasive capacity of BC cell lines appears to be less pronounced than that of ETA, given that ETB expression is greater in the non-invasive BC cell lines RT4 and BC61 than in the highly invasive BC cell lines T24 and BFTC-905.

Next, we analyzed gene expression databases to elucidate the association of ETA (encoded by the *EDNRA* gene), ETB (encoded by the *EDNRB* gene), MMP3 (encoded by the *MMP3* gene), and COX-2 (ETB, encoded by the *PTGS2* gene) with BC. For this purpose, we used the above-described databases GSE13507 and GSE32894 to compare their expression profiles in NMIBC versus MIBC and the TCGA dataset by Robertson et al. [33] to correlate overall survival of patients with BC in relation to the mRNA levels of these genes (Fig. 8I, J and Supplementary Fig. SF8). For the survival analyses, transcript abundance rank values were defined as “low/moderate” (0–74) or “high” (75–100) percentile RNA expression ranks. Among these genes, only *EDNRA* (ETA) showed significant increase in both NMIBC/MIBC comparisons and reduced OS survival rates with increased gene expression levels (Fig. 8I, J and Supplementary Fig. SF8). Interestingly, although *MMP3* levels were higher in MIBC, OS analysis did not reveal an association between *MMP3* levels and OS of patients with BC (Supplementary Fig. SF8). Surprisingly, while PTGS2 (COX-2) levels tended to be higher in patients with MIBC compared with patients with NMIBC, patients with higher PTGS2 levels showed better survival rates compared with patients with lower PTGS2 levels (Supplementary Fig. SF8). In conclusion, the presented data demonstrate that ETA (EDNRA) could be a suitable candidate gene for BC diagnosis and treatment.

## Discussion

The processes of cancer cell migration, invasion, and metastasis are crucial for tumor progression and significantly impact patient outcomes [41, 42]. These processes involve a complex interplay of various genetic, molecular, and environmental factors that contribute to tumor aggressiveness [43]. Despite significant advances in BC research that have identified several key cellular processes involved in MIBC progression, such as the epithelial‒mesenchymal transition (EMT) process, which involves the loss of epithelial markers such as E-cadherin and the acquisition of mesenchymal markers such as N-cadherin and vimentin, the determinants of BC invasion remain elusive [44], to some extent owing to the lack of appropriate experimental models. In this study, we developed a novel ex vivo tissue invasion model (EXTIM) as a tool for investigating the migration and invasive capacity of BC cells in an intact tissue context. EXTIM utilizes culturing of largely intact mouse bladders over a period of up to 190 days ex vivo. The cells seeded into the mouse bladder can transmigrate through the bladder tissue and can be further cultured for subsequent downstream analyses. Importantly, we utilized this innovative model in combination with a CRISPR-Cas9 library and a limited library of 90 compounds to identify novel candidate molecules involved in the cell migration and invasion process. While CRISPR-Cas9 screening identified DDR1 as a potential inhibitor of BC cell migration, compound screening revealed that the endothelin receptor ETA promotes the BC cell invasion process, as PD-156707, a selective ETA antagonist, inhibits cancer cell invasion and transmigration in EXTIMs.

The new ex vivo cancer cell invasive model EXTIM provides significant advantages over the existing Boyden chamber and other ex vivo tissue invasion models that utilize rat, porcine, or human ureter tissues to investigate tumor cell spreading, as summarized in Supplementary Table ST4. Moreover, EXTIM is suitable to evaluate the impact of specific gene alterations on these processes, as demonstrated by alterations of GJB3 or ORP3, supporting the idea that this tool could be applied as a genetic screening approach for identifying genes involved in BC invasion/spread. In fact, using EXTIM in combination with genomewide CRISPR-Cas9 library screening, we identified several candidate genes with potential roles in cancer cell migration and invasion. Subsequent bioinformatics analysis via high-throughput sequencing of published datasets of NMIBC and MIBC samples, accompanied by gene expression studies, identified *DDR1* as the top candidate gene.

The discoidin domain receptors (DDRs), including DDR1 and DDR2, belong to the family of receptor tyrosine kinases (RTKs), which are single-pass transmembrane receptors containing extracellular ligand-binding regions and conserved cytosolic kinase domains [45]. Under physiological conditions, DDR1 is expressed mainly in epithelial cells and is involved in several key cellular processes, including cell migration, cell adhesion, signalling, ECM interactions, and cell proliferation [46]. DDR1 functions by binding to collagen and other ECM components, leading to autophosphorylation and activation of downstream signaling pathways that promote cell survival, proliferation, and migration [47]. Importantly, DDR1 has garnered significant attention in cancer research because of its emerging role in tumor progression, particularly in the context of tumor cell migration and invasion. Elevated DDR1 expression has been observed in various types of tumors, including breast cancer [48], gastric cancer [25], non-small cell lung carcinoma (NSCLC) [49], melanoma [50], and colon cancer [51], where it has been associated with more aggressive disease phenotypes. However, recent findings suggest that DDR1 may have both promigratory and antimigratory effects, indicating a more complex role in cancer biology than initially thought [52]. For example, DDR1 has been shown to stabilize cell‒cell junctions in epithelial cells, reducing their motility and invasive potential [53]. These findings suggest that DDR1 may play a dual role in different cancer types or at different stages of tumor progression. In some cases, DDR1 signalling may maintain epithelial integrity and act as a barrier to epithelial‒mesenchymal transition (EMT), a critical process for metastasis [54]. Importantly, a study in DDR1 knockout mice revealed that DDR1 ablation induces aggressive basal-like breast cancers, supporting the tumor-suppressive function of DDR1 in epithelial cells [55].

Our findings are rather in concordance with a tumor-inhibitory or migration/invasion inhibitory role of DDR1 in bladder tissue. We detected high DDR1 expression in normal epithelial cells of the human ureter and noninvasive bladder cancer cell lines (RT4 and BC61), whereas DDR1 levels were low/absent in invasive BC cell lines (e.g., T24 and UMUC3). We showed that DDR1 knockout resulted in increased invasiveness of RT4 cells in the Boyden chamber, porcine bladder, and EXTIM models, whereas ectopic DDR1 expression impaired the migratory and invasive/transmigratory capacity of T24 cells. Importantly, a commercially available DDR1 inhibitor, which was developed to block DDR1 activity for tumor therapy, reversed the migration/invasion inhibition of ectopic DDR1 (i.e., T24-DDR1). Our conclusions are further supported by high-throughput sequencing results retrieved from the TCGA database, which revealed that lower DDR1 levels are associated with poorer survival. Interestingly, we found an association for worse survival for male but for not female patients with BC. Although we cannot provide a definitive explanation for this gender-specific outcome, several factors may underlie the absence of an observed association between low DDR1 expression and poorer survival among female patients with bladder cancer (BC). One plausible explanation is the limited sample size (Fig. 5E) and corresponding lack of statistical power, as BC occurs approximately four times more frequently in men than in women. Notably, given that female patients with BC often present with more advanced tumor stage at diagnosis and exhibit worse stage-matched survival, the differential prognostic impact of DDR1 may reflect underlying sex-specific biological and biomechanical mechanisms contributing to outcome disparities in BC. Whether sex-based differences in molecular tumor subtypes underlie these survival differences remains to be elucidated. The use of DDR1 inhibitors has emerged as a potential therapeutic strategy aimed at reducing tumor metastasis and improving the efficacy of existing therapies [56–58]. However, given its dual role in promoting and inhibiting migration, the therapeutic targeting of DDR1 must be approached with caution. Inhibiting DDR1 in tumors, where it plays a protective role in preventing metastasis, could worsen patient outcomes by enhancing EMT and invasion. In fact, more research is needed to distinguish between the pro- and antimigratory functions of DDR1 in different cancer types and contexts.

Our compound screening approach combining the Boyden chamber and EXTIM assays surprisingly revealed that PD-156707, a selective endothelin receptor type A (ETA) antagonist, inhibited the migration/invasion and transmigration processes in the Boyden chamber and EXTIM approaches. ETA is a G protein-coupled receptor that binds endothelin-1 (ET-1) and mediates vasoconstriction. However, several reports have also indicated the role of ETA in cell migration and metastasis across various cancer types [59–61]. Interestingly, elevated ET-1 levels in bladder cancer patients were associated with increased lung metastases and decreased survival. In line with this observation, ET-1 was demonstrated to act through ETA to enhance tumor cell and macrophage migration, invasion, and the expression of inflammatory cytokines and proteases, facilitating metastatic colonization [59]. Moreover, ambrisentan, another endothelin receptor type A-selective antagonist, inhibits cancer cell migration, invasion, and metastasis [62]. These studies collectively highlight the pivotal role of ETA in cancer cell migration and metastasis, suggesting that targeting ETA could be a promising therapeutic strategy in oncology. We noted that PD-156707 at a concentration of 10 µM also impaired the migration/invasion capacity of UMUC3 cells lacking ETA expression in the Boyden chamber, indicating that ETB may also be a candidate for suppressing cancer cell invasion and migration. In summary, EXTIM provides an advanced model for studying the early stages of tumor dissemination in a tumor cell–“healthy tissue” context. The development of a faithful model that resembles human bladder cancer invasive progression holds immense potential for advancing our understanding of the mechanisms underlying this process and serves as a valuable tool for identifying and evaluating potential treatment regimens for bladder cancer invasion. Although the EXTIM offers a valuable experimental tool for investigating the invasive progression of BC, several limitations remain. One limitation of the mouse bladder model is the inherent anatomical and physiological differences between mouse and human bladders. These disparities may affect the translation of findings from mouse studies to human clinical settings. Additionally, while the mouse bladder model can provide valuable insights into BC invasion, it may not fully recapitulate the complexity of human bladder cancer progression. Most critically, a functional immune response is lacking under ex vivo conditions.

Despite these limitations, EXTIM closely approximates physiological conditions and provides a tissue microenvironment for studying the BC invasion process. Notably, cells seeded into the EXTIM can transmigrate through bladder tissue and can be further cultured for downstream analyses. Most importantly, EXTIM provides an applicable tool for high-throughput analyses, as demonstrated previously. In this study, we used C57BL/6 mice without any genetic modifications. However, it is conceivable that bladder tissue from genetically modified mice, such as E-cadherin knockout mice or transgenic mice with altered stromal functions, in combination with noninvasive or invasive cancer cell lines could be used to investigate the contribution of the specific tissue microenvironment to tumor cell dissemination. It would also be tempting to test human cancer cells from different tissue origins in the EXTIM.

## Conclusions

This work establishes EXTIM as a novel and versatile ex vivo tool to study BC invasion within an intact tissue microenvironment. The model enables functional and molecular studies of invasive tumor behavior and allows high-throughput approaches to identify molecules targeting cancer cell dissemination. By combining EXTIM with CRISPR-Cas9 screening, we identified DDR1 as an inhibitor of BC cell migration and invasion. Functional validation confirmed that loss of DDR1 enhanced invasiveness, whereas its ectopic expression constrained migration and invasion capacities. Importantly, clinical data corroborated the biological relevance of DDR1, linking reduced expression to poorer survival outcomes in patients with BC. These findings align with emerging evidence supporting a context-dependent, and in some settings tumor-suppressive, role for DDR1. Our data underscore the need for caution when considering DDR1 as a therapeutic target, given its potential protective role against invasion. In parallel, compound screening identified ETA signaling as a pro-invasive pathway in BC cells. Pharmacological inhibition of ETA significantly reduced migration and invasion in both Boyden chamber and EXTIM assays. These results reinforce the growing recognition of endothelin signaling as a driver of tumor dissemination. While limitations related to species differences and immune absence remain, EXTIM bridges an important gap between in vitro assays and in vivo studies, closely mimicking key aspects of tissue-level invasion. Overall, EXTIM represents a powerful platform for dissecting invasion mechanisms and for preclinical evaluation of anti-invasive therapeutic strategies. Importantly, the model is readily adaptable to genetically modified mouse models and diverse cancer cell types.

## Supplementary Information

Additional file 1: SF1. Control experiment showing that only UMUC3 cells but not RT4 cells transmigrate through the mouse bladder in the EXTIM A. Representative images illustrating the different transmigratory capacities of three different cell groups, RT4, UMUC3, and RT4:UMUC3-eGFP, in the EXTIM. To evaluate whether any RT4 cells could transmigrate through the mouse blade when cocultured with UMUC3 cells, RT4 cells were seeded with eGFP-expressing UMUC3 cells (UMUC3-eGFP) at a 1000-to-1 ratio in mouse bladders. Only UMUC3 parental cells and UMUC3-eGFP cells transmigrated through the mouse bladder and proliferated to form colonies in the culture plates. These cells were fixed and stained in the wells for visualization. *n* = 3 independent experiments were performed. Note that, for each cell group, only one of the repeat experiments is shown (depicted here are example plates of the repeat experiments with the plates showing no colonies until day 66 for the RT4 cell group and example plates of the repeat experiments with the plates showing colonies on day 60 for the UMUC3 cell group and RT4 mixed with the UMUC3-eGFP cell group). B Note that pictures were taken before fixation of cells. (Top) The cells were observed under a fluorescence microscope with blue excitation. (Bottom) Pictures of phase contrast microscopy. Scale bars: 500 μm (B). Images captured at total magnification of 50× (B). The representative images show that only UMUC3-eGFP cells but not RT4 cells are able to transmigrate through the mouse bladder and proliferate in the culture wells, forming colonies.

Additional file 2: SF2. Western blot experiments demonstrating the shRNA-mediated downregulation of GJB3 or ORP3 in RT4 cells as well as their ectopic expression in T24 cells. A. Western blot results indicate ORP3 protein expression (Left) in RT4 cells with shScr, shORP3#1, and shORP3#2 and GJB3 protein expression (Right) in RT4 cells with shScr, shGJB3#1, and shGJB3#2. GAPDH or α-tubulin was used as a loading control. *n* = 3 separate experiments were conducted. B The Western blot results show ORP3 protein expression (left) in T24 cells with ectopic ORP3 expression and GJB3 protein expression (right) in T24 cells with ectopic GJB3 expression. α-Tubulin was used as a loading control. *n* = 3 separate experiments were conducted.

Additional file 3: SF3. Results of Western blot experiments demonstrating shRNA-mediated downregulation of GJB3 or ORP3 in RT4-TP53DD cells. The Western blot results indicate ORP3 protein expression (A) in RT4-TP53DD cells with shScr, shORP3#1, and shORP3#2 and GJB3 protein expression (B) in RT4-TP53DD cells with shScr, shGJB3#1, and shGJB3#2. GAPDH or α-tubulin was used as a loading control. *n* = 3 separate experiments were conducted.

Additional file 4: SF4. Determination of relative DDR1 mRNA levels via RT‒qPCR. A. The bar graph shows the relative *DDR1*, *AZGP1* and *STK19*, *STEAP3*, and *LAT* mRNA levels in non/low-invasive BC cell lines (RT4 and BC61) as well as in highly invasive BC cell lines (T24 and UMUC3). The mRNA levels were normalized to those of *GAPDH*. *n* = 3 independent experiments were performed. The error bars represent the means ± SEMs. ? indicates that no significance was determined owing to the low abundance of these genes. B Bar graph showing the relative *DDR1*, *AZGP1* and *STK19*, *STEAP3* and *LAT* mRNA levels in human tissues. Urothelial cells (UC#1, UC#2, UC#3, UC#4, and UC#5) were isolated from the ureters of five different patients who underwent nephrectomy at Ulm University Hospital. The indicated tissue RNAs were acquired from Clontech (see “Materials and methods” section). The mRNA levels were normalized to those of *GAPDH*. *n* = 3 independent experiments were performed. The error bars represent the means ± SEMs.

Additional file 5: SF5. MTT assay to determine the toxicity of 90 compounds on T24 cells. (Top) The bar graph shows the results of the 3-(4,5-dimethylthiazol-2-yl)-2,5-diphenyltetrazolium (MTT) assay, which was used to determine the survival of T24 cells after treatment with the indicated compounds at 10 µM concentrations. The compounds are indicated by their location in the 96-well plate. A detailed list of the compounds is provided in Supplementary Table 6 (Supplementary Table ST6). The cells were counted at days 1, 2, and 4 post-seeding. The MTT assay was performed at least in sextuplicate. Red asterisks indicate the 62 compounds that did not significantly impair cell viability within 4 days. (Bottom) Table showing the results of the MTT assay in summary. Impairment of cell viability was quantified and results are shown in percent of viability impairment. For the next step, i.e., for the Boyden chamber experiment, all compounds exhibiting over 50% viability impairment were excluded (black). The majority of the compounds (red) did not or only mildly impair cell viability at 2 or 4 days of treatment and were used to assess their influence on cell migration/invasion using Boyden chamber assay.

Additional file 6: SF6. Boyden chamber assay to determine the impact of compounds on T24 cell migration/invasion. Representative pictures of the Boyden chamber assay used to assess the impact of the selected 62 compounds on the invasive capacity of T24 cells. Among these, only three compounds (highlighted by red rectangle borders), the matrix metalloprotease 3 (MMP3: position F2 in the 96-well) inhibitor, the cyclooxygenase-2 (COX-2: position B12 in the 96-well) inhibitor, and the antagonist of endothelin receptor type A (ETA: position E4 in the 96-well), impaired the invasive capacity of T24 cells in the Boyden chamber assay. Notably, the results of the Boyden chamber assay were evaluated at 48 h (2 days) after seeding. Importantly, as shown in SF5, cell viability was not impaired at 2 days of treatment with the compounds marked by red asterisks. The compounds are indicated by their location in the 96-well plate. A detailed list of the compounds is provided in Supplementary Table 6 (ST6).

Additional file 7: SF7. ETA is expressed in invasive BC cell lines and inhibits their migratory and invasive capacities. (A) Relative ETA (*EDNRA*) and (B) ETB (*EDNRB*) mRNA expression levels quantified by qRT-PCR in RT4, B61, T24, UMUC3, and BFTC-905 cell lines. (C). Depicted exemplary pictures demonstrate the different invasive and migratory capacities of T24, UMUC3, and BFTC-905 lines following treatment with DMSO or the ETA inhibitor (PD-156707) at 100 nM, 1 µM, and 10 µM in the Boyden chamber assay. Representative images of at least *n* = 3 independent experiments. Images captured at total magnification of 100×. Scale bars: 200 µm. (D) Quantitation of migratory and invasive capacities of T24, UMUC3, and BFTC-905 cell lines following treatment with DMSO or the ETA inhibitor at 100 nM, 1 µM, and 10 µM. Mean ± SEM values from n = 3 independent experiments are shown in the bar graphs.

Additional file 8: SF8. Expression of EDNRB, *MMP3 and PTGS2 in NMIBC, MIBC and correlation their mRNA levels to patient survival. A.* *EDNRB* (ETA), *MMP3*, and *PTGS2* (COX-2) mRNA levels in NMIBC versus MIBC in GSE13507 and GSE32894 datasets. The microarray datasets GSE13507 and GSE32894 were obtained from the Gene Expression Omnibus (GEO) database (https://www.ncbi.nlm.nih.gov/). GSE13507 contained NMIBC (*n* = 103) and MIBC (*n* = 62). GSE32894 contained NMIBC (*n* = 213) and MIBC (*n* = 93). Significance was assessed using the unpaired *t* test. A *p*-value < 0.05 was considered statistically significant. B Kaplan‒Meier survival curves showing overall survival (OS) for patients with BC in correlation with *EDNRB* (ETA), *MMP3*, and *PTGS2* (COX-2) mRNA levels, stratified by expression level. The source data [33] were downloaded from the cBioPortal [34, 35], and OS was calculated via PRISM software. Patients were stratified into high (*n *= 101) and moderate/low (*n* = 303) EDNRA expression groups using the 75% percentile as the cutoff. Note: OS is plotted against time in months.

Additional file 9: ST1. Table showing which candidate was identified in which well in the first and second EXTIM seeding. Red font indicates that no transmigration was observed in the second seeding.

Additional file 10: ST2. List of candidate genes with potential roles in cancer cell invasion identified by genome-wide CRISPR-Cas9 library screening using the new ex vivo mouse bladder tissue invasion model. Genes with a previously reported role in cell migration and invasion are marked in green. Notably, the RFLP2 gRNA was found independently in wells 2 and 5 (indicated by red font). The last two columns refer to selected references indicating the role of the candidate genes in cancer and in cancer cell migration/invasion. The reference list is included at the end of ST2.

Additional file 11: ST3. Gene Ontology (GO-BP) enrichment analysis was performed via the Enrichr tool [63] (https://maayanlab.cloud/Enrichr/).

Additional file 12: ST4. The applications, advantages, and disadvantages of the Boyden chamber, ex vivo porcine bladder, and EXTIM are compared.

Additional file 13: ST5. List of sequences for cloning primers, for PCR, and for gRNAs.

Additional file 14: ST6. List of chemicals used for screening of compounds with detailed information.

Additional file 15.

## Data Availability

The data that support the findings of this study are available from the corresponding author upon reasonable request.

## Abbreviations

BC: Bladder cancer
COX-2i: COX-2 inhibitor
COX-2/PTGS2: Cyclooxygenase-2/prostaglandin-endoperoxide synthase 2
CRISPR: Clustered regularly interspaced short palindromic repeats
DDR1: Discoidin domain receptor tyrosine kinase 1
DDR2: Discoidin domain receptor tyrosine kinase 2
DMEM: Dulbecco’s modified Eagle medium
ECM: Extracellular matrix
EDNRA/ETA: Endothelin receptor type A
EDNRB/ETB: Endothelin receptor type B
ETA-a: Endothelin receptor A antagonist
EV: Empty vector
EXTIM: Ex vivo tissue invasion model
FACS: Fluorescence-activated cell sorting
FCS: Fetal calf serum
GEO: Gene Expression Omnibus
GJB3: Gap junction protein beta 3
gRNA: Guide-RNA (guide ribonucleic acid)
H&E: Hematoxylin and eosin
HTS: High-throughput screening
IHC: Immunohistochemistry
MIBC: Muscle-invasive bladder cancer
MMP3i: MMP3 inhibitor
MMP3: Matrix metalloproteinase-3
MTT: 3-(4,5-Dimethylthiazol-2-yl)-2,5-diphenyltetrazolium bromide
NMIBC: Non-muscle-invasive bladder cancer
PCR: Polymerase chain reaction
ORP3: Oxysterol-binding protein-related protein 3
OS: Overall survival
qRT-PCR: Quantitative reverse transcription-polymerase chain reaction
