# Supplementary figures and images for: CRISPR and compound screens in a novel ex vivo tissue model identify DDR1 and ETA as regulators of cancer cell invasion
Source: Cell Mol Biol Lett. 2026 May 6;31:84. doi: 10.1186/s11658-026-00936-6 (PMC13277066; doi:10.1186/s11658-026-00936-6)

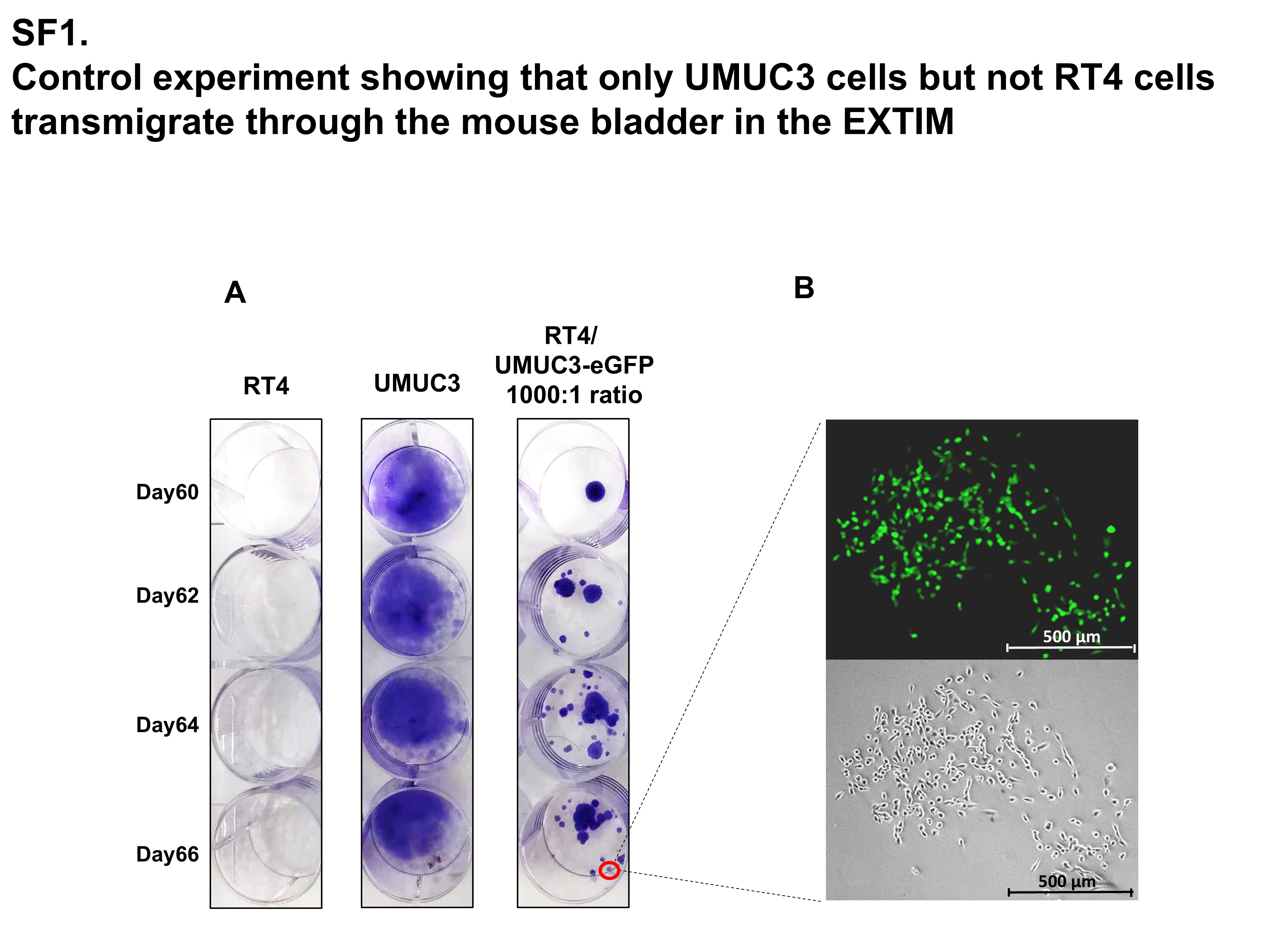

Supplement: Supplementary file 1 — Additional file 1: SF1. Control experiment showing that only UMUC3 cells but not RT4 cells transmigrate through the mouse bladder in the EXTIM A. Representative images illustrating the different transmigratory capacities of three different cell groups, RT4, UMUC3, and RT4:UMUC3-eGFP, in the EXTIM. To evaluate whether any RT4 cells could transmigrate through the mouse blade when cocultured with UMUC3 cells, RT4 cells were seeded with eGFP-expressing UMUC3 cells (UMUC3-eGFP) at a 1000-to-1 ratio in mouse bladders. Only UMUC3 parental cells and UMUC3-eGFP cells transmigrated through the mouse bladder and proliferated to form colonies in the culture plates. These cells were fixed and stained in the wells for visualization. n = 3 independent experiments were performed. Note that, for each cell group, only one of the repeat experiments is shown (depicted here are example plates of the repeat experiments with the plates showing no colonies until day 66 for the RT4 cell group and example plates of the repeat experiments with the plates showing colonies on day 60 for the UMUC3 cell group and RT4 mixed with the UMUC3-eGFP cell group). B Note that pictures were taken before fixation of cells. (Top) The cells were observed under a fluorescence microscope with blue excitation. (Bottom) Pictures of phase contrast microscopy. Scale bars: 500 μm (B). Images captured at total magnification of 50× (B). The representative images show that only UMUC3-eGFP cells but not RT4 cells are able to transmigrate through the mouse bladder and proliferate in the culture wells, forming colonies. [file 11658_2026_936_MOESM2_ESM.tiff]

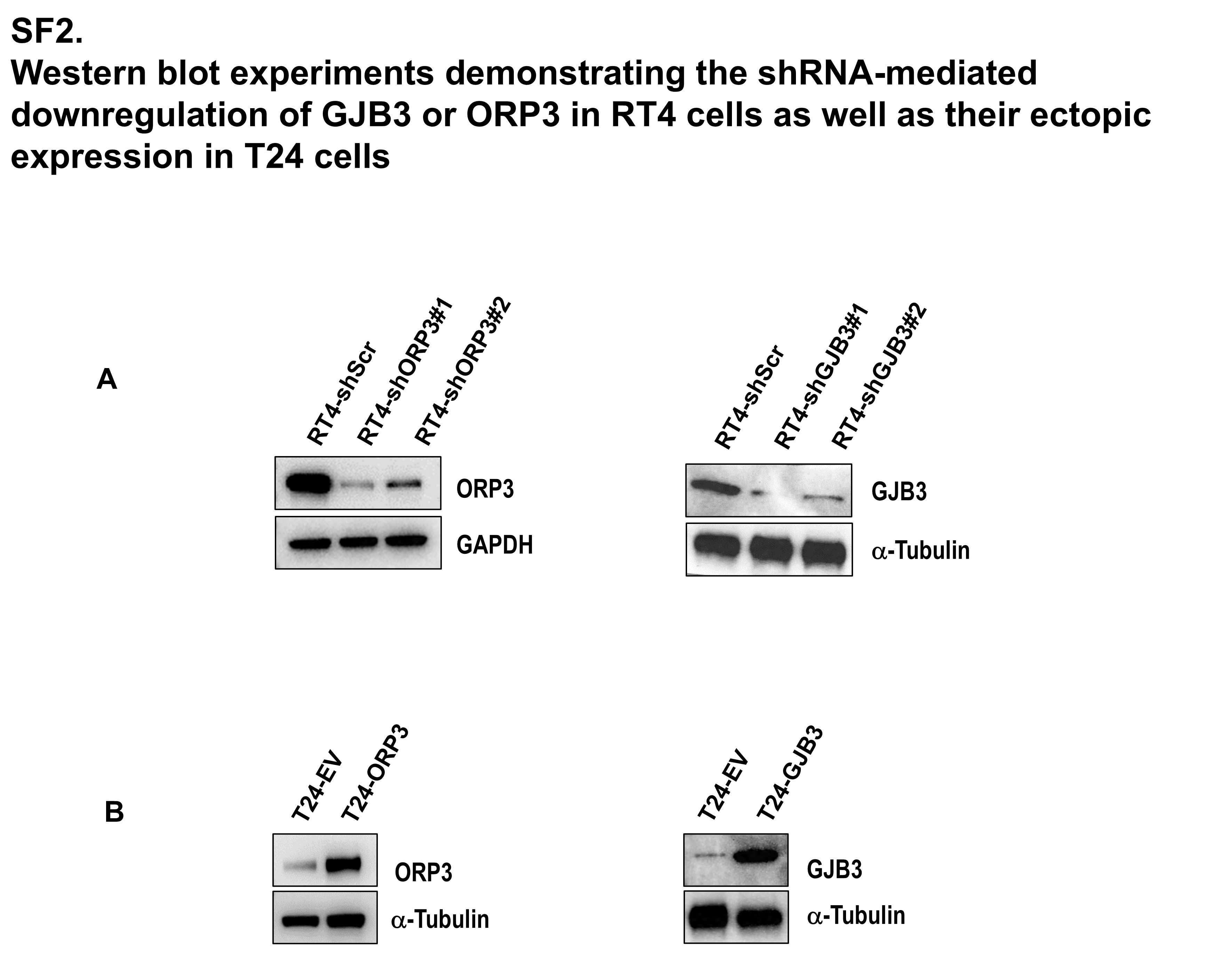

Supplement: Supplementary file 2 — Additional file 2: SF2. Western blot experiments demonstrating the shRNA-mediated downregulation of GJB3 or ORP3 in RT4 cells as well as their ectopic expression in T24 cells. A. Western blot results indicate ORP3 protein expression (Left) in RT4 cells with shScr, shORP3#1, and shORP3#2 and GJB3 protein expression (Right) in RT4 cells with shScr, shGJB3#1, and shGJB3#2. GAPDH or α-tubulin was used as a loading control. n = 3 separate experiments were conducted. B The Western blot results show ORP3 protein expression (left) in T24 cells with ectopic ORP3 expression and GJB3 protein expression (right) in T24 cells with ectopic GJB3 expression. α-Tubulin was used as a loading control. n = 3 separate experiments were conducted. [file 11658_2026_936_MOESM3_ESM.tiff]

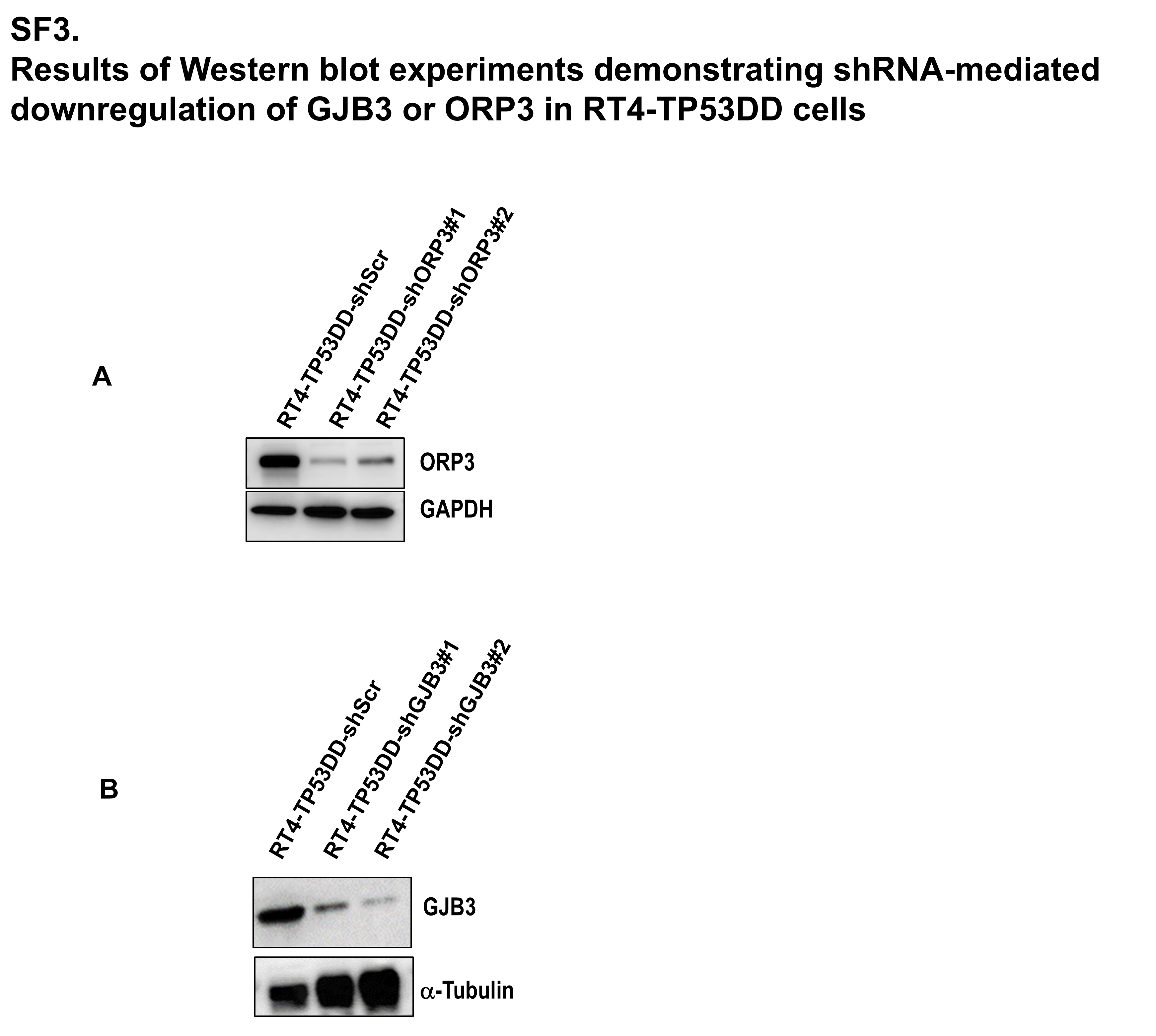

Supplement: Supplementary file 3 — Additional file 3: SF3. Results of Western blot experiments demonstrating shRNA-mediated downregulation of GJB3 or ORP3 in RT4-TP53DD cells. The Western blot results indicate ORP3 protein expression (A) in RT4-TP53DD cells with shScr, shORP3#1, and shORP3#2 and GJB3 protein expression (B) in RT4-TP53DD cells with shScr, shGJB3#1, and shGJB3#2. GAPDH or α-tubulin was used as a loading control. n = 3 separate experiments were conducted. [file 11658_2026_936_MOESM4_ESM.tiff]

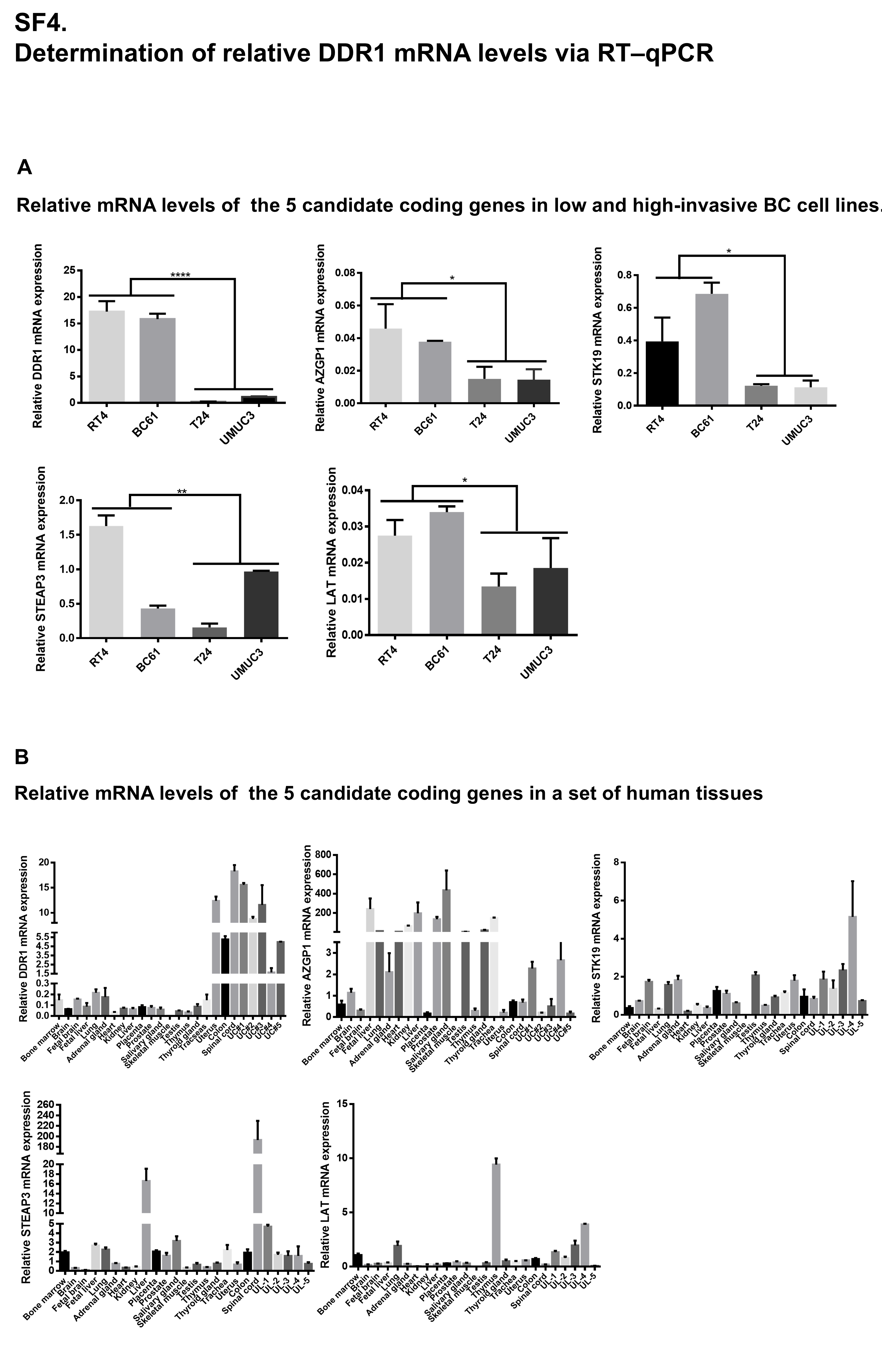

Supplement: Supplementary file 4 — Additional file 4: SF4. Determination of relative DDR1 mRNA levels via RT‒qPCR. A. The bar graph shows the relative DDR1, AZGP1 and STK19, STEAP3, and LAT mRNA levels in non/low-invasive BC cell lines (RT4 and BC61) as well as in highly invasive BC cell lines (T24 and UMUC3). The mRNA levels were normalized to those of GAPDH. n = 3 independent experiments were performed. The error bars represent the means ± SEMs. ? indicates that no significance was determined owing to the low abundance of these genes. B Bar graph showing the relative DDR1, AZGP1 and STK19, STEAP3 and LAT mRNA levels in human tissues. Urothelial cells (UC#1, UC#2, UC#3, UC#4, and UC#5) were isolated from the ureters of five different patients who underwent nephrectomy at Ulm University Hospital. The indicated tissue RNAs were acquired from Clontech (see “Materials and methods” section). The mRNA levels were normalized to those of GAPDH. n = 3 independent experiments were performed. The error bars represent the means ± SEMs. [file 11658_2026_936_MOESM5_ESM.tiff]

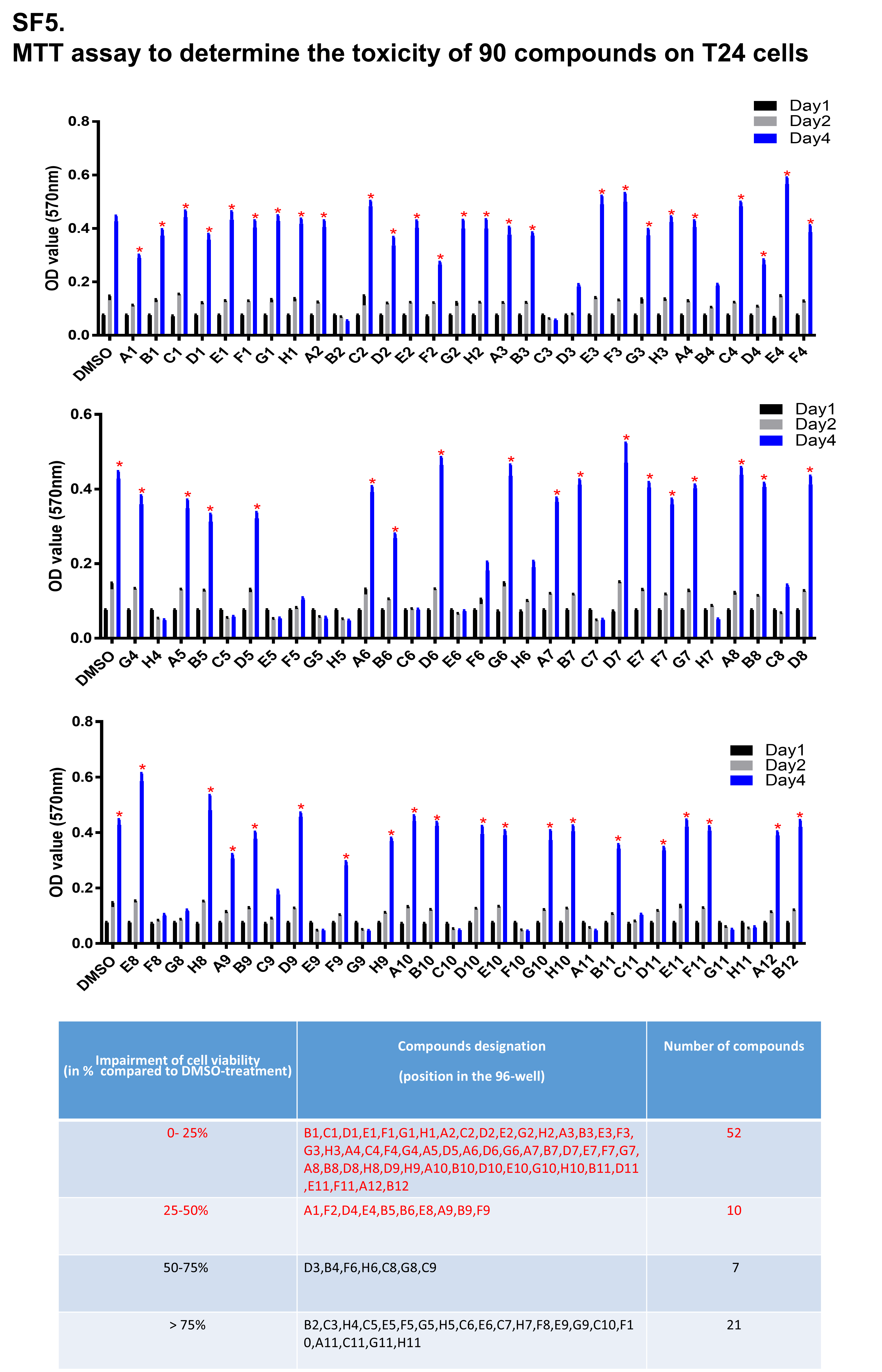

Supplement: Supplementary file 5 — Additional file 5: SF5. MTT assay to determine the toxicity of 90 compounds on T24 cells. (Top) The bar graph shows the results of the 3-(4,5-dimethylthiazol-2-yl)-2,5-diphenyltetrazolium (MTT) assay, which was used to determine the survival of T24 cells after treatment with the indicated compounds at 10 µM concentrations. The compounds are indicated by their location in the 96-well plate. A detailed list of the compounds is provided in Supplementary Table 6 (Supplementary Table ST6). The cells were counted at days 1, 2, and 4 post-seeding. The MTT assay was performed at least in sextuplicate. Red asterisks indicate the 62 compounds that did not significantly impair cell viability within 4 days. (Bottom) Table showing the results of the MTT assay in summary. Impairment of cell viability was quantified and results are shown in percent of viability impairment. For the next step, i.e., for the Boyden chamber experiment, all compounds exhibiting over 50% viability impairment were excluded (black). The majority of the compounds (red) did not or only mildly impair cell viability at 2 or 4 days of treatment and were used to assess their influence on cell migration/invasion using Boyden chamber assay. [file 11658_2026_936_MOESM6_ESM.tiff]

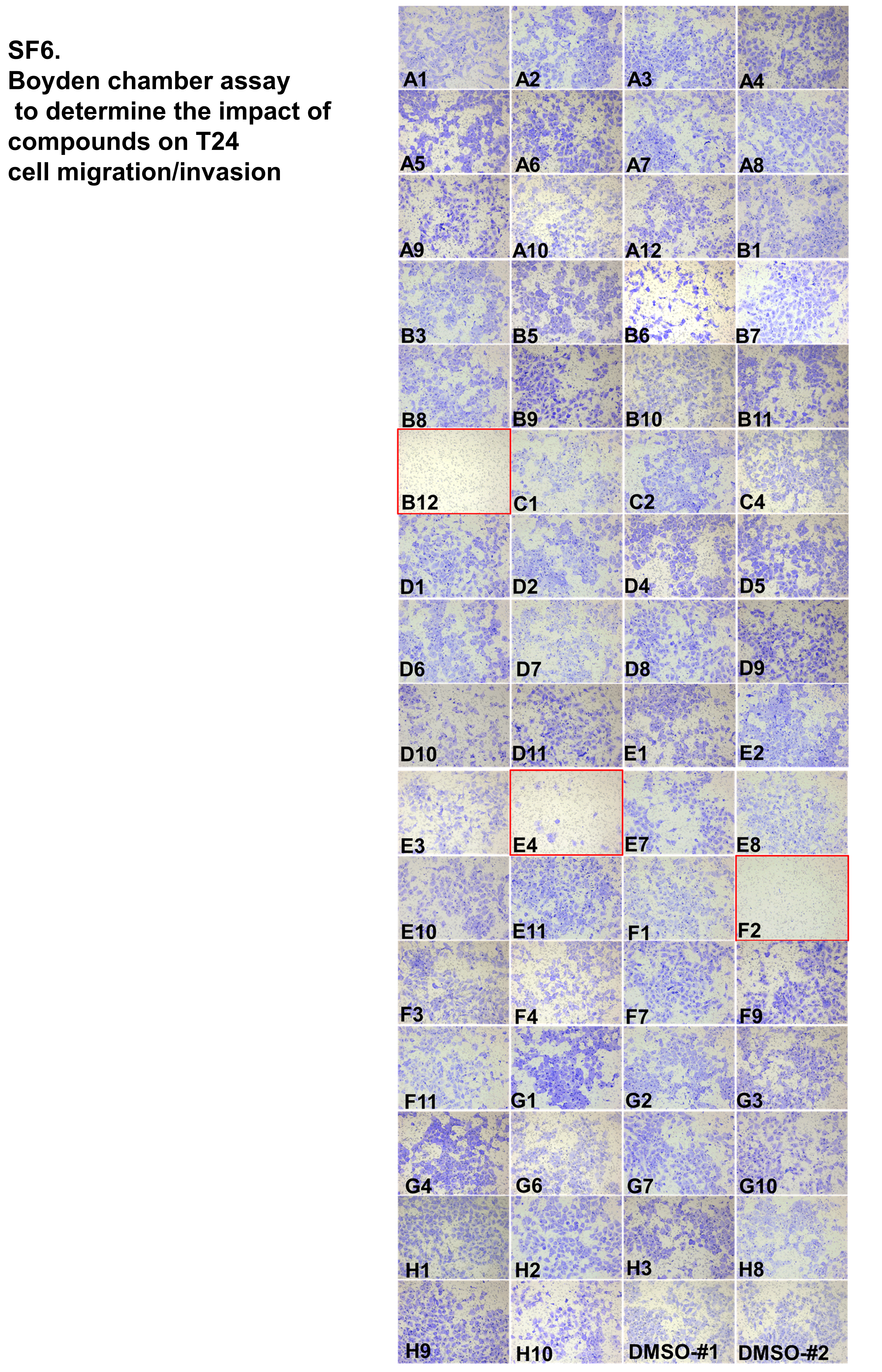

Supplement: Supplementary file 6 — Additional file 6: SF6. Boyden chamber assay to determine the impact of compounds on T24 cell migration/invasion. Representative pictures of the Boyden chamber assay used to assess the impact of the selected 62 compounds on the invasive capacity of T24 cells. Among these, only three compounds (highlighted by red rectangle borders), the matrix metalloprotease 3 (MMP3: position F2 in the 96-well) inhibitor, the cyclooxygenase-2 (COX-2: position B12 in the 96-well) inhibitor, and the antagonist of endothelin receptor type A (ETA: position E4 in the 96-well), impaired the invasive capacity of T24 cells in the Boyden chamber assay. Notably, the results of the Boyden chamber assay were evaluated at 48 h (2 days) after seeding. Importantly, as shown in SF5, cell viability was not impaired at 2 days of treatment with the compounds marked by red asterisks. The compounds are indicated by their location in the 96-well plate. A detailed list of the compounds is provided in Supplementary Table 6 (ST6). [file 11658_2026_936_MOESM7_ESM.tiff]

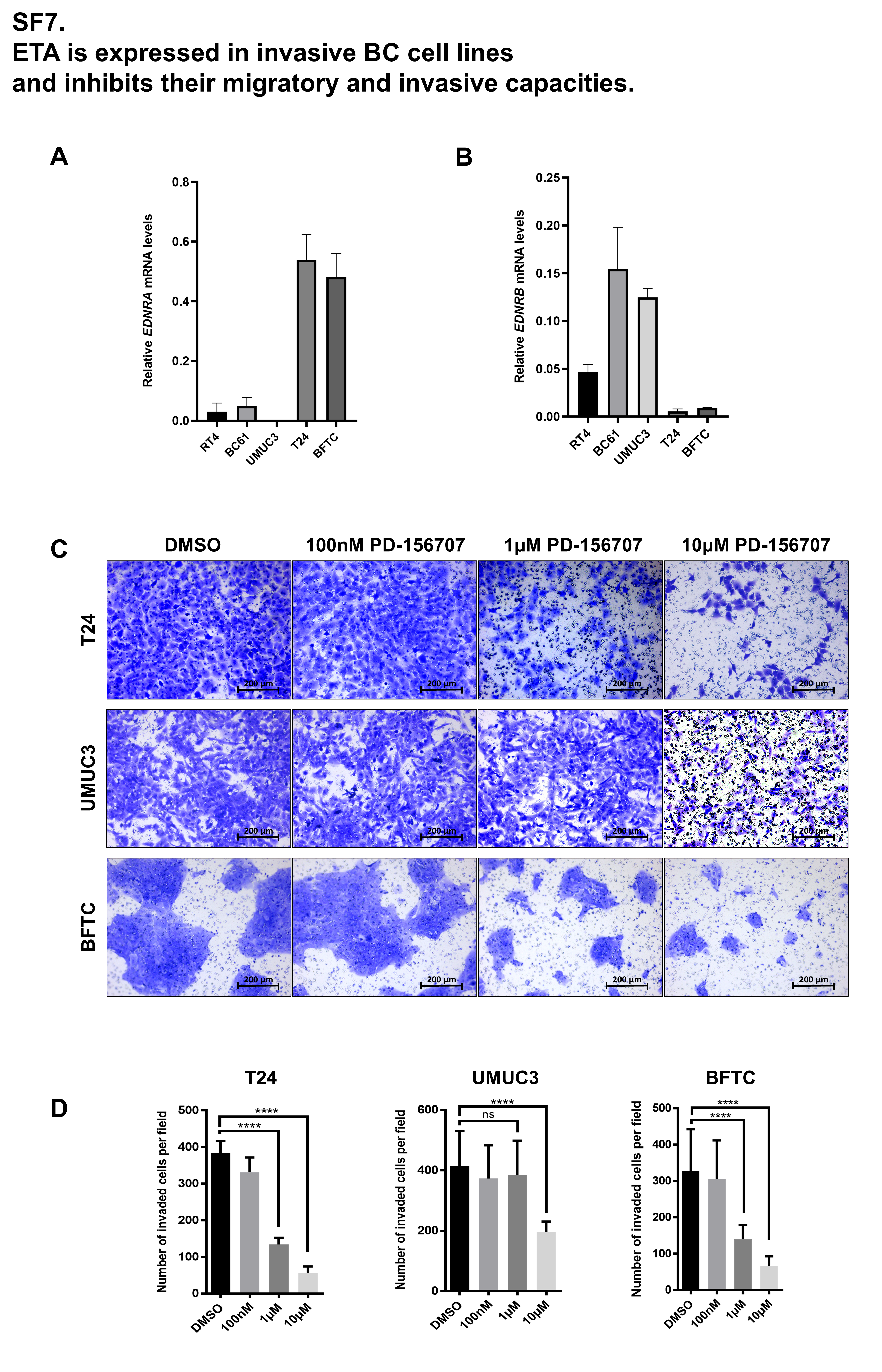

Supplement: Supplementary file 7 — Additional file 7: SF7. ETA is expressed in invasive BC cell lines and inhibits their migratory and invasive capacities. (A) Relative ETA (EDNRA) and (B) ETB (EDNRB) mRNA expression levels quantified by qRT-PCR in RT4, B61, T24, UMUC3, and BFTC-905 cell lines. (C). Depicted exemplary pictures demonstrate the different invasive and migratory capacities of T24, UMUC3, and BFTC-905 lines following treatment with DMSO or the ETA inhibitor (PD-156707) at 100 nM, 1 µM, and 10 µM in the Boyden chamber assay. Representative images of at least n = 3 independent experiments. Images captured at total magnification of 100×. Scale bars: 200 µm. (D) Quantitation of migratory and invasive capacities of T24, UMUC3, and BFTC-905 cell lines following treatment with DMSO or the ETA inhibitor at 100 nM, 1 µM, and 10 µM. Mean ± SEM values from n = 3 independent experiments are shown in the bar graphs. [file 11658_2026_936_MOESM8_ESM.tiff]

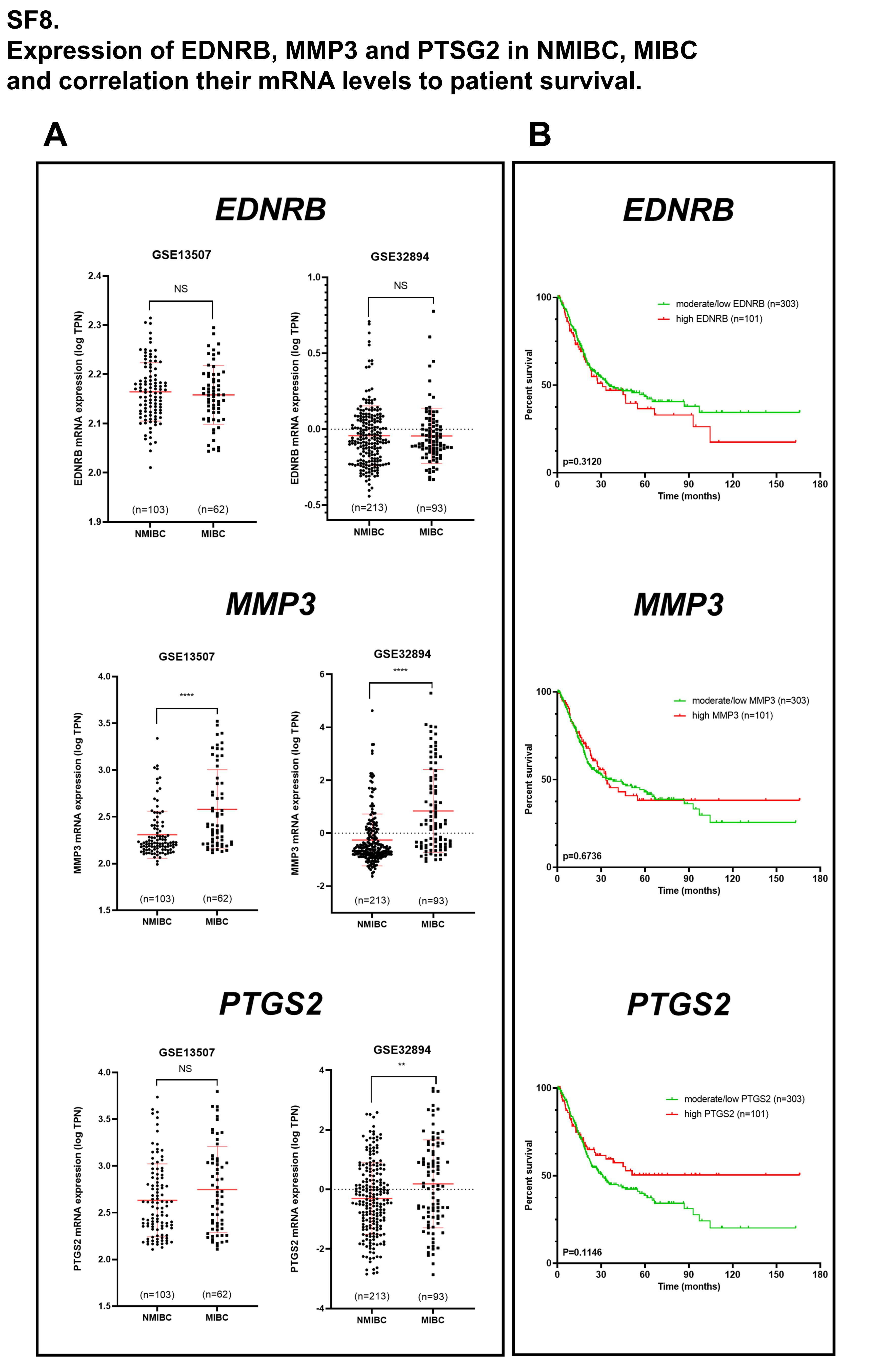

Supplement: Supplementary file 8 — Additional file 8: SF8. Expression of EDNRB, MMP3 and PTGS2 in NMIBC, MIBC and correlation their mRNA levels to patient survival. A. EDNRB (ETA), MMP3, and PTGS2 (COX-2) mRNA levels in NMIBC versus MIBC in GSE13507 and GSE32894 datasets. The microarray datasets GSE13507 and GSE32894 were obtained from the Gene Expression Omnibus (GEO) database (https://www.ncbi.nlm.nih.gov/). GSE13507 contained NMIBC (n = 103) and MIBC (n = 62). GSE32894 contained NMIBC (n = 213) and MIBC (n = 93). Significance was assessed using the unpaired t test. A p-value < 0.05 was considered statistically significant. B Kaplan‒Meier survival curves showing overall survival (OS) for patients with BC in correlation with EDNRB (ETA), MMP3, and PTGS2 (COX-2) mRNA levels, stratified by expression level. The source data [33] were downloaded from the cBioPortal [34, 35], and OS was calculated via PRISM software. Patients were stratified into high (n = 101) and moderate/low (n = 303) EDNRA expression groups using the 75% percentile as the cutoff. Note: OS is plotted against time in months. [file 11658_2026_936_MOESM9_ESM.tiff]

**Fig. 3A**

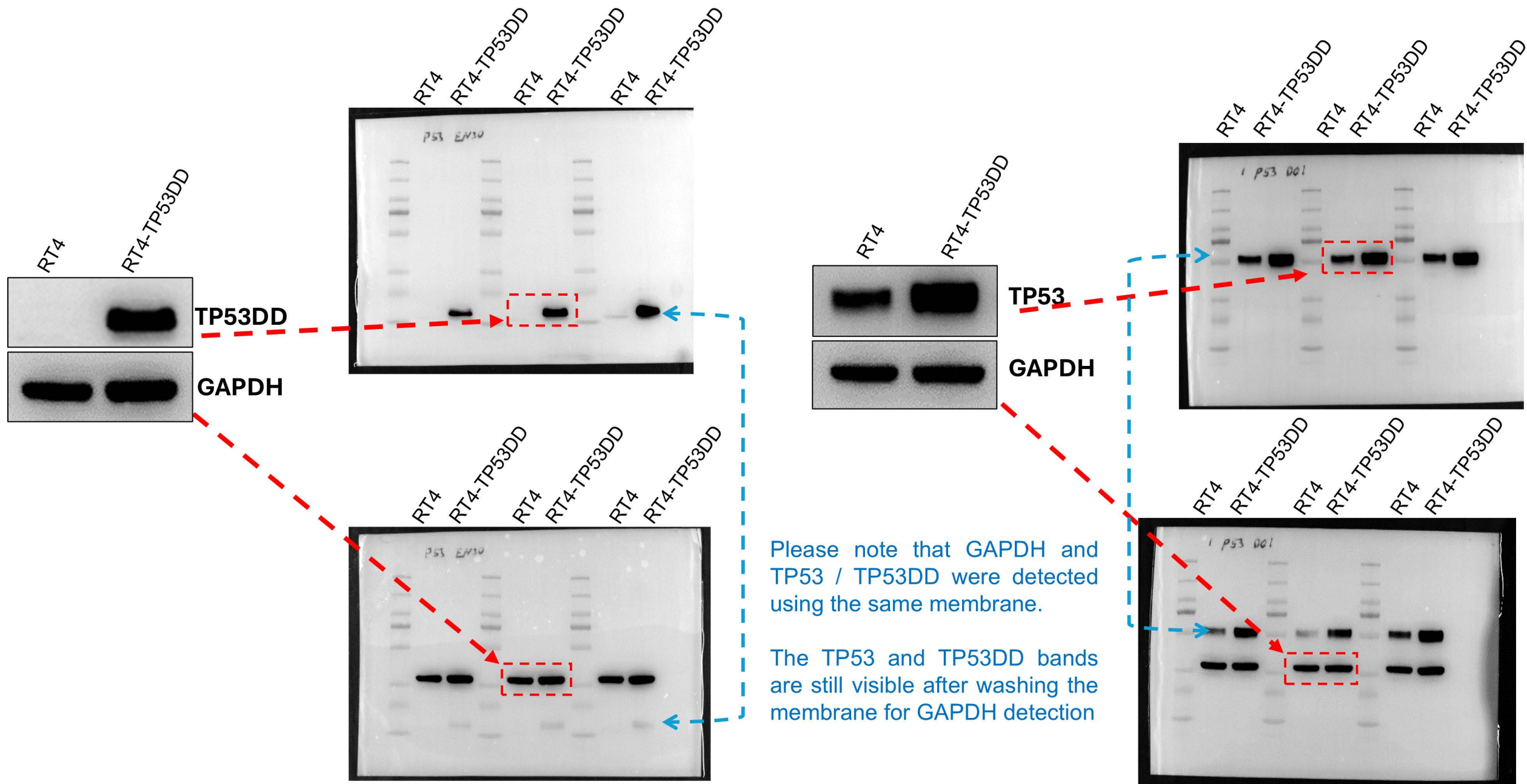

**Fig. 5D**

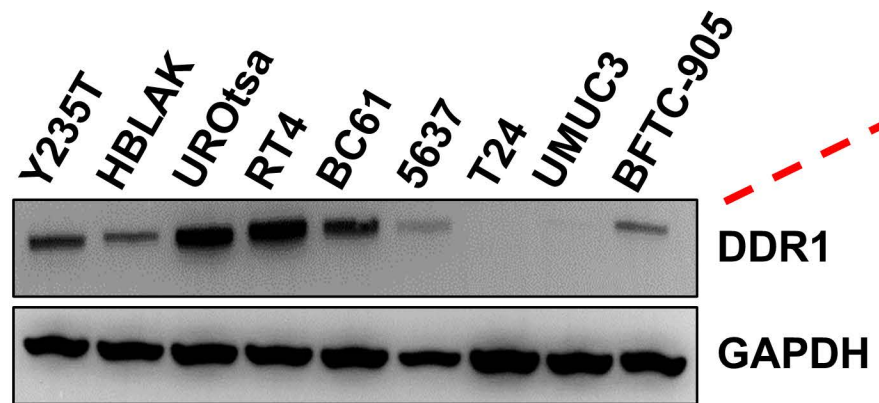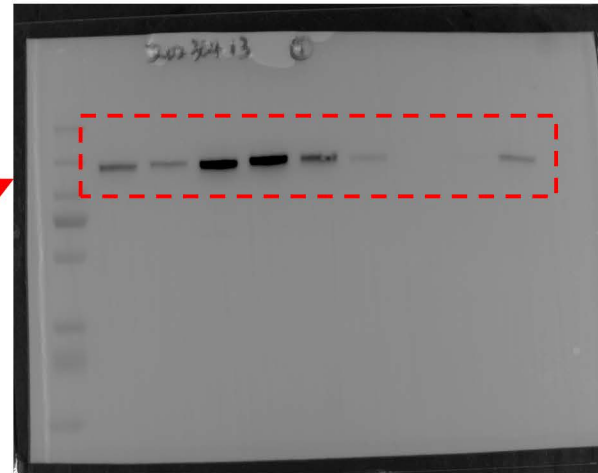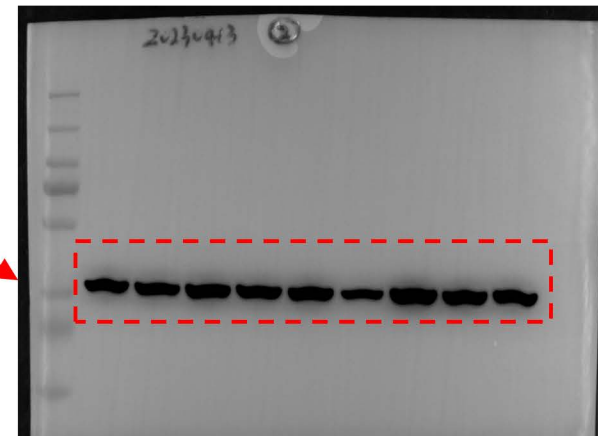

**Fig. 6**

**A**

RT4-TP53DD-gGFP#1  
RT4-TP53DD-gGFP#2  
RT4-TP53DD-gDDR1#1  
RT4-TP53DD-gDDR1#2

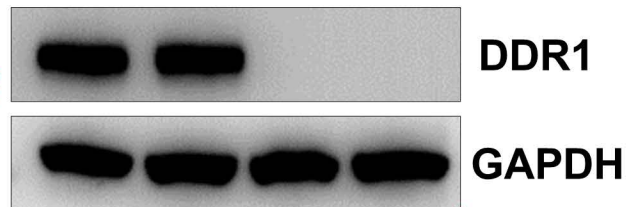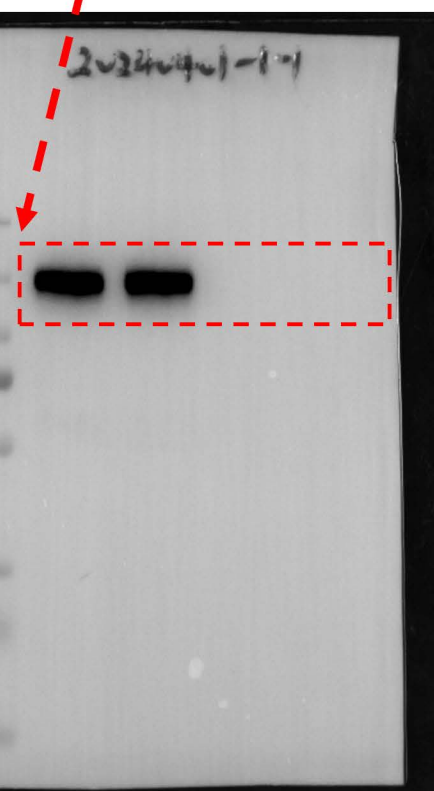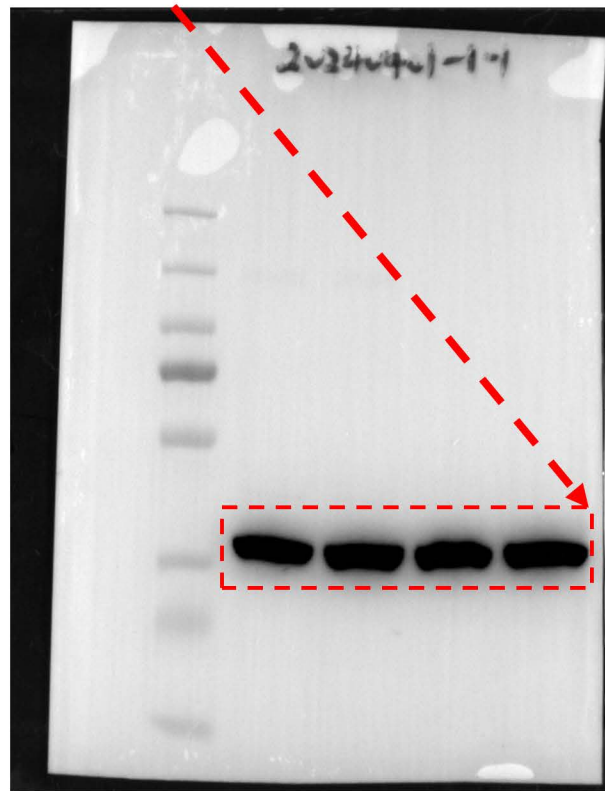

**B**

T24-EV  
T24-DDR1

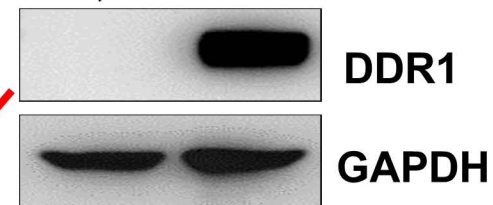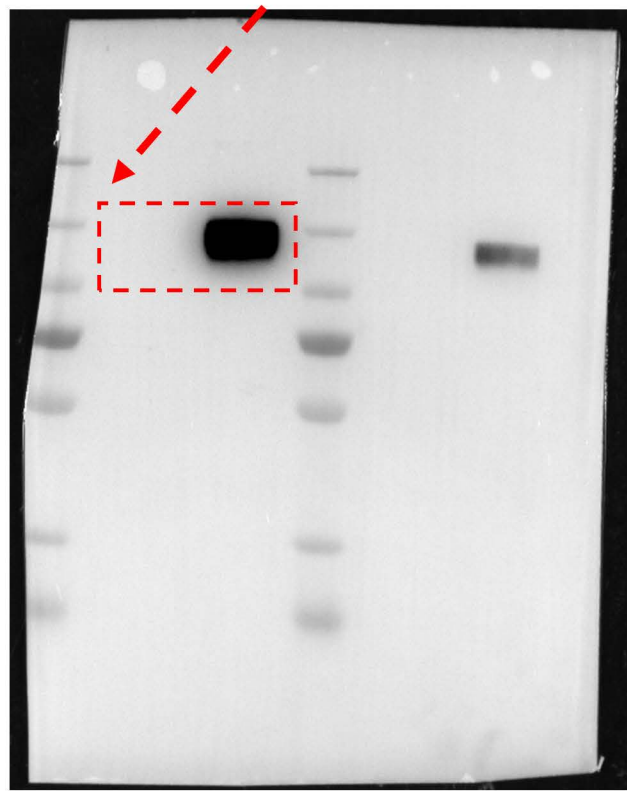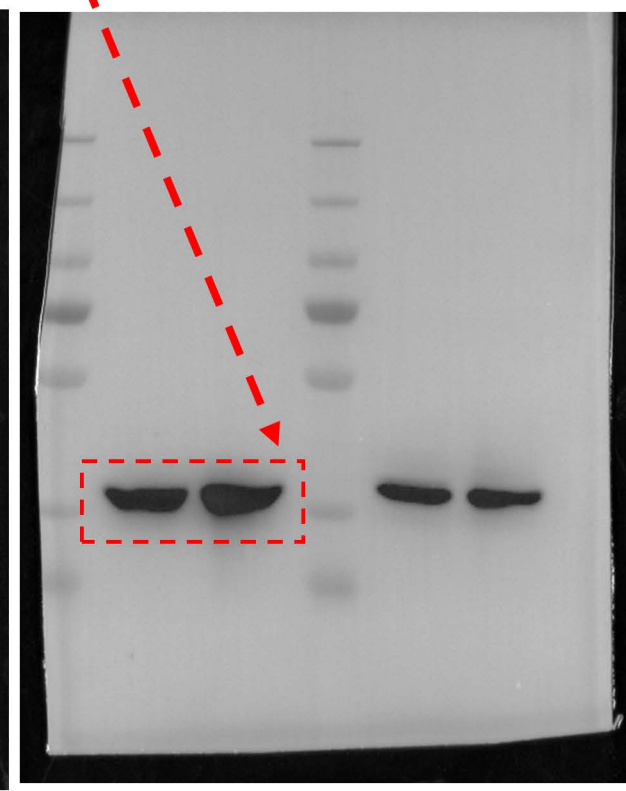

SF2

A

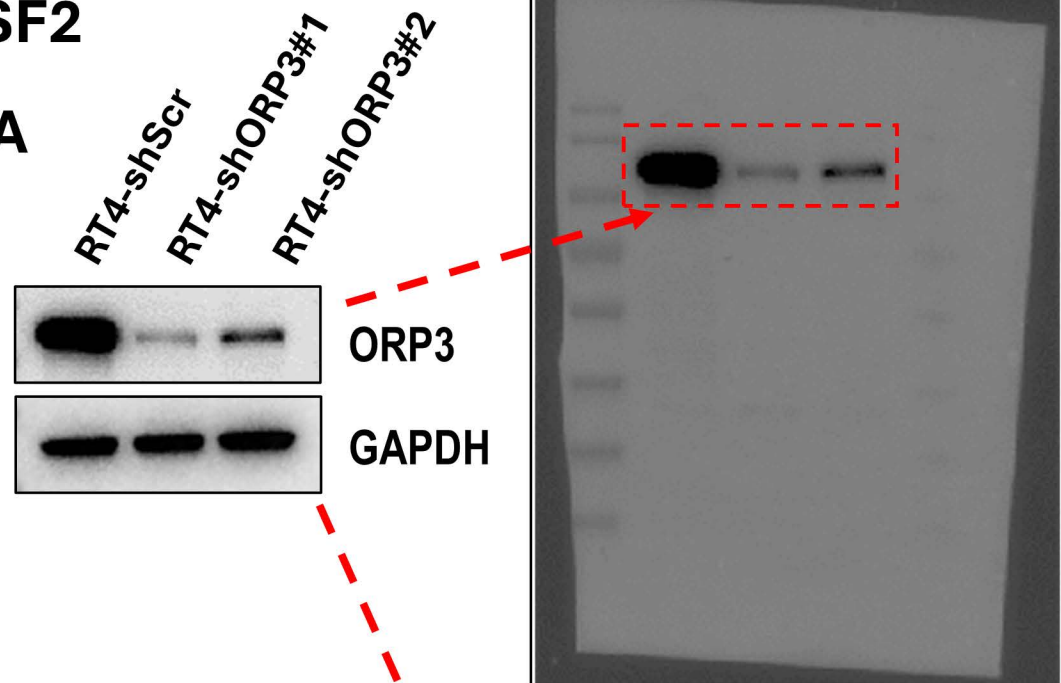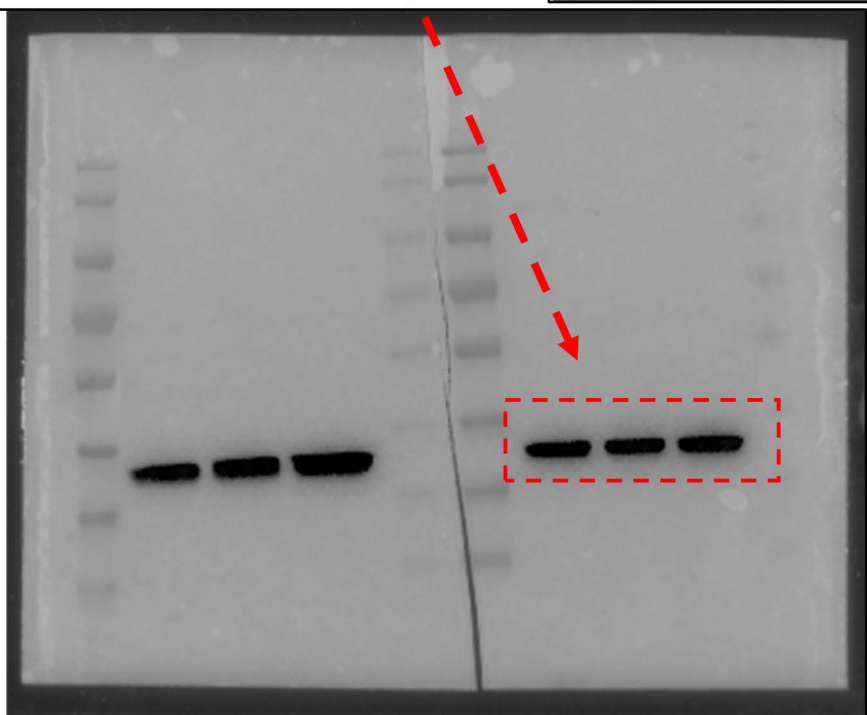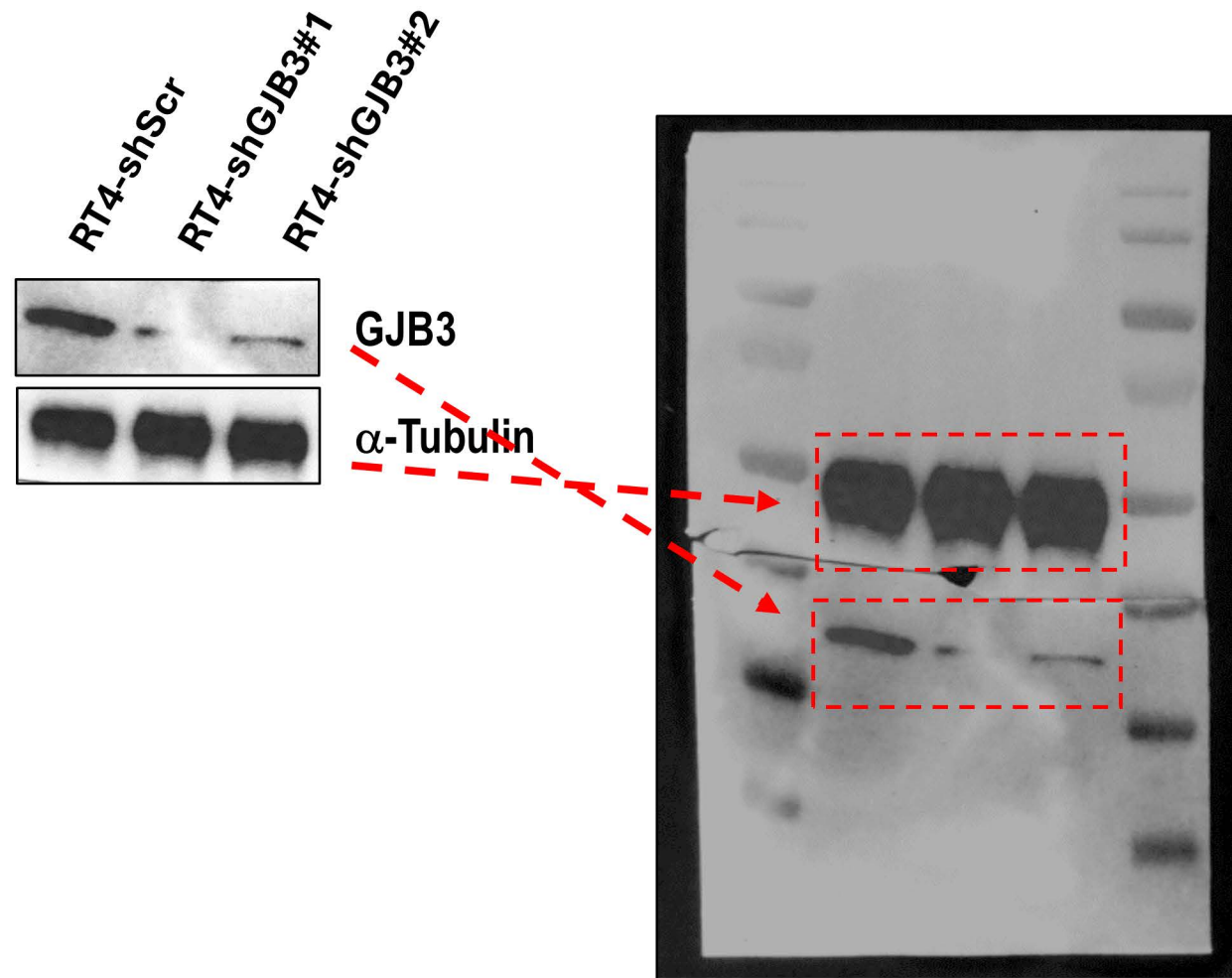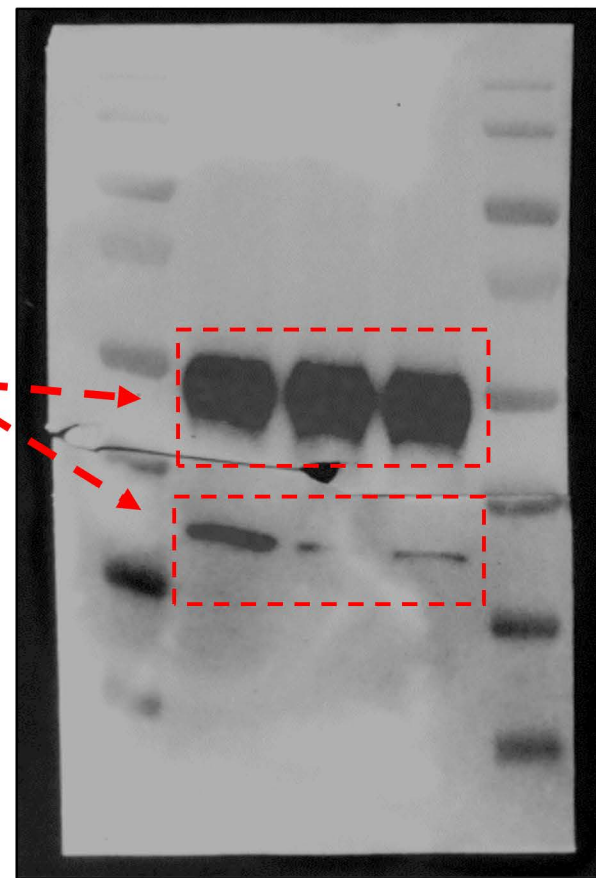

SF2

B

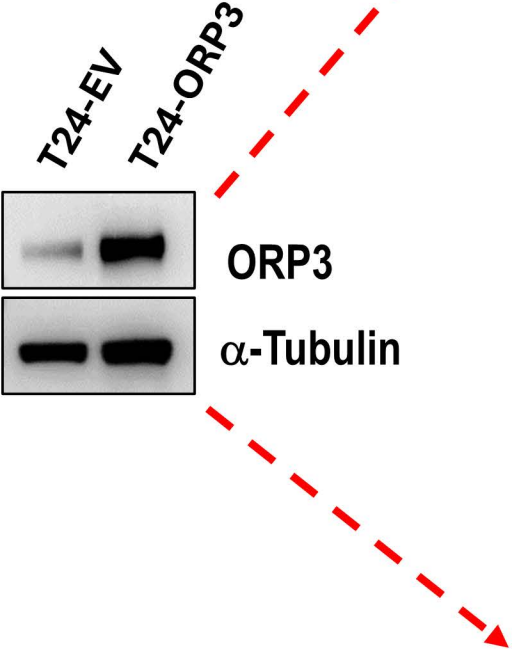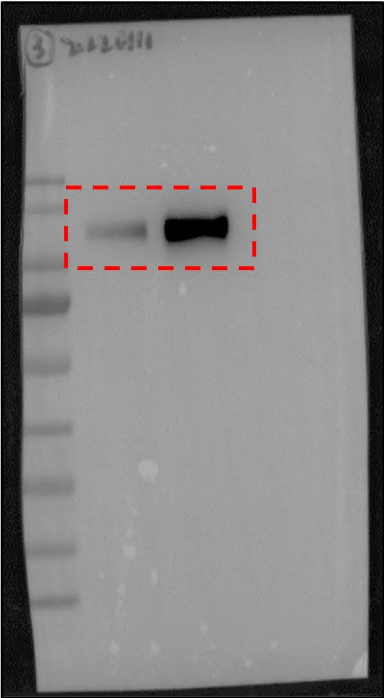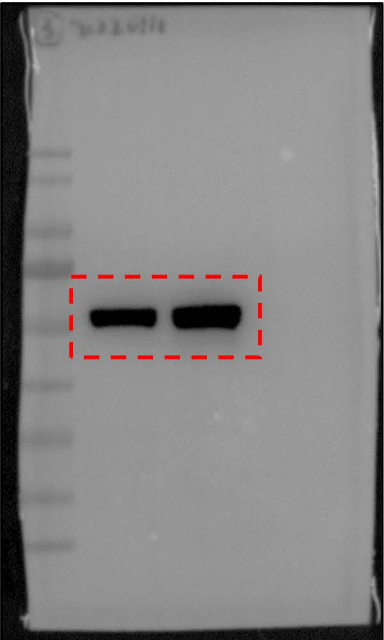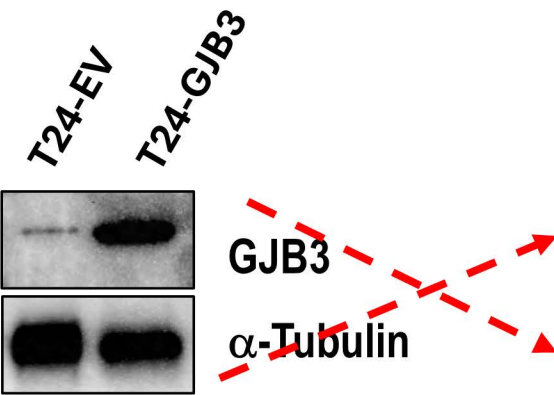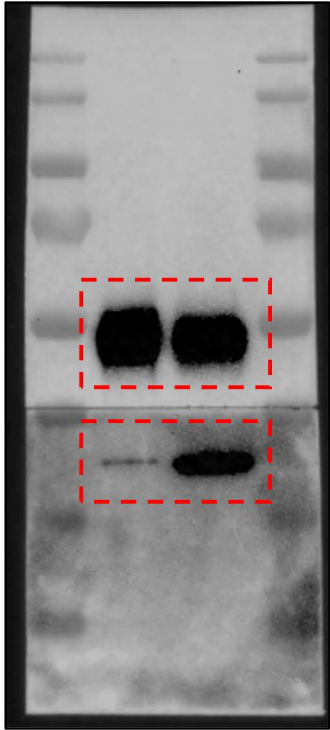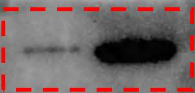

SF3

A

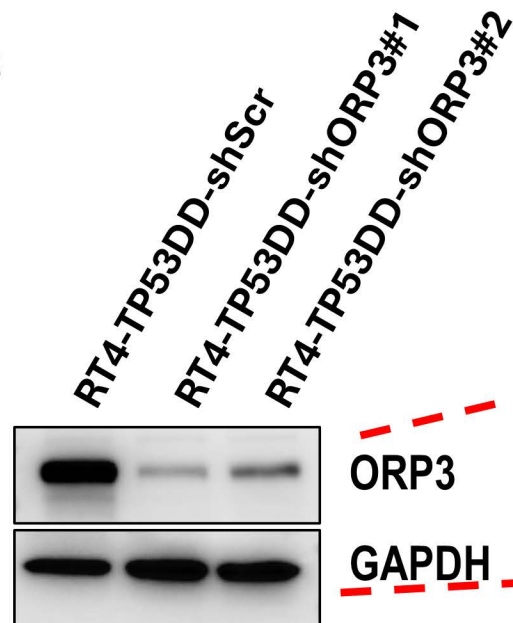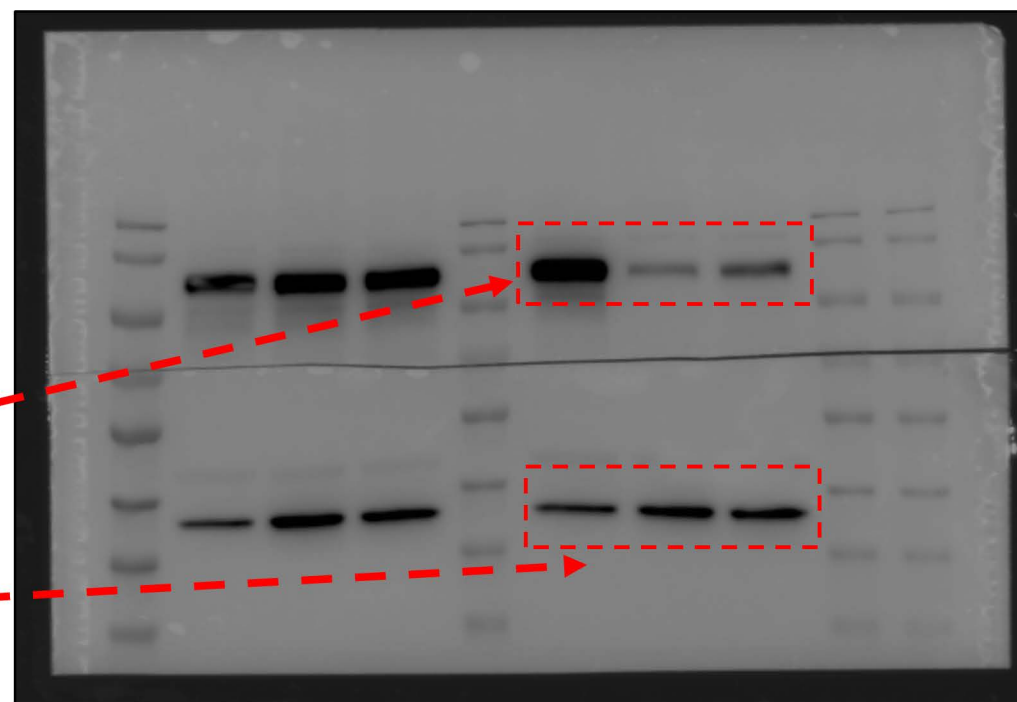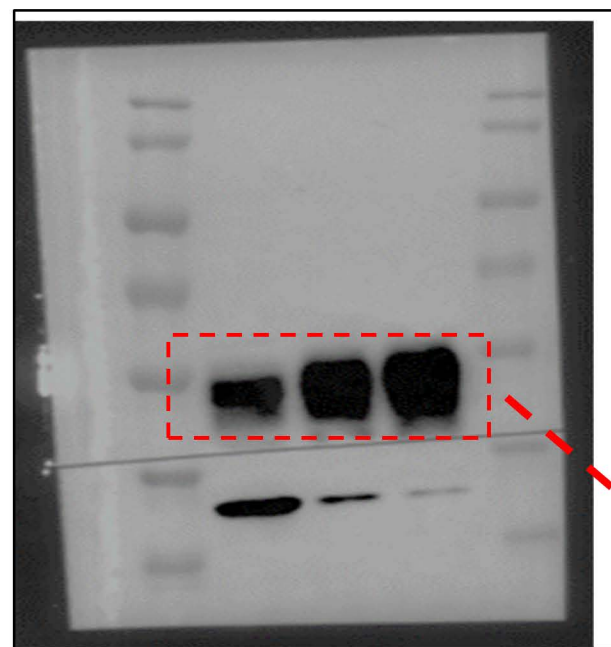

B

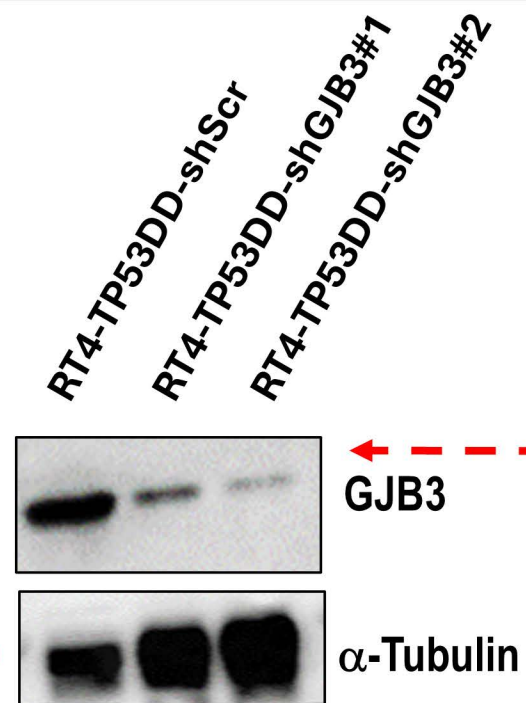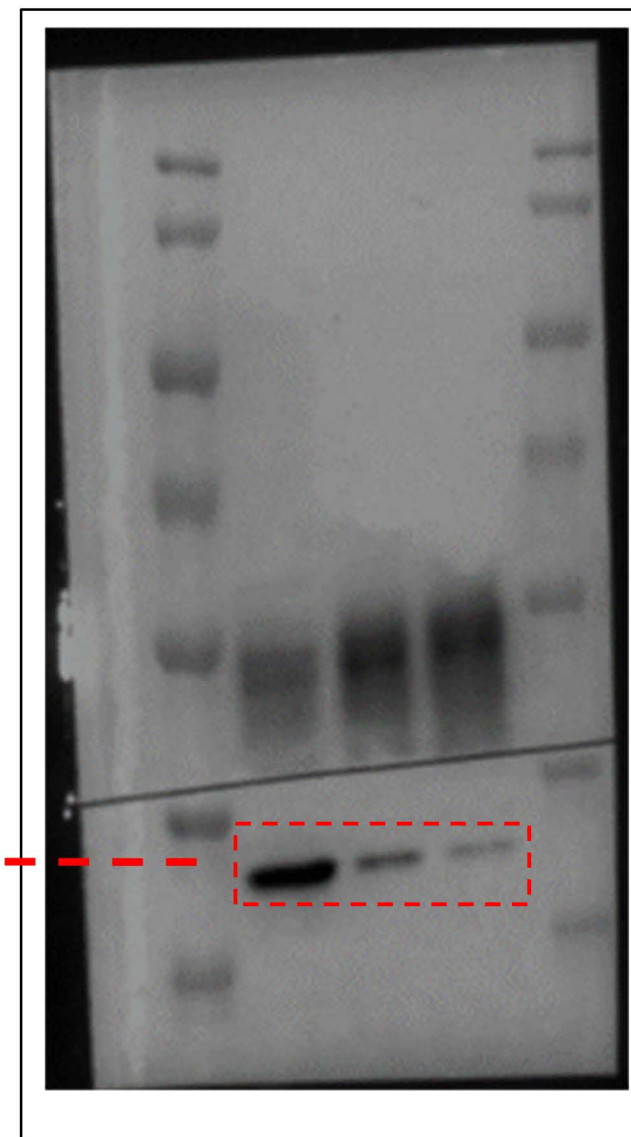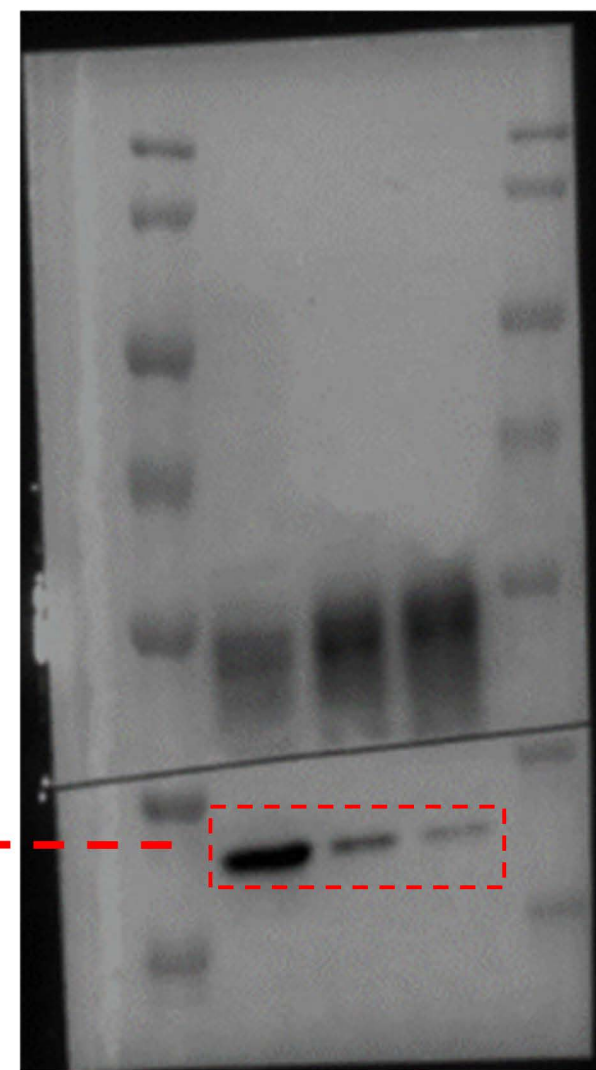

Supplement: Supplementary file 15 — Additional file 15. [file 11658_2026_936_MOESM16_ESM.pdf]
