# Supplementary material for: CRISPR and compound screens in a novel ex vivo tissue model identify DDR1 and ETA as regulators of cancer cell invasion
Source: Cell Mol Biol Lett. 2026 May 6;31:84. doi: 10.1186/s11658-026-00936-6 (PMC13277066; doi:10.1186/s11658-026-00936-6)
Supplement: Supplementary file 10 — Additional file 10: ST2. List of candidate genes with potential roles in cancer cell invasion identified by genome-wide CRISPR-Cas9 library screening using the new ex vivo mouse bladder tissue invasion model. Genes with a previously reported role in cell migration and invasion are marked in green. Notably, the RFLP2 gRNA was found independently in wells 2 and 5 (indicated by red font). The last two columns refer to selected references indicating the role of the candidate genes in cancer and in cancer cell migration/invasion. The reference list is included at the end of ST2. [file 11658_2026_936_MOESM11_ESM.docx]

| **No.** | **Well** | **Gene name** | **Official Full Name** | **Role in Cancer**  (Reference number) | **Role in invasion/metastasis**  (Reference number) |
| --- | --- | --- | --- | --- | --- |
|  |  |  |  |  |  |
| **1** | **1** | **hsa-mir-1231** | **microRNA 123** | [^1^](#_ENREF_1) | [^2^](#_ENREF_2) |
| **2** | **1** | **hsa-mir-6795** | **microRNA 6795** | **No report** | **No report** |
| **3** | 1 | **STEAP3** | **STEAP3 metalloreductase** | [^3^](#_ENREF_3) | [^3^](#_ENREF_3) |
|  |  |  |  |  |  |
| **4** | **2** | **hsa-mir-606** | **microRNA 606** | [^4^](#_ENREF_4) | [^4^](#_ENREF_4) |
| **5** | **2** | **NSMF** | **NMDA receptor synaptonucl. signaling and neuronal migration factor** | **No report** | **No report** |
| **6** | **2** | **FAM192A** | **family with sequence similarity 192 member A** | [^5^](#_ENREF_5) | **No report** |
| **7** | **2 and 5** | **RFPL2** | **ret finger protein like 2** | **No report** | **No report** |
|  |  |  |  |  |  |
| **8** | **4** | **ATP1B1** | **ATPase Na+/K+ transporting subunit beta 1** | [^6^](#_ENREF_6) | **No report** |
| **9** | **4** | **STK19** | **serine/threonine kinase 19** | [^7^](#_ENREF_7)**^,^**[^8^](#_ENREF_8) | [^7^](#_ENREF_7) |
| **10** | **4** | **CALCRL** | **calcitonin receptor like receptor** | [^9^](#_ENREF_9) | **No report** |
| **11** | **4** | **EMP2** | **epithelial membrane protein 2** | [^10^](#_ENREF_10) | [^11^](#_ENREF_11) |
| **12** | **4** | **hsa-mir-1226** | **microRNA 1226** | [^12-15^](#_ENREF_12) | [^14^](#_ENREF_14)**^,^**[^15^](#_ENREF_15) |
|  |  |  |  |  |  |
| **13** | **5** | **hsa-mir-7973-1** | **microRNA 7973-1** | [^16^](#_ENREF_16) | **No report** |
| **14** | **5** | **DDR1** | **discoidin domain receptor tyrosine kinase 1** | [^17-19^](#_ENREF_17) | [^20^](#_ENREF_20)**^,^**[^21^](#_ENREF_21) |
| **15** | **5** | **FMOD** | **fibromodulin** | [^22-24^](#_ENREF_22) | [^25^](#_ENREF_25) |
| **16** | **5** | **CCNL2** | **cyclin L2** | [^26^](#_ENREF_26)**^,^**[^27^](#_ENREF_27) | [^28^](#_ENREF_28) |
| **17** | **5 and 2** | **RFPL2** | **ret finger protein like 2** | **No report** | **No report** |
|  |  |  |  |  |  |
| **18** | **6** | **IL11** | **interleukin 11** | [^29^](#_ENREF_29)**^,^**[^30^](#_ENREF_30) | [^31^](#_ENREF_31) |
| **19** | **6** | **LAT** | **linker for activation of T cells** | [^32^](#_ENREF_32) | **No report** |
| **20** | **6** | **PARS2** | **prolyl-tRNA synthetase 2, mitochondrial** | [^33^](#_ENREF_33) | **No report** |
| **21** | **6** | **TCP10L** | **t-complex 10 like** | [^34^](#_ENREF_34) | **No report** |
| **22** | **6** | **FERMT1** | **FERM domain containing kindlin 1** | [^35^](#_ENREF_35) | [^36^](#_ENREF_36) |
| **23** | **6** | **AZGP1** | **alpha-2-glycoprotein 1, zinc-binding** | [^37^](#_ENREF_37) | [^38^](#_ENREF_38) |
| **24** | **6** | **CASP10** | **caspase 10** | [^39^](#_ENREF_39)**^,^**[^40^](#_ENREF_40) | [^40^](#_ENREF_40) |
|  |  |  |  |  |  |
| **25** | **8** | **hsa-mir-371a** | **microRNA 371a** | [^41^](#_ENREF_41) | [^42^](#_ENREF_42)**^,^**[^43^](#_ENREF_43) |
| **26** | **8** | **ABCA3** | **ATP binding cassette subfamily A member 3** | [^44^](#_ENREF_44) | [^45^](#_ENREF_45) |
| **27** | **8** | **PHYHIP** | **phytanoyl-CoA 2-hydroxylase interacting protein** | [^46^](#_ENREF_46)**^,^**[^47^](#_ENREF_47) | **No report** |
| **28** | **8** | **TMEM18** | **transmembrane protein 18** | [^48^](#_ENREF_48) | [^49^](#_ENREF_49) |
| **29** | **8** | **TPSG1** | **tryptase gamma 1** | [^50^](#_ENREF_50) | [^51^](#_ENREF_51) |
|  |  |  |  |  |  |
| **30** | **10** | **MYT1** | **myelin transcription factor 1** | [^52^](#_ENREF_52) | [^53^](#_ENREF_53) |
| **31** | **10** | **SLC46A2** | **solute carrier family 46 member 2** | [^54^](#_ENREF_54) | **No report** |
| **32** | **10** | **TPBG** | **trophoblast glycoprotein** | [^55^](#_ENREF_55) | [^56^](#_ENREF_56) |
| **33** | **10** | **KIFC2** | **kinesin family member C2** | [^57^](#_ENREF_57) | [^57^](#_ENREF_57) |
| **34** | **10** | **PSENEN** | **presenilin enhancer, gamma-secretase subunit** | [^58^](#_ENREF_58) | **No report** |
| **35** | **10** | **BICD1** | **BICD cargo adaptor 1** | [^59^](#_ENREF_59) | [^60^](#_ENREF_60) |

1 Zhang, J. *et al.* MicroRNA-1231 exerts a tumor suppressor role through regulating the EGFR/PI3K/AKT axis in glioma. *J Neurooncol* **139**, 547-562 (2018). <https://doi.org/10.1007/s11060-018-2903-8>

2 Shang, S. *et al.* Exosomal miRNA-1231 derived from bone marrow mesenchymal stem cells inhibits the activity of pancreatic cancer. *Cancer Med* **8**, 7728-7740 (2019). <https://doi.org/10.1002/cam4.2633>

3 Wang, S., Chen, L. & Liu, W. Matrix stiffness-dependent STEAP3 coordinated with PD-L2 identify tumor responding to sorafenib treatment in hepatocellular carcinoma. *Cancer Cell Int* **22**, 318 (2022). <https://doi.org/10.1186/s12935-022-02634-7>

4 Choi, S. *et al.* MicroRNA‑606 inhibits the growth and metastasis of triple‑negative breast cancer by targeting Stanniocalcin 1. *Oncol Rep* **51** (2024). <https://doi.org/10.3892/or.2023.8661>

5 Jonik-Nowak, B. *et al.* PIP30/FAM192A is a novel regulator of the nuclear proteasome activator PA28γ. *Proc Natl Acad Sci U S A* **115**, E6477-e6486 (2018). <https://doi.org/10.1073/pnas.1722299115>

6 Selvakumar, P. *et al.* Epigenetic silencing of Na,K-ATPase β 1 subunit gene ATP1B1 by methylation in clear cell renal cell carcinoma. *Epigenetics* **9**, 579-586 (2014). <https://doi.org/10.4161/epi.27795>

7 Li, Z. *et al.* Role of high expression levels of STK39 in the growth, migration and invasion of non-small cell type lung cancer cells. *Oncotarget* **7**, 61366-61377 (2016). <https://doi.org/10.18632/oncotarget.11351>

8 Qiu, Y. *et al.* Targeting RAS phosphorylation in cancer therapy: Mechanisms and modulators. *Acta Pharm Sin B* **11**, 3433-3446 (2021). <https://doi.org/10.1016/j.apsb.2021.02.014>

9 Huang, Z. *et al.* Identification and validation of CALCRL-associated prognostic genes in acute myeloid leukemia. *Gene* **809**, 146009 (2022). <https://doi.org/10.1016/j.gene.2021.146009>

10 Dillard, C. *et al.* EMP2 Is a Novel Regulator of Stemness in Breast Cancer Cells. *Mol Cancer Ther* **19**, 1682-1695 (2020). <https://doi.org/10.1158/1535-7163.Mct-19-0850>

11 Ahmat Amin, M. K. B., Shimizu, A. & Ogita, H. The Pivotal Roles of the Epithelial Membrane Protein Family in Cancer Invasiveness and Metastasis. *Cancers (Basel)* **11** (2019). <https://doi.org/10.3390/cancers11111620>

12 Li, X. Y. *et al.* The long noncoding RNA MIR210HG promotes tumor metastasis by acting as a ceRNA of miR-1226-3p to regulate mucin-1c expression in invasive breast cancer. *Aging (Albany NY)* **11**, 5646-5665 (2019). <https://doi.org/10.18632/aging.102149>

13 Mohamadzade, Z., B, M. S., Ghaemi, Z. & Hoseinpour, P. Cell specific tumor suppressor effect of Hsa-miR-1226-3p through downregulation of HER2, PIK3R2, and AKT1 genes. *Int J Biochem Cell Biol* **134**, 105965 (2021). <https://doi.org/10.1016/j.biocel.2021.105965>

14 Lin, F. & Li, R. MiR-1226, mediated by ASCL1, suppresses the progression of non-small cell lung cancer by targeting FGF2. *Bull Cancer* **109**, 424-435 (2022). <https://doi.org/10.1016/j.bulcan.2021.11.017>

15 Liu, W. *et al.* Long noncoding RNA ZFPM2-AS1 regulates ITGB1 by miR-1226-3p to promote cell proliferation and invasion in hepatocellular carcinoma. *Eur Rev Med Pharmacol Sci* **24**, 7612-7620 (2020). <https://doi.org/10.26355/eurrev_202007_22259>

16 Velthut-Meikas, A. *et al.* Research resource: small RNA-seq of human granulosa cells reveals miRNAs in FSHR and aromatase genes. *Mol Endocrinol* **27**, 1128-1141 (2013). <https://doi.org/10.1210/me.2013-1058>

17 Buraschi, S. *et al.* Discoidin Domain Receptor 1 functionally interacts with the IGF-I system in bladder cancer. *Matrix Biol Plus* **6-7**, 100022 (2020). <https://doi.org/10.1016/j.mbplus.2020.100022>

18 Xie, X. *et al.* Discoidin domain receptor 1 activity drives an aggressive phenotype in bladder cancer. *Am J Transl Res* **9**, 2500-2507 (2017).

19 Maitz, K. *et al.* Altered Treg Infiltration after Discoidin Domain Receptor 1 (DDR1) Inhibition and Knockout Promotes Tumor Growth in Lung Adenocarcinoma. *Cancers (Basel)* **15** (2023). <https://doi.org/10.3390/cancers15245767>

20 Xie, X. *et al.* Overexpression of DDR1 Promotes Migration, Invasion, Though EMT-Related Molecule Expression and COL4A1/DDR1/MMP-2 Signaling Axis. *Technol Cancer Res Treat* **19**, 1533033820973277 (2020). <https://doi.org/10.1177/1533033820973277>

21 Liu, X. *et al.* Recombinant humanized collagen type III with high antitumor activity inhibits breast cancer cells autophagy, proliferation, and migration through DDR1. *Int J Biol Macromol* **243**, 125130 (2023). <https://doi.org/10.1016/j.ijbiomac.2023.125130>

22 Dai, Z. *et al.* Innovative molecular subtypes of multiple signaling pathways in colon cancer and validation of FMOD as a prognostic-related marker. *J Cancer Res Clin Oncol* **149**, 13087-13106 (2023). <https://doi.org/10.1007/s00432-023-05163-6>

23 Silva, T. *et al.* Fibromodulin Gene Variants (FMOD) as Potential Biomarkers for Prostate Cancer and Benign Prostatic Hyperplasia. *Dis Markers* **2022**, 5215247 (2022). <https://doi.org/10.1155/2022/5215247>

24 Bettin, A., Reyes, I. & Reyes, N. Gene expression profiling of prostate cancer-associated genes identifies fibromodulin as potential novel biomarker for prostate cancer. *Int J Biol Markers* **31**, e153-162 (2016). <https://doi.org/10.5301/jbm.5000184>

25 Ao, Z. *et al.* Tumor angiogenesis of SCLC inhibited by decreased expression of FMOD via downregulating angiogenic factors of endothelial cells. *Biomed Pharmacother* **87**, 539-547 (2017). <https://doi.org/10.1016/j.biopha.2016.12.110>

26 Li, Q. *et al.* Integrated analysis to identify the AC005154.6/hsa-miR-29c-3p/CCNL2 axis as a novel prognostic biomarker associated with immune infiltration in prostate cancer. *Cancer Cell Int* **22**, 346 (2022). <https://doi.org/10.1186/s12935-022-02779-5>

27 Mo, X. B. *et al.* A novel long non-coding RNA, lnc-RNU12, influences the T-cell cycle via c-JUN and CCNL2 in rheumatoid arthritis. *Rheumatology (Oxford)* **62**, 1955-1963 (2023). <https://doi.org/10.1093/rheumatology/keac553>

28 Chen, S. *et al.* CLK1/SRSF5 pathway induces aberrant exon skipping of METTL14 and Cyclin L2 and promotes growth and metastasis of pancreatic cancer. *J Hematol Oncol* **14**, 60 (2021). <https://doi.org/10.1186/s13045-021-01072-8>

29 Putoczki, T. L. & Ernst, M. IL-11 signaling as a therapeutic target for cancer. *Immunotherapy* **7**, 441-453 (2015). <https://doi.org/10.2217/imt.15.17>

30 Ernst, M. & Putoczki, T. L. Molecular pathways: IL11 as a tumor-promoting cytokine-translational implications for cancers. *Clin Cancer Res* **20**, 5579-5588 (2014). <https://doi.org/10.1158/1078-0432.Ccr-13-2492>

31 Johnstone, C. N., Chand, A., Putoczki, T. L. & Ernst, M. Emerging roles for IL-11 signaling in cancer development and progression: Focus on breast cancer. *Cytokine Growth Factor Rev* **26**, 489-498 (2015). <https://doi.org/10.1016/j.cytogfr.2015.07.015>

32 Balagopalan, L. *et al.* Enhanced T-cell signaling in cells bearing linker for activation of T-cell (LAT) molecules resistant to ubiquitylation. *Proc Natl Acad Sci U S A* **108**, 2885-2890 (2011). <https://doi.org/10.1073/pnas.1007098108>

33 Wang, J. *et al.* Multi-Omics Database Analysis of Aminoacyl-tRNA Synthetases in Cancer. *Genes (Basel)* **11** (2020). <https://doi.org/10.3390/genes11111384>

34 Shen, S. *et al.* TCP10L negatively regulates alpha-fetoprotein expression in hepatocellular carcinoma. *BMB Rep* **53**, 431-436 (2020). <https://doi.org/10.5483/BMBRep.2020.53.8.008>

35 Fan, H., Zhang, S., Zhang, Y., Liang, W. & Cao, B. FERMT1 promotes gastric cancer progression by activating the NF-κB pathway and predicts poor prognosis. *Cancer Biol Ther* **21**, 815-825 (2020). <https://doi.org/10.1080/15384047.2020.1792218>

36 Liu, C. C. *et al.* FERMT1 mediates epithelial-mesenchymal transition to promote colon cancer metastasis via modulation of β-catenin transcriptional activity. *Oncogene* **36**, 1779-1792 (2017). <https://doi.org/10.1038/onc.2016.339>

37 Yun, H. *et al.* Degradation of AZGP1 suppresses apoptosis and facilitates cholangiocarcinoma tumorigenesis via TRIM25. *J Cell Mol Med* **28**, e18104 (2024). <https://doi.org/10.1111/jcmm.18104>

38 Liu, J. *et al.* AZGP1 inhibits soft tissue sarcoma cells invasion and migration. *BMC Cancer* **18**, 89 (2018). <https://doi.org/10.1186/s12885-017-3962-5>

39 Consonni, F. *et al.* Study of the potential role of CASPASE-10 mutations in the development of autoimmune lymphoproliferative syndrome. *Cell Death Dis* **15**, 315 (2024). <https://doi.org/10.1038/s41419-024-06679-6>

40 Kumari, R., Deshmukh, R. S. & Das, S. Caspase-10 inhibits ATP-citrate lyase-mediated metabolic and epigenetic reprogramming to suppress tumorigenesis. *Nat Commun* **10**, 4255 (2019). <https://doi.org/10.1038/s41467-019-12194-6>

41 Belge, G. *et al.* Graded expression of microRNA-371a-3p in tumor tissues, contralateral testes, and in serum of patients with testicular germ cell tumor. *Oncotarget* **11**, 1462-1473 (2020). <https://doi.org/10.18632/oncotarget.27565>

42 Wang, H. *et al.* MiR-371 promotes proliferation and metastasis in hepatocellular carcinoma by targeting PTEN. *BMB Rep* **52**, 312-317 (2019). <https://doi.org/10.5483/BMBRep.2019.52.5.155>

43 Guo, H. *et al.* MicroRNA-371a-3p promotes progression of gastric cancer by targeting TOB1. *Cancer Lett* **443**, 179-188 (2019). <https://doi.org/10.1016/j.canlet.2018.11.021>

44 Schimanski, S. *et al.* Expression of the lipid transporters ABCA3 and ABCA1 is diminished in human breast cancer tissue. *Horm Metab Res* **42**, 102-109 (2010). <https://doi.org/10.1055/s-0029-1241859>

45 Song, M., Gao, L., Zang, J. & Xing, X. ABCA3, a tumor suppressor gene, inhibits the proliferation, migration and invasion of lung adenocarcinoma by regulating the epithelial‑mesenchymal transition process. *Oncol Lett* **26**, 420 (2023). <https://doi.org/10.3892/ol.2023.14006>

46 Yamamoto, F. & Yamamoto, M. Identification of genes that exhibit changes in expression on the 8p chromosomal arm by the Systematic Multiplex RT-PCR (SM RT-PCR) and DNA microarray hybridization methods. *Gene Expr* **14**, 217-227 (2008). <https://doi.org/10.3727/105221608786883816>

47 Li, J., Pu, K., Li, C., Wang, Y. & Zhou, Y. A Novel Six-Gene-Based Prognostic Model Predicts Survival and Clinical Risk Score for Gastric Cancer. *Front Genet* **12**, 615834 (2021). <https://doi.org/10.3389/fgene.2021.615834>

48 Ha, M. *et al.* TMEM18: A Novel Prognostic Marker in Acute Myeloid Leukemia. *Acta Haematol* **140**, 71-76 (2018). <https://doi.org/10.1159/000492742>

49 Huang, T., Huang, X., Nie, Y., Shi, X. & Shu, C. A Combined Effect of Expression Levels of Obesity-Related Genes and Clinical Factors on Cancer Survival Rate. *Biomed Res Int* **2020**, 8838676 (2020). <https://doi.org/10.1155/2020/8838676>

50 Pu, S. *et al.* Identification of necroptosis-related subtypes and prognosis model in triple negative breast cancer. *Front Immunol* **13**, 964118 (2022). <https://doi.org/10.3389/fimmu.2022.964118>

51 Liu, J. *et al.* Identification of liver metastasis-associated genes in human colon carcinoma by mRNA profiling. *Chin J Cancer Res* **30**, 633-646 (2018). <https://doi.org/10.21147/j.issn.1000-9604.2018.06.08>

52 Choi, H. S., Bode, A. M., Shim, J. H., Lee, S. Y. & Dong, Z. c-Jun N-terminal kinase 1 phosphorylates Myt1 to prevent UVA-induced skin cancer. *Mol Cell Biol* **29**, 2168-2180 (2009). <https://doi.org/10.1128/mcb.01508-08>

53 Melhuish, T. A. *et al.* Myt1 and Myt1l transcription factors limit proliferation in GBM cells by repressing YAP1 expression. *Biochim Biophys Acta Gene Regul Mech* **1861**, 983-995 (2018). <https://doi.org/10.1016/j.bbagrm.2018.10.005>

54 Kim, K. Y. *et al.* Expression Analyses Revealed Thymic Stromal Co-Transporter/Slc46A2 Is in Stem Cell Populations and Is a Putative Tumor Suppressor. *Mol Cells* **38**, 548-561 (2015). <https://doi.org/10.14348/molcells.2015.0044>

55 He, P. *et al.* Trophoblast glycoprotein promotes pancreatic ductal adenocarcinoma cell metastasis through Wnt/planar cell polarity signaling. *Mol Med Rep* **12**, 503-509 (2015). <https://doi.org/10.3892/mmr.2015.3412>

56 Spencer, H. L. *et al.* Role of TPBG (Trophoblast Glycoprotein) Antigen in Human Pericyte Migratory and Angiogenic Activity. *Arterioscler Thromb Vasc Biol* **39**, 1113-1124 (2019). <https://doi.org/10.1161/atvbaha.119.312665>

57 Liu, X. *et al.* The kinesin-14 family motor protein KIFC2 promotes prostate cancer progression by regulating p65. *J Biol Chem* **299**, 105253 (2023). <https://doi.org/10.1016/j.jbc.2023.105253>

58 Chen, K. *et al.* Immunological and prognostic analysis of PSENEN in low-grade gliomas: An immune infiltration-related prognostic biomarker. *Front Mol Neurosci* **15**, 933855 (2022). <https://doi.org/10.3389/fnmol.2022.933855>

59 Dong, Y. *et al.* Baicalein promotes KDM4E to induce BICD1 and inhibit triple-negative breast cancer progression by blocking PAR1 signaling. *Mol Carcinog* **63**, 1288-1302 (2024). <https://doi.org/10.1002/mc.23724>

60 Jiang, Y. *et al.* BICD1 functions as a prognostic biomarker and promotes hepatocellular carcinoma progression. *Pathol Res Pract* **216**, 152858 (2020). <https://doi.org/10.1016/j.prp.2020.152858>
