# Supplementary material for: CRISPR and compound screens in a novel ex vivo tissue model identify DDR1 and ETA as regulators of cancer cell invasion
Source: Cell Mol Biol Lett. 2026 May 6;31:84. doi: 10.1186/s11658-026-00936-6 (PMC13277066; doi:10.1186/s11658-026-00936-6)
Supplement: Supplementary file 12 — Additional file 12: ST4. The applications, advantages, and disadvantages of the Boyden chamber, ex vivo porcine bladder, and EXTIM are compared. [file 11658_2026_936_MOESM13_ESM.docx]

| **N.A. :Not available** | **Boyden chamber**  **model** | **Ex vivo porcine**  **bladder model** | **EXTIM** |
| --- | --- | --- | --- |
| **Application** | **Migration/invasion** | **Invasion** | **Invasion** |
| **Characteristics** | **Collagen matrix**  **Cell culture** | **De-epithelized bladder tissue**  ***ex vivo*** | **Whole bladder tissue**  ***ex vivo*** |
| **Operational complexity** | **Simple** | **Demanding** | **Moderate** |
| **Contamination** | **Rarely** | **Occasionally** | **Rarely** |
| **Suitability for genome-wide screens** | **Suitable**  **for migration/invasion related**  **approaches** | **Unsuitable** | **Suitable for tissue invasion related approaches** |
| **Background genetic variation among samples** | **N.A.** | **Genetic variation** | **Inbred mice with close**  **genetic homogeneity** |
| **Combination with transgenic animal models** | **N.A.** | **N.A.** | **Suitable** |

**ST4. Comparison of Boyden chamber model, *ex vivo* porcine bladder model and EXTIM.**

**N.A. : Not available**
